# The first microseconds of the life of excited heptamethine cyanine revealed by femtosecond stimulated Raman spectroscopy

**DOI:** 10.1038/s42004-025-01850-2

**Published:** 2025-12-15

**Authors:** Gabriel Glotz, Jan Polena, Nasrulla Majid Khan, Atripan Mukherjee, Miroslav Kloz, Petr Slavíček, Petr Klán

**Affiliations:** 1 Department of Chemistry, Faculty of Science, Masaryk University, Brno, Czech Republic; 2 RECETOX, Faculty of Science, Masaryk University, Brno, Czech Republic; 3 Department of Physical Chemistry, University of Chemistry and Technology, Prague, Prague, Czech Republic; 4 The Extreme Light Infrastructure Facility ERIC, Dolni Břežany, Czech Republic

**Keywords:** Photochemistry, Computational chemistry

## Abstract

Heptamethine cyanines are a well-known class of organic near-infrared (NIR) fluorophores that play an indispensable role in chemistry and biology. Despite their ubiquity, the underlying photophysical and photochemical dynamics triggered by excitation remain surprisingly elusive. In this study, we investigated a prototypical heptamethine cyanine (**Cy7**) using femtosecond stimulated Raman spectroscopy. Combining transient Raman spectra with quantum chemical calculations allowed us to develop a comprehensive picture of the species produced during **Cy7** excitation and their behavior on the fs to sub-ms time scale. We have unambiguously identified the excited singlet and triplet states and the resulting configurational photoisomers using clearly distinguishable Raman shifts. We also reveal solvent-mediated relaxation channels, in particular ultrafast photoinduced electron transfer to dissolved oxygen, generating cyanine radical dication in addition to superoxide. Together, these insights provide a coherent mechanistic framework for **Cy7** photodynamics and provide design guidance for next-generation NIR probes.

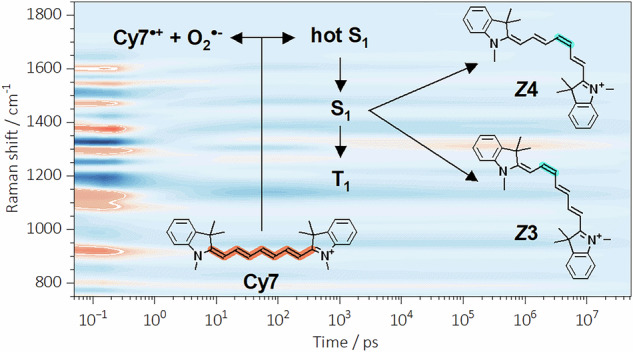

## Abstract (web summary)

Cyanines are a well-known class of organic, near-infrared fluorophores, however, their photoexcited dynamics are not well understood. In this study, the authors investigated the photochemical behavior of a prototypical heptamethine cyanine derivative using transient Raman spectroscopy and quantum chemical calculations, observing ultrafast electron transfer between the excited cyanine and oxygen and identifying key intermediates and products, including the excited states and photoisomers.

## Introduction

Cyanine chromophores from the polymethine dye family are among the best-known synthetic pigments^1,2^. Cyanine dyes were originally widely used in silver halide photography^3^ and LASER mode locking^4^ but have recently found widespread use in biochemical and medical research^5^ primarily due to their remarkable spectroscopic properties. Heptamethine cyanine dyes have become the fluorophore of choice for labeling biological molecules^6^, single-molecule spectroscopy^7,8^, optical cancer imaging and targeted therapy^9–12^, biosensors^13,14^, and drug delivery and photodynamic therapy^15–17^. Despite their widespread use, the photophysical and photochemical properties of heptamethine cyanine dyes are poorly understood. After excitation to the excited singlet state, the primary process is configurational photoisomerization, which leads to *Z*-isomers. This isomerization occurs via a spectroscopically dark twisted intermediate^18^. The relative efficiency of photoisomerization compared to other deactivation processes depends on the substituents, temperature, and solvent viscosity, and the lifetime of the photoisomers^19,20^. The formation of these photoisomers contributes to the so-called blinking of cyanine fluorescence, and they are referred to as dark states^21,22^. Photoisomerization is considered one of the predominant nonradiative deactivation pathways of the cyanine excited singlet state. However, Štacko^23^ and Schnermann^24^ recently challenged this view by showing that the fluorescence quantum yield is mainly reduced by fast nonradiative deactivation pathways of the S_1_ state significantly associated with various C–H vibrational modes of heterocyclic rings. In addition, the formation of the triplet state also contributes to the fluorescence blinking. The triplet state forms via intersystem crossing (ISC) with very low quantum yields, typically with a lifetime in the sub-millisecond range^25,26^. Photoisomerization and triplet formation processes have been extensively studied using transient absorption (TA) spectroscopy^27–29^. Unfortunately, the large overlap between the transient absorption spectra of the triplet state and the four possible mono *Z*-photoisomers in heptamethine cyanine dyes has prevented further elucidation thus far^30^. Since TA spectroscopy cannot distinguish between the possible *Z*-photoisomers, the question of which photoisomer is formed has largely been the subject of computational studies^31,32^.

Femtosecond-stimulated Raman (FSR) spectroscopy is one of the fastest-developing ultrafast vibrational spectroscopy techniques^33–39^. It provides information about the vibrational structure with high temporal and spectral resolution. Because Raman spectroscopy is very useful for determining C = C bond configurations, FSR spectroscopy has been used to study the photoisomerization of retinal^40,41^, linear carotenoids^42–44^, and phytochrome^45^, to describe the photoreception dynamics of photoexcited myoglobin^46^ in the photoactive yellow protein^47^, and most recently, to detect the perpendicular “phantom” state formed during the photoisomerization of stilbene^48^.

**Fig. 1:**
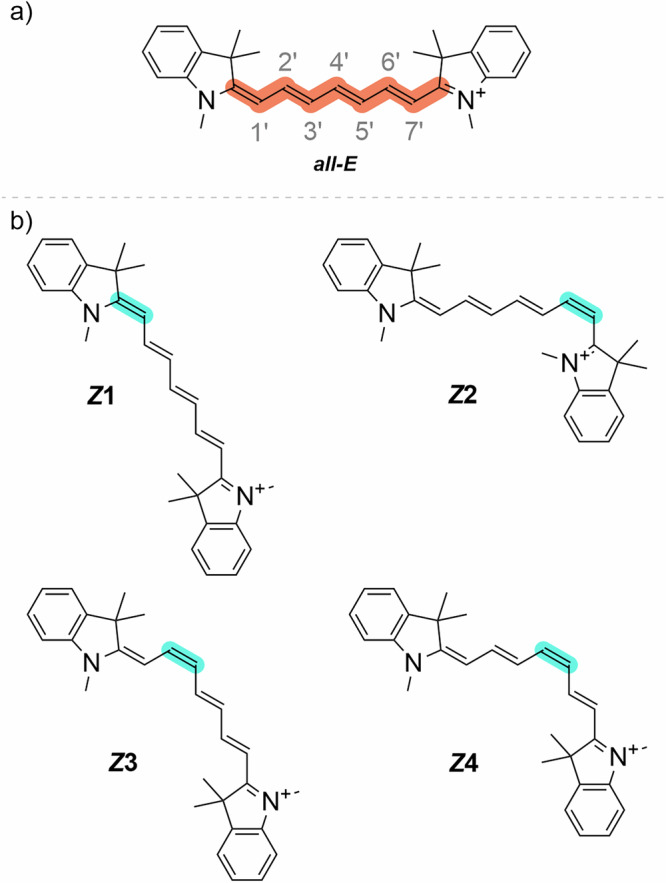
Structures of Cy7 and its photoisomers. Structures of (**a**) ***all-E*** **Cy7** and (**b**) four possible mono *Z*-photoisomers ***Z*1–*Z*4**.

We investigated prototypical heptamethine cyanine (**Cy7**, Fig. 1a) using FSR spectroscopy in acetonitrile, methanol, and water. The obtained transient Raman (TR-Raman) spectra, together with density functional theory (DFT) quantum chemical calculations, allowed us to identify all the main species formed upon **Cy7** excitation in the aforementioned solvents and their time evolution. We monitored the formation of a triplet state, which was distinguishable from the photoisomers based on Raman shifts. For the first time, we identified the formation of two of the four possible mono *Z*-isomers (Fig. 1b). Additionally, we identified an ultrafast electron transfer process that leads to the formation of cyanine radical dication (**Cy7**^•+^) and superoxide radical anion (O_2_^•–^). Although the formation of O_2_^•–^ upon **Cy7** excitation was reported earlier^49,50^, we established an unprecedented timescale for the process. Finally, we addressed the ultrafast, solvent-mediated relaxation pathway of the excited species. Quantitative interpretation of TR-Raman spectra of the singlet state, the vibrationally excited singlet state, the cyanine radical dication, and their solvated species indicates that the predominant relaxation pathways of these species are related to different C–H vibrational modes.

## Results and discussion

### Ground-state stimulated Raman spectroscopy

**Fig. 2:**
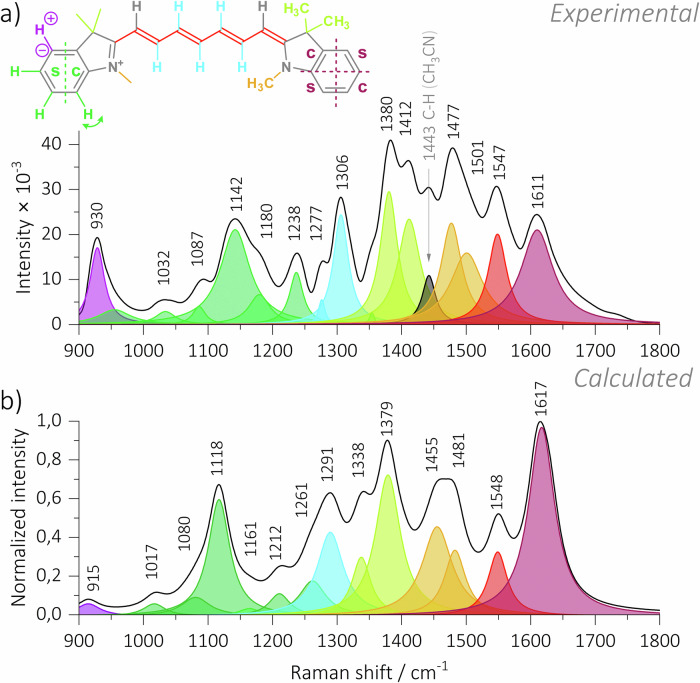
Experimental and calculated Raman spectra of Cy7. **a** The stimulated ground-state Raman spectrum of **Cy7** obtained in acetonitrile and (**b**) calculated Raman spectrum of ***all-E*** **Cy7**.

The ground-state Raman (GSR) spectra of **Cy7** in acetonitrile, methanol, and water were recorded using stimulated Raman excitation with wavelength-modulated, narrow-band pulses with picosecond duration set at 800 nm^51^. The GSR of **Cy7** in acetonitrile is shown in Fig. 2a together with the calculated Raman spectrum of ***all*-*E*** **Cy7** (Fig. 2b). Details on the computational methods can be found in the Supporting Information. We limit our discussion of the **Cy7** Raman spectrum to the spectral regions critical for elucidating photoisomerization and triplet formation. The high-intensity vibrational band observed at 1547 cm^−1^ in the experimental spectrum is attributed to the ${\nu }_{s}$ (C=C) vibration of the *E* configuration. This assignment is supported by quantum chemical calculations that predict a band at 1548 cm^−1^. The associated polyene chain C–H in-plane bending (δ_(i.p.)_) vibrational band, located at 1306 cm^−1^ in the experimental spectrum, is in good agreement with the calculated spectrum (Fig. 2b). The 1306 cm⁻¹ band assignment was further substantiated by recording the GSR of the di- (**Cy7**-2’,6’-*d*₂) and pentadeuterated (**Cy7**-2’,3’,4’,5’,6’- *d*_5_) **Cy7** derivatives. As the C–H *δ*_(i.p.)_ band was replaced by a lower-frequency C–D *δ*_(i.p.)_ vibrational band, the intensity of the 1306 cm⁻¹ band decreased noticeably in **Cy7**-2’,6’-*d*_2_ (see Figure S3), whereas this band was completely absent in **Cy7**-2’,3’,4’,5’,6’-*d*_5_ (see Figure S4). Furthermore, upon substituting hydrogen with deuterium in the chain, an apparent decline in the intensity of multiple vibrational bands within the 1000–1350 cm⁻¹ region was evident. The presence of these vibrational bands in the parent **Cy7** indicates the formation of a coupled oscillator involving interaction between the *δ*_(i.p.)_ (C–H) and the ${\nu }_{s}$ (C=C) vibrational modes. A minimal change in the GSR was detected when the solvent was changed from acetonitrile to methanol or water. The observed variations are primarily in the relative band intensities, with minimal shifts in band positions (Figures S7–S8).

### Singlet and triplet excited states and photoisomerization

The formation of both singlet and triplet states, as well as the subsequent photoisomerization of cyanine dyes, has been the focus of extensive research by TA over several decades^29,30,52,53^. A significant limitation of this approach is the substantial spectral overlap between the photoisomers and the triplet state signals. Accordingly, it is practically impossible to unambiguously determine from the TA data which photoisomer is formed^30^. This prompted further investigation into the photoisomerization of **Cy7** with FSR spectroscopy. The experimental setup utilized for measurements on the fs to sub-ms time scale was identical to that previously described^44,54^. FSR spectroscopy is a three-pulse technique. In the present setup, the pump pulse wavelength was set to 735 nm (the **Cy7** absorbance maximum), the Raman pulse was fixed at 800 nm, and the white-light supercontinuum was used as the probe.

We initially performed femtosecond broadband transient absorption spectroscopy (fs-TA) in methanol. The excitation wavelength was set to 735 nm, and the instrument response function (IRF) was approximately 75 fs full width at half-maximum (FWHM; the experimental details of the fs-TA setup and data processing are given in the Supporting Information). The ground-state bleach (GSB) and excited-state absorption (ESA) occurred at approximately 755 and ≈505 nm, respectively. A global analysis was performed, employing a previously described homemade software program^55,56^, under the assumption of the sequential kinetic model. To achieve a satisfactory fit, a total of four species providing four evolution-associated difference spectra (EADS) were required. The analysis of fs-TA data obtained from aerated methanol revealed the presence of two distinct species: **EADS1** and **EADS2** are the representations of the ultrafast relaxation of excited **Cy7** (*n* > 0 → *n* = 0 of S_1_), with time constants of 1.01 and 3.70 ps, respectively. The third species, **EADS3**, was attributed to the singlet state (S_1_, *τ* = 0.79 ns), and the fourth species (**EADS4,** *τ* = 22.6 ± 3.1 μs) was attributed to photoisomers (Figure S9). The results of the experiment demonstrated that when fs-TA was recorded in degassed methanol, the lifetime of **EADS4** increased (350 ± 10.2 μs), indicating that the signal also includes contributions from the triplet state. It is important to note that the **EADS4** absorbance at approximately 800 nm overlaps with the Raman pulse used in our FSR spectroscopy setup, resulting in pre-resonance Raman conditions. These conditions offered an exceptionally high-intensity Raman signal and a high signal-to-noise ratio for FSR spectra. The lifetimes of **EADS1,** **EADS2**, and **EADS3**, when recorded under degassed conditions, were 1.33 ps, 22 ps, and 0.82 ns, respectively. Our results are in good agreement with the previously published values^57^.

**Fig. 3:**
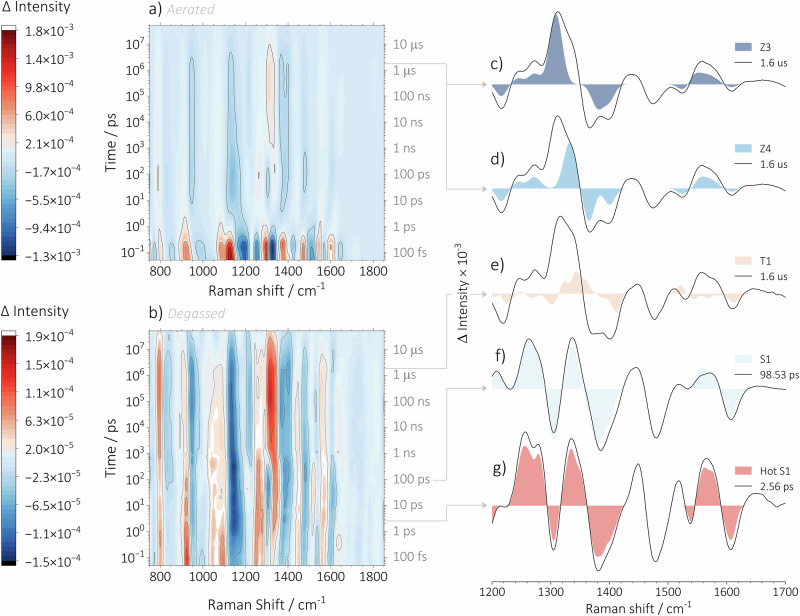
Experimental FSR spectra of Cy7 in aerated and degassed acetonitrile. Experimental FSR spectra presented as a contour graph recorded in (**a**) aerated and (**b**) degassed acetonitrile. The extracted TR-Raman spectra of (**c**) ***Z*3 Cy7**, (**d**) ***Z*4 Cy7**, (**e**) T_1_ **Cy7**, (**f**) S_1_ **Cy7**, and (**b**) hot S_1_ states of **Cy7**. Experimental FSR spectra in (**c**–**g**) are shown as black lines, and the extracted TR-Raman spectra of the corresponding species are represented by colored-filled curves.

We first examined our FSR spectroscopy data qualitatively (Fig. 3). Several distinguishable regions were observed in the FSR spectra of **Cy7** in aerated (Fig. 3a) and degassed (Fig. 3b) acetonitrile. Acetonitrile was selected as the solvent of choice because **Cy7** exhibits the higher photostability in this solvent, than in water or methanol. The decreased photostability of **Cy7** dyes in aqueous solutions is relevant for imaging applications and is well established in the literature^9,15^. Initially, we did not consider a timescale shorter than ≈1 ps; therefore, we will first discuss the ps–μs region. The observed signals suggested dynamics involving the formation and subsequent decay of several excited species, including singlet (S_1_) and triplet (T_1_) states and **Cy7** photoisomers. Most of the spectral dynamics were limited to the ps–ns range, while after ≈ 10 ns, the system evolved solely through the decay of the formed species. Global analyses performed using previously described homemade software^55,56^ and Glotaran^58^ did not yield meaningful results. Complex kinetics involving a combination of sequential and parallel processes led to a virtually infinite number of convergent points. To circumvent this issue, we took a different approach. A target analysis model was built based on physically reasonable assumptions that best describe the experimental data (see Figure S53).

First, we want to address the photoisomerization of **Cy7**. For this, two regions of interest were examined: the region of C=C *ν*_s_ vibrations (1500–1650 cm^–1^) and the accompanying C–H *δ*_(i.p.)_ region (1200–1420 cm^–1^). Other spectral regions that yielded less informative data about the C = C bond configuration were excluded from the analysis. Moreover, the significant absorbance of EADS4 at 800 nm leads to pre-resonance Raman conditions with an 800-nm Raman pulse. This explains the exceptionally high signal intensity and high signal-to-noise ratio observed in FSR spectra. This allowed us to analyze all individual FSR spectra from 1.14 ps to 51.2 μs by fitting them with Gaussian peaks within the selected frequency range, which gave us an overview of peak position and intensity changes over time (Figures S37–S40).

**Fig. 4:**
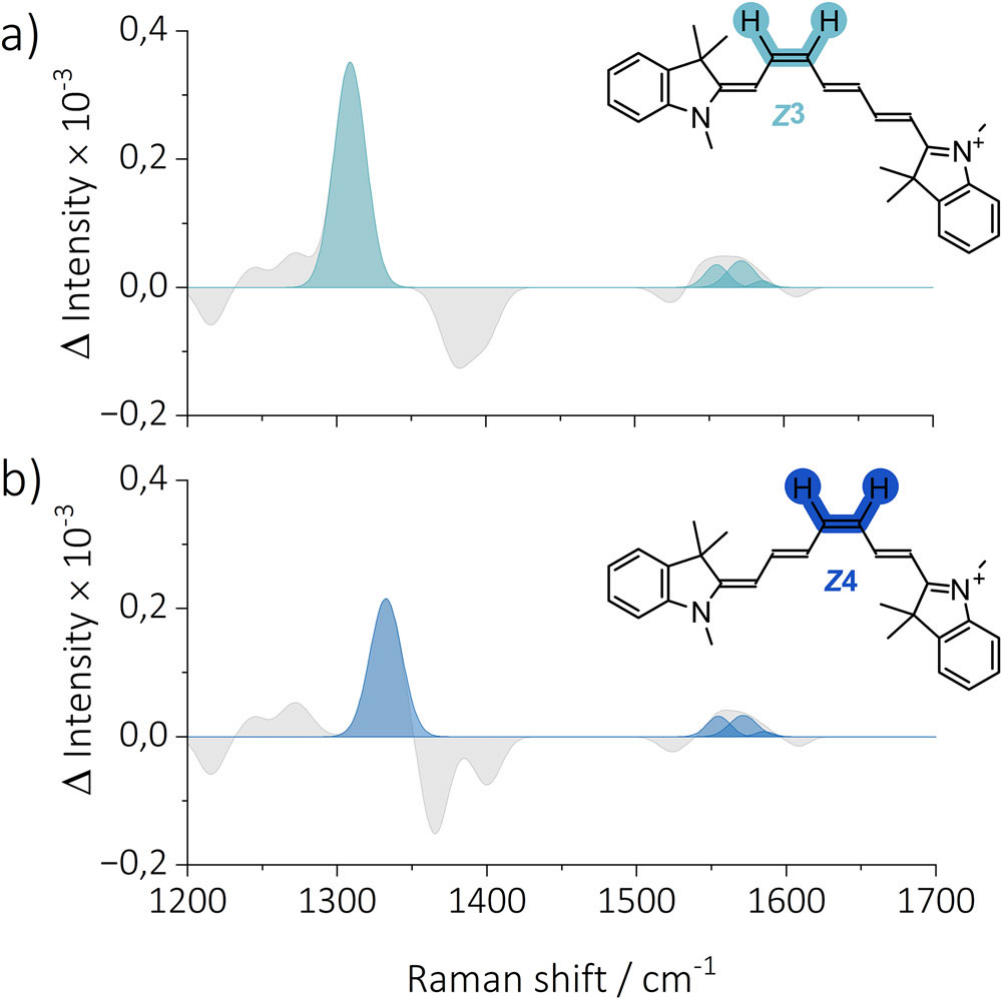
TR-Raman spectra of the observed Cy7 photoisomers. The extracted TR-Raman spectra of (**a**) ***Z*3** and (**b**) ***Z*4** with highlighted vibrational bands for ${\delta }_{i.p.}$ (C–H) in combination with ${\nu }_{s}$ (C=C) in the *Z* configuration. Negative vibrational bands were not fitted as they represent the GSB of the parent ground state **Cy7**.

**Fig. 5:**
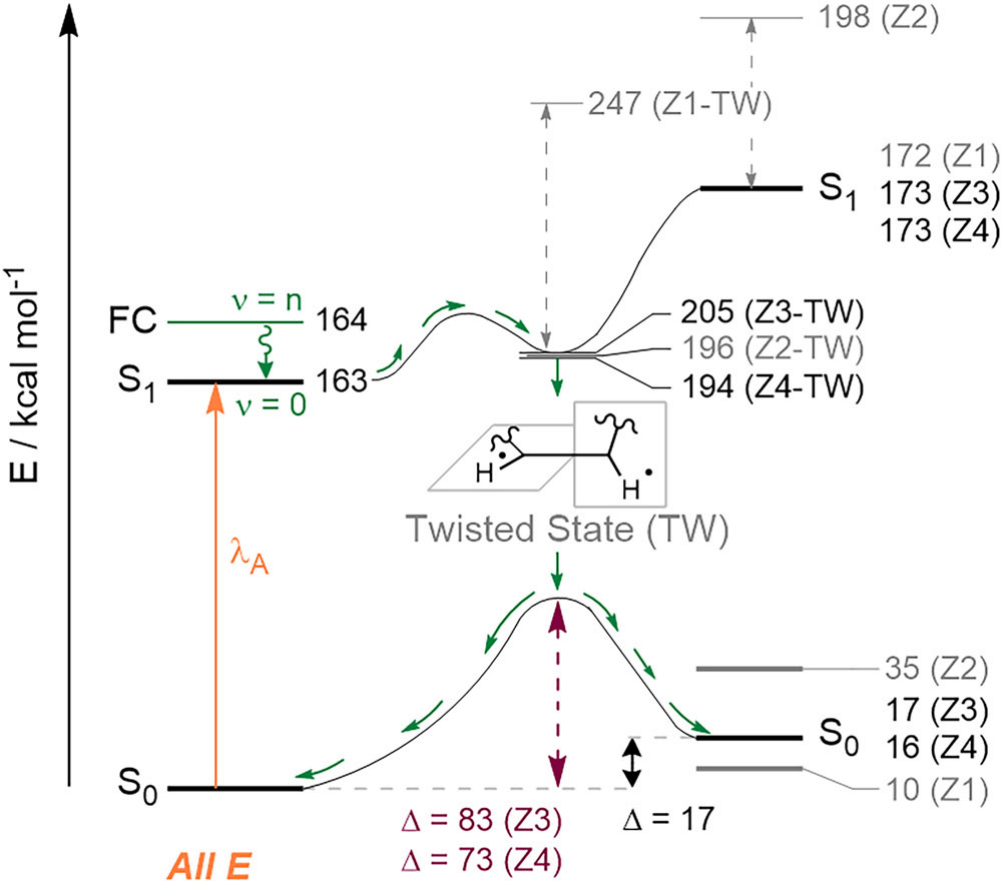
Calculated Photoisomerization of Cy7. A schematic potential energy surface obtained by CIS(D)/TZVP calculations. The calculated energies are shown next to their corresponding states.

The construction of TR-Raman spectra of photoisomers and T_1_ was carried out in the following manner: Given the rapid quenching of T_1_ by oxygen in aerated acetonitrile, it was hypothesized that the signals above 1 μs originate from oxygen-insensitive species. This would allow for the discrimination of TR-Raman spectra of T_1_ and photoisomers. Therefore, we assigned FSR signals recorded in aerated acetonitrile at 1.6 μs to photoisomers. A thorough examination of the experimental data, in conjunction with a comparison of the calculated Raman spectra of each of the photoisomers (see Figures S16-S23), let us assign the experimental TR-Raman spectrum of the mixture of ***Z*3** and ***Z*4 Cy7** (Fig. 3c-d and S41). The TR-Raman spectra for both ***Z*3** and ***Z*4** show ground state bleaching (**GSB**) of ${\nu }_{s}$ (C=C) with the *E* configuration. The most intense newly formed band is ${\delta }_{i.p.}$ (C–H) of the *Z* configuration at 1309 cm^–1^ for ***Z*3** (Fig. 4a) and 1332 cm^–1^ for ***Z*4** (Fig. 4b), attributed by quantum chemical calculations and calculated energetic landscape for the photoisomerization of **Cy7** (Figure S29-S36). The calculations demonstrated that the energy profiles for the formation ***Z*2,** ***Z*3**, and ***Z*4** were equivalent. Conversely, a substantial energy barrier was identified for the photoisomerization process, yielding ***Z*1** (twisted state). Assuming that the Raman cross sections are similar for all four photoisomers, the absence of ***Z*1** and ***Z*2** is likely due to an energetic barrier preventing their formation. The schematic potential energy surface, derived from the calculated results, is depicted in Fig. 5. In contrast to the fs-TA, where the large spectral overlap of the broad absorption bands of the **Cy7** photoisomers hinders identification, the significantly different vibrational patterns of the **Cy7** photoisomers allowed us to distinguish and identify the formed photoisomers using FSR spectroscopy.

**Fig. 6:**
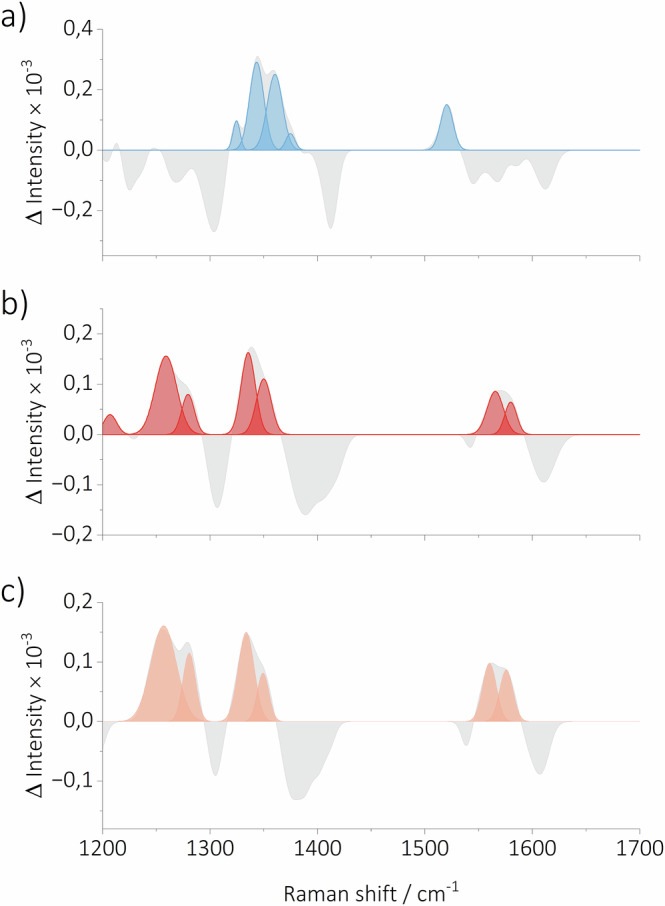
TR-Raman spectra of the hot S_1_, S_1_ and T_1_ states. The TR-Raman spectra of (**a**) T_1_, (**b**) S_1_, and (**c**) hot S_1_ states of **Cy7** extracted from the FSR spectroscopy data recorded in acetonitrile. Negative vibrational bands were not fitted as they represent the GSB of the parent ground state **Cy7**.

Analogous to the photoisomers, the TR-Raman spectrum of the T_1_ state was obtained based on the assumption that the experimental signal in degassed acetonitrile contains contributions from both T_1_ and photoisomers. The triplet state signal for degassed samples represents only a small component of the FSR spectra because the ratio of the quantum yields of ISC (*Φ*_ISC_ = 0.0089^59^) and photoisomerization (*Φ*_E→Z_ = 0.09^60^) is small. Subsequently, the experimental TR-Raman spectrum recorded under aerated conditions was subtracted from the spectrum recorded under degassed conditions at 1.6 μs. It yielded the TR-Raman spectrum of T_1_ (Fig. 3e), which features GSB of ${\nu }_{s}$ (C=C) combined with the formation of a new band ≈1590 cm^–1^ at the same time, resulting in an overall complex pattern (Fig. 6a). The measured Raman shift of ≈1590 cm^–1^ is well supported by a computed signal at 1575 cm^–1^ associated with the$\,{\nu }_{s}$ (C=C) vibration (Figure S43).

After the TR-Raman spectra of ***Z4***, ***Z3***, and T_1_ in acetonitrile were obtained, the focus was directed towards the S_1_ state. Assuming that both S_1_ and T_1_ contribute to the FSR spectra under degassed conditions in acetonitrile, the TR-Raman spectrum of T_1_ was subtracted from the experimental FSR spectrum at 98.53 ps to obtain the TR-Raman spectrum of S_1_ (Fig. 3f). The spectrum displayed an intense vibrational band at 1337 cm^–1^ attributed to ${\delta }_{i.p.}$ (C–H) vibrations, and two overlapping vibrational bands at 1558 and 1575 cm^–1^, corresponding to stretching vibrations of a polyene chain ${\nu }_{s}$ (C=C) and an aromatic ring, respectively. The GSB of ${\delta }_{i.p.}$ (C–H) and ${\nu }_{s}$ (C=C) of all-*E* ground state **Cy7** are observed in addition to newly formed bands (Fig. 6b). The obtained TR-Raman spectrum of S_1_ exhibited a strong resemblance to the calculated spectrum (Figure S44). In order to ascertain the reliability of the previously constructed S_1_ spectrum, a comparison was made between the spectrum and experimental data. It was determined that the S_1_ spectrum was not an accurate representation of the experimental data at times shorter than ≈3 ps. In consideration of the lifetimes of **EADS2** derived from the fs-TA data, it was hypothesized that the spectrum of an additional species, probably a vibrationally excited S_1_ state (*ν* > 0, designated as hot S_1_), must precede the S_1_ state. Subsequently, a spectrum of the hot S_1_ state was constructed (Fig. 3g) by subtracting the spectrum of S_1_ from the experimental FSR spectrum recorded under degassed conditions at 2.56 ps. The TR-Raman spectrum of hot S_1_ state closely resembles that of S_1_, featuring GSB of (C=C) ${\nu }_{s}$ and (C–H) ${\delta }_{i.p.}$ of **Cy7** with the all *E* configuration. The new vibrational bands at 1559 and 1577 cm^–1^ are assigned to the ${\nu }_{s}$ (C=C) and aromatic ring stretching vibrations, respectively. In contrast, the bands in the 1215–1367 cm^–1^ range are ascribed to the new ${\delta }_{i.p.}$ (C–H) and (C–H) vibrations of aromatic rings (Fig. 6c). In all cases, FWHM of the vibrational bands in the TR-Raman spectrum of the hot S_1_ state is greater than that of the S_1_ state, which further supported our assignment.

### Electron transfer between excited Cy7 and ground-state oxygen: formation of superoxide

With the resolved TR-Raman spectra of **Z*4***, **Z*3***, and T_1_, S_1_, and hot S_1_ states in hand, a target analysis^61^ was performed to extract kinetic traces of all species, using a custom-designed routine (Supporting Information). A series of kinetic models were examined, yet the analysis of residuals indicated a systematic deviation of the fitted data from the experimental FSR data in aerated acetonitrile. This finding suggested the presence of an additional, previously undetected species. To address this, we considered the possibility of electron transfer (ET). Photoinduced electron transfer (PET) processes have been well established for cyanine dyes. For instance, PET is a key process in photography, where a cyanine dye is used to photoreduce silver ions^3^. Numerous reports have evidenced the participation of both singlet and triplet excited states of cyanine dyes in PET^62–65^. The recent revival of interest in photoinitiators has led to the utilization of cyanine dyes as NIR redox photoinitiators^66,67^.

**Fig. 7:**
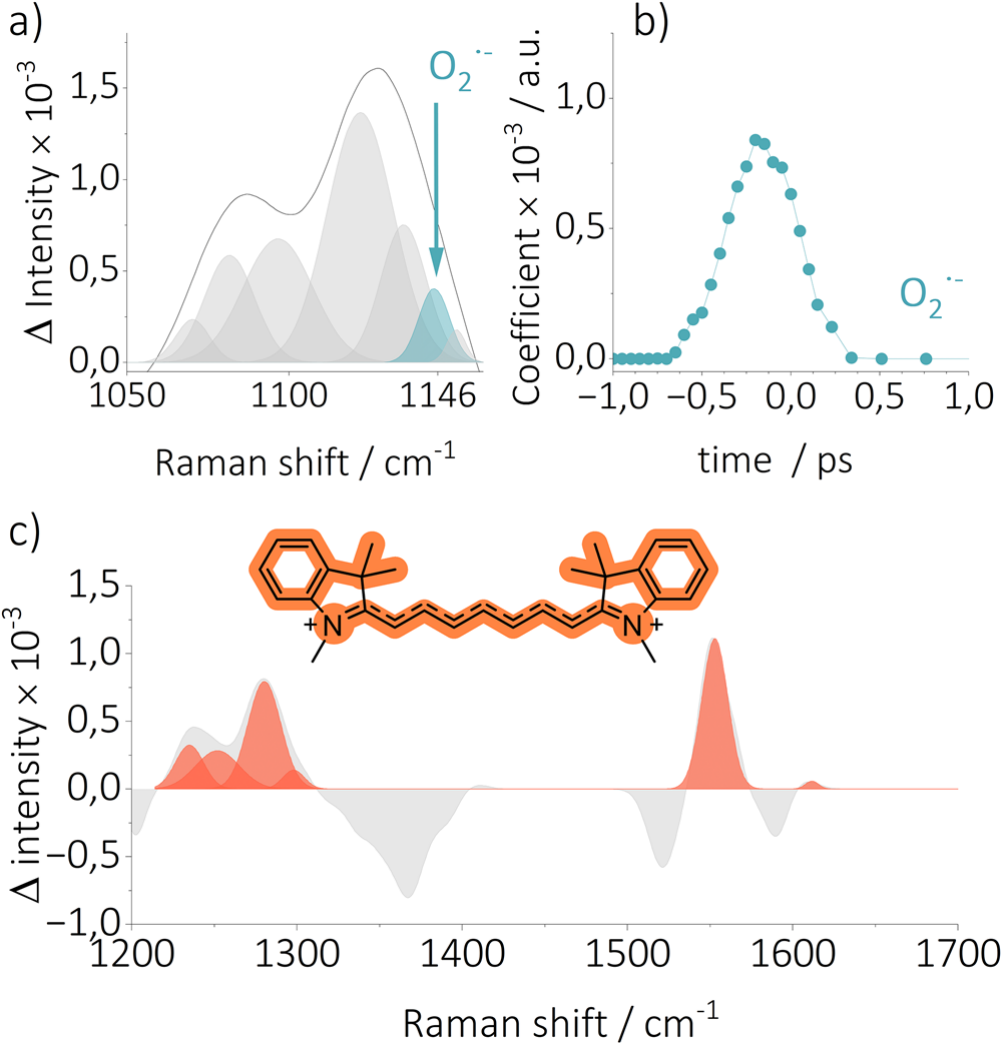
The FSR spectrum of Cy7 in aerated acetonitrile at *t* = 0. **a** Enlarged view of the part where the O_2_^•^⁻ band is observed, and (**b**) its temporal evolution. **c** A TR-Raman spectrum of **Cy7**^•+^ extracted from experimental FSR spectrum recorded in aerated acetonitrile. Negative vibrational bands were not fitted as they represent the GSB of the parent ground state **Cy7**.

The only other species present in our system was molecular oxygen, leading us to hypothesize that ET could occur from excited cyanine to oxygen. The feasibility of electron transfer between the excited S_1_ state of **Cy7** and oxygen was estimated by calculating the free energy change$\,{\Delta G}_{{PET}}$ of –6.92 kcal mol^–1^ (see the Supporting Information). The photosensitized production of O_2_^•^⁻ by a series of cyanine dyes has been indirectly evidenced by electron paramagnetic resonance (EPR) spectroscopy using spin trapping^68^, as the lifetime of O_2_^•^⁻ at room temperature is ≈1 μs^69^, preventing its direct detection by EPR. A subsequent examination of the TR-Raman spectra at ultrashort times (up to ≈500 fs) revealed a band at 1144 cm^–1^ (Fig. 7a). This band was assigned to the O–O stretching vibration (${\nu }_{s}$) and exhibits strong agreement with the established literature value^70^. The experimental Raman spectrum of O_2_^•–^ in water is distinguished by a band at 1147 cm^–1^, originating from the O–O ${\nu }_{s}$ and a weak overtone band at 2266 cm^–1^^70^_._In the same work, the gas phase O–O ${\nu }_{s}$ frequency of O_2_^•–^ was estimated to be 1105 ± 4 cm^–1^, shifted by 57 ± 20 cm^–1^ to higher frequencies due to solvation effects. Our calculations with the CCSD(T)/aug-cc-pVQZ approach predict the Raman band of O_2_^•^⁻ at 1085 cm^–1^ in the gas phase and 1114 cm^–1^ in acetonitrile, resulting in the shift of 29 cm^–1^ to higher frequencies, attributed to solvation effects. The overtone band was found to be outside the detection window of our FSR spectroscopy setup, thus it was not detected. Furthermore, the 1144 cm^–1^ band was not detected in the FSR spectra recorded under degassed conditions (Figures S46–S49), unambiguously supporting its assignment.

The O_2_^•^⁻ band was only observable at ultrashort times (up to ≈200 fs; the entire solvent envelope is impulsively excited^35^, Fig. 7b). The detection of the O_2_^•^⁻ band is understandable because the electron transfer process is faster than the time resolution of our FSR spectroscopy setup. As soon as the excitation (50 fs) and probe (75 fs, at given wavelength, 2 ps for the entire envelope) pulses begin to overlap in time (at –185 fs delay time), the O_2_^•^⁻ band is observed. However, this results in the observed FSR signal being the sum of signals from the excited **Cy7** species, the surrounding solvent molecules, and in this case, probably solvated O_2_^•^⁻^33^. Electron transfer faster than the rate of diffusion might indicate the formation of the ground-state O_2_–**Cy7** complex. To test this hypothesis, the absorption spectrum of **Cy7** in acetonitrile was measured in both the presence and absence of O_2_. The resulting difference in the absorption spectra was only manifested in a broadening of the long-wavelength absorption band of **Cy7** (Figure S11). This finding is consistent with several studies of ground-state complexes of organic molecules with O_2_ reporting specific broad bands in the absorption spectra at longer wavelengths^71,72^. Our quantum chemical calculations suggest the formation of a ground-state O_2_–**Cy7** complex with $\Delta {G}_{{complex}}$ of 0.18 kcal mol^–1^. As an alternative, the formation of **Cy7**^•^⁺ and O₂^•^⁻ via electron transfer may originate from the collision complex. However, the ultrafast nature of the process strongly suggests that it stems from the ground-state O₂-**Cy7** complex. To obtain the TR-Raman spectrum of **Cy7**^•+^, it was hypothesized that the FSR spectrum obtained in degassed acetonitrile at 2.56 ps consists exclusively of a hot S_1_ state signal, while the FSR spectrum recorded in aerated acetonitrile contains the **Cy7**^•+^ signal in conjunction with that of hot S_1_. Consequently, the spectrum of **Cy7**^•+^ (Fig. 7c) was obtained by subtracting the former FSR from the latter.

In order to provide further support for this analysis, an attempt was made to measure the TR-Raman spectrum of **Cy7**^•+^ independently. We designed an experiment in which excited 9,10-dicyanoanthracene (DCA) was used to oxidize ground state **Cy7** in acetonitrile, thereby producing **Cy7**^•+^ and 9,10-dicyanoanthracene radical anion (DCA^•–^; Supporting Information). The calculated ${\Delta G}_{{PET}}$ of –64.57 kcal mol^–1^ indicated that the transfer should be highly exergonic (Supporting Information). Some other heptamethine cyanine radical dication derivatives were shown to be produced electrochemically and were characterized by UV-Vis^73^ and EPR spectroscopies^74^. A distinguishing characteristic of these species is a broad weak absorption band centered around 800 nm^75^. Unfortunately, the absorption spectrum of **Cy7**^•+^ was not reported due to its very short lifetime and rapid dimer formation^76^. Assuming that **Cy7**^•+^ has similar absorption properties to those of other reported heptamethine cyanine radical dications, significant Raman signal enhancement was expected due to the resonance conditions of the 800-nm Raman pulse with the weak absorption band of **Cy7**^•+^ at ≈800 nm^70^. In contrast, the intensity of the Raman signals of DCA^•–^ was expected to be much lower, as DCA^•–^ exhibits no significant absorption at 800 nm^77^. The TR-Raman spectrum of **Cy7**^•+^ obtained in this study closely resembles the previously obtained subtraction spectrum and is further supported by the quantum chemical calculations (Figures S51, S52). Thus, based on the TR-Raman and quantum chemical calculations, **Cy7**^•+^ was determined to be in its electronically ground state, not in its electronically excited state.

After confirming the presence of O_2_^•–^ and **Cy7**^•+^, several target analysis models were tested that consider all the transients described so far (Figure S54). The model that best described the experimental FSR data recorded in both aerated (Figure S57) and degassed acetonitrile (Figure S58) at times above 500 fs consisted of **Cy7**^•+^ and the hot S_1_ state formed simultaneously. The hot S_1_ further decayed to the S_1_ state, which was later converted to the T_1_, ***Z*3**, and ***Z*4** species. We used the same model to fit the experimental FSR spectra in aerated methanol (Figure S59) and water (Figure S60), achieving satisfactory results for times above 1 and 2 ps, respectively. In addition, we fitted the model output to calculate the approximate lifetimes of the identified species. It should be noted that FSR spectroscopy cannot provide exact transient lifetimes^34,35^. As shown in Table 1, the results indicated that the hot S_1_ state decays to S_1_ within 3 ps in all three solvents, regardless of the solvent properties or the presence of O_2_. The obtained S_1_ lifetimes are in good agreement with the previous literature reports and the values provided by our fs-TA measurements. As expected, the T_1_ decay in all three solvents under aerated conditions was fast, on the order of several ns, while the lifetime increased to less than 13 μs in degassed acetonitrile. The lifetimes of ***Z*3** and ***Z*4**, which were on the order of tens of μs, were found to be independent of the solvent or the presence of O_2_ and similar to those obtained by fs-TA measurements (Table 1). Finally, the observed lifetimes of **Cy7**^•+^ decreased from 276 to 10 ps when acetonitrile was changed to water. This behavior is expected for electron-deficient species such as **Cy7**^•+^, which can be attacked by nucleophilic solvents such as methanol or water. This significantly decreases their lifetime in these solvents. Additionally, the high concentration of **Cy7** required for FSR spectroscopy increases the dimer formation efficiency, providing additional pathway for decreasing its apparent lifetime^78,79^. The very short **Cy7**^•+^ lifetimes obtained by FSR spectroscopy also explain why it cannot be characterized by EPR (CW-EPR, X-band; lowest time limit 1 μs)^74^.

**Table 1:** The lifetimes of various species as obtained from FSR spectroscopy and fs-TA data

Transient species	Solvent / Conditions ^*a*^	FSR spectroscopy lifetimes	fs-TA lifetimes ^*b*^
Hot S_1_	CH_3_CN / O_2_ CH_3_CN / deg MeOH / O_2_ H_2_O / O_2_	2.1 ± 0.4 ps 1.9 ± 0.5 ps 2.5 ± 0.6 ps 1.8 ± 0.5 ps	n.d^c^. n.d. 3.7 ns 5.1 ns
S_1_	CH_3_CN / O_2_ CH_3_CN / deg MeOH / O_2_ H_2_O / O_2_	1.2 ± 0.4 ns 1.6 ± 0.3 ns 0.65 ± 0.05 ns 0.39 ± 0.04 ns	n.d. (1.37 ns)^29^ n.d. 0.78 ns (0.79 ns)^57^ 0.35 ns
T_1_^c^	CH_3_CN / O_2_ CH_3_CN / deg MeOH / O_2_ H_2_O / O_2_	4.2 ± 0.8 ns 12.8 ± 2.7 μs 1.9 ± 0.12 ns 0.65 ± 0.05 ns	n.d. n.d. n.d. n.d.
*Z*-photoisomers	CH_3_CN / O_2_ CH_3_CN / deg MeOH / O_2_ H_2_O / O_2_	***Z*3**: 17 ± 3 μs, ***Z*4**: 16 ± 3 μs ***Z*3**: 16 ± 2 μs, ***Z*4**: 20 ± 4 μs ***Z*3**: 16 ± 2 μs, ***Z*4**: 59 ± 8 μs ***Z*3**: 11 ± 3 μs, ***Z*4**: 31 ± 3 μs	n.d. n.d. 22.6 ± 3.5 μs ^d^ 12.8 ± 2.5 μs ^d^
**Cy7**^•+^	CH_3_CN / O_2_ CH_3_CN / deg MeOH / O_2_ H_2_O / O_2_	276 ± 50 ps n.d. 27.0 ± 3.2 ps 9.8 ± 5.2 ps	n.d. n.d. n.d. n.d.

^a^Obtained at 22.5 ± 0.5 °C; the **Cy7** concentrations were adjusted for *A* = 1 ± 0.05 (735 nm; 1-mm optical pathlength) in aerated (O_2_) or degassed (deg) solvents.

^*b*^The lifetimes obtained from our fs-TA measurements, together with the literature values in parentheses.

^*c*^Not determined.

^*d*^It was assumed that the contribution to the evolution-associated difference spectra under aerated conditions comes only from the photoisomers, so this value represents the average value for both photoisomers.

### Dynamics on a sub-500 fs timescale

**Fig. 8:**
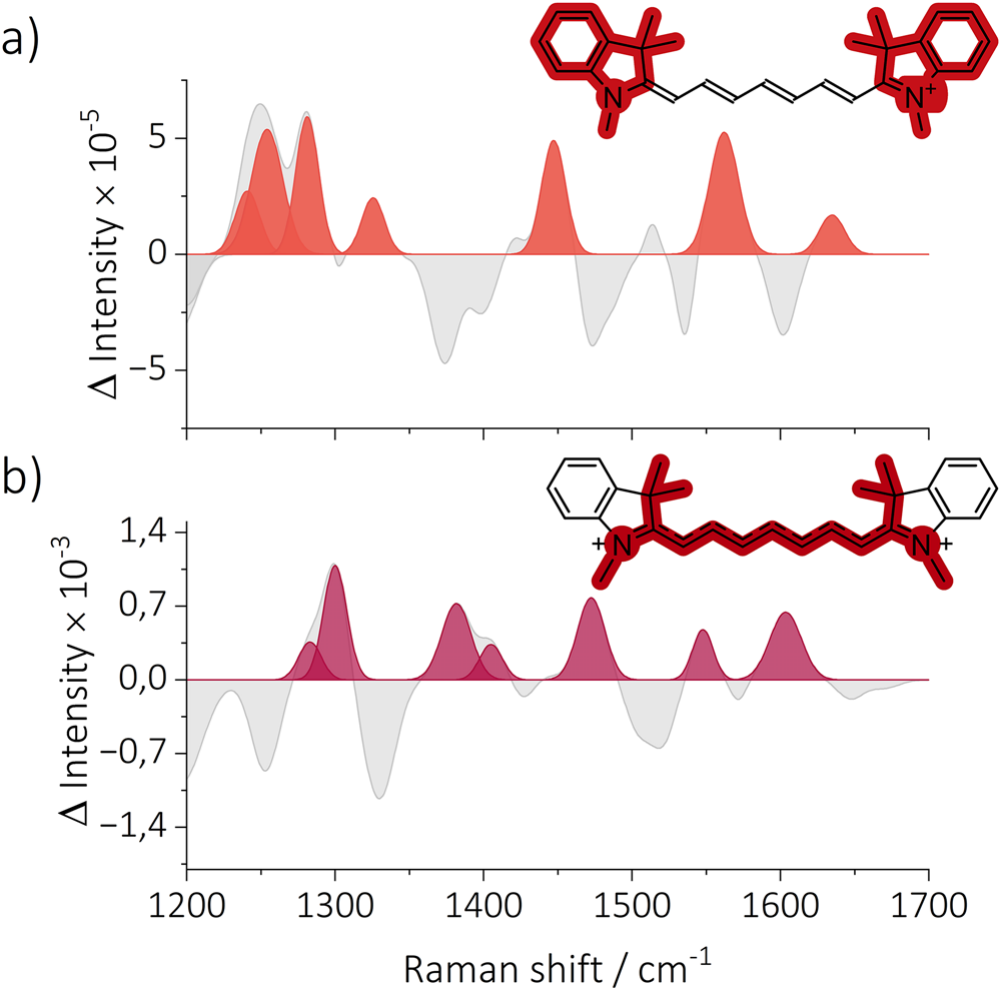
TR-Raman spectra of the solvated hot S_1_ and solvated Cy7^•+^ species. The TR-Raman spectra extracted from the FSR data recorded in aerated acetonitrile, of (**a**) the solvated hot S_1_ state and (**b**) the solvated **Cy7**^•+^ species in the 1200–1700 cm^–1^ region, used for the second set of target analysis models. Negative vibrational bands were not fitted as they represent the GSB of the parent ground state **Cy7**.

**Fig. 9:**
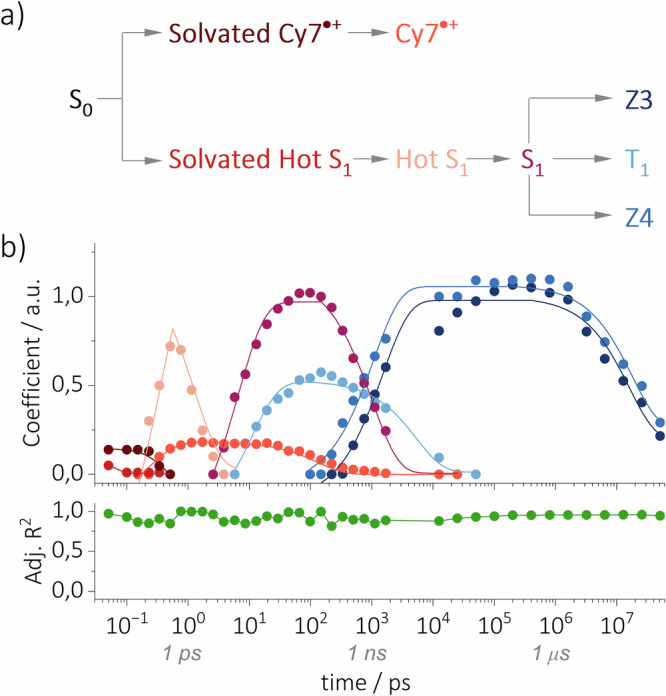
First microseconds of the life of excited Cy7. **a** Reaction mechanism diagram showing the transient species formed upon excitation of **Cy7** and (**b**) their time evolution in aerated acetonitrile. The target analysis model for aerated acetonitrile fits the experimental data for the entire time range due to the inclusion of solvated hot S_1_ and **Cy7**^•+^ species. The *R*^2^ values for the model fit to the FSR spectroscopy data are shown.

Next, we examined the dynamics of **Cy7** in aerated acetonitrile at ultrashort times ( < 500 fs), where the previous target analysis model failed to produce a satisfactory fit. We hypothesized that the TR-Raman spectra under degassed conditions corresponded to the solvated hot S_1_ state (Fig. 8a), and that under aerated conditions, the spectra originated from the solvated hot S_1_ state and solvated **Cy7**^•+^. Therefore, the difference in TR-Raman spectra at *t* = 0 fs recorded under aerated and degassed conditions provided TR-Raman spectra of solvated **Cy7**^•+^ (Fig. 8b). A detailed analysis of the solvated hot S_1_ state and **Cy7**^•+^ would require calculating the Raman spectra of the respective species interacting with several solvent molecules. Here, we present only a qualitative interpretation. The newly formed bands are tentatively assigned to the aromatic ring quadrant stretching (1558 cm^–1^), CH_3_ bending coupled with ${\delta }_{i.p.}$ (C–H, of both solvated hot S_1_ and acetonitrile species at 1325 and 1447 cm^–1^, respectively), and the combination of ${\delta }_{i.p.}$ (C–H) and (C–H) aromatic ring vibrational bands (1281–1240 cm^–1^, Fig. 8a). These vibrations are likely responsible for the energy dissipation of the solvated hot S_1_ state. On the other hand, the most intense newly formed vibrational bands – polyene chain stretching ${\nu }_{s}$ (C=C; 1547 cm^–1^), quadrant stretching (1603 cm^–1^), (C–H) bending vibrations of CH_3_ (**Cy7**^•+^ 1473, 1405, and 1383 cm^–1^), and ${\delta }_{i.p.}$ (C–H; 1301 cm^–1^) – are most probably responsible for the energy dissipation in the TR-Raman spectrum of solvated **Cy7**^•+^ (Fig. 8b). A second set of target analysis models was subsequently constructed that considered the TR-Raman spectra of the solvated hot S_1_ and **Cy7**^•+^ states (Figure S55). The model that best describes the experimental FSR spectroscopy data, along with the temporal distribution of species obtained by fitting the experimental data, is shown in Fig. 9a. The difference between the new model and the previous one (Figure S54) is in the inclusion of solvated species. These species were observable only at ultrashort time scales, and their inclusion improved the fit at times shorter than 500 fs for FSR spectra recorded in aerated acetonitrile. Both solvated species decayed rapidly ( ≈ 500 fs) to their unsolvated counterparts. This is expected because the entire solvating shell around the species becomes impulsively excited on this time scale. Thus, the resulting TR-Raman spectra reveal the **Cy7** species and the surrounding solvent molecules (i.e., solvated species).

The high intensity of the TR-Raman signals combined with FSR spectrum recording in both aerated and degassed acetonitrile, as well as target analysis, allowed us to distinguish the chemical species formed upon **Cy7** excitation. The most comprehensive overall picture of the photophysical and photochemical processes was constructed for **Cy7** in aerated acetonitrile (Fig. 9), in which the transient species showed the strongest Raman signal intensity. Upon excitation of **Cy7**, the first observed species formed were the solvated hot S_1_ state (in the absence of O_2_) and both the solvated hot S_1_ and **Cy7**^•+^ species (in the presence of O_2_). The latter transients are the result of extremely fast electron transfer and indicate the presence of a ground-state O_2_–**Cy7** complex. Such ground-state complexes are very weakly bound and exhibit partial charge transfer, which leads to complete charge transfer and radical pair formation upon excitation^69^. The effective concentration of such complexes is typically very small. Due to the significant uncertainty in determining the molar absorption coefficient of these complexes, estimates of their concentrations are subject to large errors^71^.

Subsequently, the solvated species decay into their unsolvated counterparts within ≈500 fs, i.e., at the end of the time window during which the solvated shell is impulsively excited. This is followed by the formation of **Cy7**^•+^ and the hot S_1_ state. **Cy7**^•+^ can be further converted to a dimer^78,79^, or react with a nucleophilic solvent^80,81^. The hot S_1_ state rapidly decays to the thermally equilibrated S_1_ state. The similarity between the hot S_1_ and S_1_ TR-Raman spectra indicates a large overlap between the promoting and accepting vibrational modes and is most likely responsible for the very fast hot S_1_ → S_1_ conversion^82,83^. Furthermore, the newly observed vibrational bands in the TR-Raman spectrum of S_1_ (Fig. 6c) indicate the (C–H) vibrations of the polyene chain and the breathing vibrations of the aromatic rings, which are responsible for its nonradiative relaxation. These results further support the finding that both deuteration of the chain^23^ (and/or heterocyclic end groups) and conformational restriction of the cyanine chain^7^ increase fluorescence quantum yields. In the next step, photoisomerization from the S_1_ state selectively produces the **Z3** and **Z4** photoisomers. Our results corroborate observations made by fluorescence correlation and transient state excitation modulation spectroscopies, which indicated the formation of multiple photoisomers with an unspecified chain configuration^84^. Further research is necessary to elucidate the possible subsequent interconversion of the isomers in the excited state. Finally, considering the very small geometrical changes required during ISC, the fast formation of T_1_ is understandable. However, since the Raman cross section and total concentrations of the short-lived species are unknown, it was not possible to determine the quantum yields for their formation^34^. The kinetic model derived in this study is not the only plausible interpretation; however, it provides the most rational explanation of the experimental FSR data. The precise identification of the reaction intermediates and determination of their time-resolved behavior provides a clear picture of the photochemistry of this common molecule.

## Conclusion

The exceptionally strong TR-Raman signals obtained in aerated and degassed solvents enabled us to gain insight into the photophysical and photochemical processes that occur when the prototypical heptamethine cyanine dye **Cy7** is photoexcited. For the first time, we experimentally uncovered the selective formation of two **Cy7** photoisomers, ***Z*3** and ***Z*4**, as well as another short-lived species, the triplet state. Furthermore, FSR spectroscopy enabled the detection of the electron transfer process that leads to the formation of the cyanine radical dication and the superoxide radical anion. These species form on an unprecedentedly short timescale in an aerated solvent. Identifying the relaxation pathways of the vibrationally excited singlet state, the relaxed singlet state, cyanine radical dication, and their solvated counterparts revealed that the predominant relaxation pathways of these species are related to different C–H vibrational modes of the polymethine chain and the heterocyclic end groups. Overall, our work provides an in-depth overview of the fundamental processes occurring during **Cy7** excitation that has not been reported before. This information may be essential for further developing cyanine-based fluorophores for use in imaging, drug delivery, and photodynamic therapy.

## Methods

Detailed procedures for experimental and computational work are available in the Supporting Information.

### Femtosecond transient absorption spectroscopy (fs-TA)

fs-TA measurements were conducted using homebuilt 1-kHz transient absorption and femtosecond-stimulated Raman spectroscopy setups constructed around femtosecond Ti:sapphire amplifiers Femtopower (Spectra Physics) and Solstice amplifier (Spectra Physics), which shared a common oscillator. The amplifiers were synchronized by electronic triggering and an optical delay of the seed prior to amplification, allowing setting the delay between their pulses up to <1 ms with fs precision. Two laser beams, a pump and a probe, were used in TA experiments. White-light supercontinuum generated in an argon-filled hollow core fiber (Ultrafast Innovations, Savannah, USA) driven by the Femtopower amplifier served as the probe. The pump beam (centered at 735 nm, 18 nJ per pulse) was generated by an optical parametric amplifier (OPA; TOPAS, Light Conversion, Vilnius, Lithuania) driven by a Solstice amplifier. The pump and probe beams were overlapped and focused on the same spot in the sample. Each beam was interrupted by an optomechanical chopper on a shot-to-shot basis to acquire all four possible pulse combinations (pumped, not-pumped, dark background, and pump-only). The spectrum of the probe beam that passed through the sample was captured by a homemade prism spectrometer with a 1 kHz CCD camera (Entwicklungsbuero Stresing, Berlin, Germany). To reduce noise caused by white-light fluctuations, a second identical detector was used to acquire a reference spectrum of the probe replica without the sample and to perform a correction. During all experiments, the sample was kept in a 1-mm thick optical cell. 361 exponentially spaced time delays ranging from 10 fs to 0.6 ms were implemented. All experiments were conducted under the magic angle (54.7°) condition to eliminate orientation relaxation effects.

### Femtosecond stimulated Raman spectroscopy (FSR Spectroscopy)

A femtosecond-stimulated Raman spectroscopy setup employs two independent 1 kHz chirped pulse amplifiers (CPAs) seeded with fs pulses from one shared Ti:sapphire oscillator. The seed pulses were delayed electronically and optically to trace processes beyond 6 ns. The 735 nm pulses (150 nJ found to be an optimal energy after testing the energy in the 20–200 nJ range) from OPA, driven by a Solstice amplifier focused into a 100 μm spot, were used as the actinic pump (phototrigger) with ≈50 fs (full-width half maximum) pulse duration. Meanwhile, by focusing a 1450 nm signal beam from a second OPA system on a moving CaF_2_ plate, we generated a white-light supercontinuum as a probe, and the probe was focused on the sample at a spot of approximately 50 μm. In the detection apparatus, the probe was split into two beams. One part was sent to a grating-based high-resolution imaging spectrograph (Acton, Princeton instruments) for Raman analyses in the 750–950 nm region. The other part was directed to a prism spectrograph to obtain transient absorption spectra in the 370–1200 nm range. In both spectrographs, a 58 × 1024 pixels CCD camera (Entwicklungsbuero Stresing) was used as a linear image sensor via operation in a full vertical binning mode. The cameras were triggered from the lasers at 1 kHz and provided shot-to-shot detection. The 800 nm fs pulses from the second amplifier passed through a home-built pulse shaper to create a series of frequency-locked ps pulses as the Raman pump, with a total of 96 wavelength-shifted Raman pumps. The energy of the Raman pump was 2 μJ. We implemented 98 exponentially spaced time delays from 10 fs to 51.2 µs to sample the photoinduced dynamics. All the experiments were taken under the magic-angle (54.7°) condition to remove the influence of orientation relaxation. To reduce the impact of photodamage, we moved the sample in the beam at a speed of approximately 10 cm s^–1^ in a sample scanner in the case of degassed samples. For aerated samples, a peristaltic pump and flow cuvette were used to continuously flow the sample through the beam path to reduce photoinduced damage. The path length in both cases was 1 mm, and the sample absorbance at the excitation wavelength was ≈1 (with a 1-mm optical path length). All steady-state stimulated Raman spectra were taken with the actinic pulse off. To ensure that photodegradation did not occur to a significant extent during the recording of the FSR spectra, we measured the UV-Vis spectra of the solutions before and after the FSR measurements.

### Femtosecond stimulated Raman spectroscopy data analysis—target analysis

The deconvolution (peak fitting) of both the ground state and transient Raman spectra was performed using OriginPro 2023, OriginLab Corporation software. Tools: peak and baseline → multiple peak fit (nonlinear curve fit) using the Levenberg Marquardt iteration algorithm with a Lorentz model, 500 iterations, and 1 × 10^–12^ tolerance set as a default. It is important to note that the X-scale output of the fit was changed to be the same as the input data, as Origin produces an X-axis following the peak shape by default. This was done under Settings → Fitted Curves → × Data Type: the same as the input data. In the case of FSR spectra, the baseline was fixed at 0. The adj. *R*^2^ of >0.98 was considered satisfactory. It was necessary to perform noise filtering for a few FSR spectra; this was done by applying the Savitzky-Golay smoothing (points of window: 15, polynomial order: 2). For target analysis, we created a custom function in Origin: Y = A × C1 + B × C2 + C × C3 + D × C4 + E × C5 + F × C6. Where Y represents an experimental FSR spectrum at the given time, A, B, C, D, E, and F are the extracted (constructed) transient Raman spectra of ***Z*3 Cy7,** ***Z*4 Cy7**, S_1_, T_1_, hot S_1_, and **Cy7**^**•+**^ species, respectively, as an input of the model. C1–C6 are the associated coefficients, the values by which the transient Raman spectra (A to F) need to be multiplied to make the final sum equal to the experimental FSR spectrum, and this is the output of the fit. The Levenberg Marquardt iteration algorithm was used with 500 iterations and a 1 × 10^–12^ tolerance set as a default. The photophysical/photochemical models for the temporal evolution of different species were created using the Bounds option in the function dialogue. This is accomplished by setting the range in which the C1–C6 coefficients have positive values via the Higher bounds values set to >0. The lower Bounds of 0 was fixed for all C1–C6, as it is unrealistic to expect species to have less than 0 population at any given time. Several chemically plausible models were constructed and applied to the experimental data (Figures S53 and S54). The output coefficients C1–C6 were fitted with an exponential fit (ExpGrowDec function) in order to obtain the approximate lifetimes of the species (Figures S55–S58).

### Computational methods

Cyanine structures have previously been successfully characterized at the density functional theory (DFT) level using range-separated functionals^85^. The choice of basis set influences the prototypical heptamethine cyanine geometries very little, as pointed out by Jacquemin et al.^86^. Therefore, we opted for the ωB97X-D/6-31G* combination. Jacquemin et al. also suggested that solvent-induced geometry relaxation tends to be small as well^86^. In preliminary computations, we tested these effects for the **all-*E*** **Cy7** structure. The effects of the solvent were modeled using the implicit solvation model IEF-PCM^87^ with acetonitrile, methanol, and water for structure optimization and subsequent Raman spectral modeling. Only a modest change in the calculated Raman shifts was observed compared to the gas-phase results. Therefore, we proceeded with all calculations of Raman spectra in the gas phase using the vibrational scaling factor of 0.9485^88^. However, we accounted for solvent effects in complexation energy calculations and superoxide solvent shift characterization. For these types of calculations, we chose a more advanced IEF-PCM/SMD (acetonitrile) model^87^.

Excited state geometries were characterized using time-dependent DFT (TDDFT) at the same level of theory. According to the literature, TDDFT fails to provide accurate transition energies for these systems, however, it tends to correctly describe the ground- and excited-state geometries and vibrational frequencies^89^, which play a key role in Raman spectra modeling. All optimizations and frequency calculations regarding Raman spectra modeling were performed using Q-Chem v5.4.1 quantum chemistry package^90,91^.

## Supplementary information

Supplementary Information

## Data Availability

The raw datasets generated and/or analyzed during the current study are available from the corresponding authors on reasonable request.
