# Supplementary material for: The first microseconds of the life of excited heptamethine cyanine revealed by femtosecond stimulated Raman spectroscopy
Source: Commun Chem. 2025 Dec 15;9:43. doi: 10.1038/s42004-025-01850-2 (PMC12827978; doi:10.1038/s42004-025-01850-2)
Supplement: Supplementary file 1 — Supplementary Information [file 42004_2025_1850_MOESM1_ESM.pdf]

Supporting Information

The First Microseconds of the Life of Excited Heptamethine Cyanine Revealed by Femtosecond Stimulated Raman Spectroscopy

Gabriel Glotz[1,2]*, Jan Polena[3], Nasrulla Majid Khan[1,2], Atripan Mukherjee[4], Miroslav Kloz[4], Petr Slavíček[3], and Petr Klán[1,2]*

[1] Department of Chemistry, Faculty of Science, Masaryk University, Kamenice 5, 62500 Brno, Czech Republic
[2] RECETOX, Faculty of Science, Masaryk University, Kamenice 5, 62500 Brno, Czech Republic
[3] Department of Physical Chemistry, University of Chemistry and Technology, Prague, Technická 5, 16628 Prague 6, Czech Republic
[4] The Extreme Light Infrastructure Facility ERIC, Za Radnicí 835, 25241 Dolni Břežany, Czech Republic

Contents

1. General information 1
   1.1. Materials and sample preparation 1
   1.2. Femtosecond transient absorption spectroscopy (fs-TA) 1
   1.3. Femtosecond stimulated Raman spectroscopy 1
   1.4. FSR spectroscopy data analysis in Origin – target analysis 3
   1.5. Computational details 4
   1.6. Calculation of ΔG_{PET} 5
   1.7. Transient Raman spectrum of cyanine radical dication (**Cy7**$^{\bullet+}$) 5
2. Ground state stimulated Raman spectra 5
   2.1. Ground state Raman spectrum of **Cy7** in acetonitrile 6
   2.2. Ground state Raman spectrum of **Cy7** in methanol 6
   2.3. Ground state stimulated Raman spectra of deuterated **Cy7** derivatives in methanol 7
   2.4. Ground state stimulated Raman spectrum of **Cy7** in water with 5% methanol 8
   2.5. Comparison of ground state Raman spectra 9
3. Femtosecond transient absorption spectroscopy (fs-TA) of **Cy7** 10
4. UVVis spectra of O_2-**Cy7** ground state complex 13
5. Computational data 14
   5.1. Calculated Raman spectra for **all-*E* Cy7** 14
   5.2. Calculated Raman spectra of **Cy7**$^{\bullet+}$ with all-*E* configuration 15
   5.3. Calculated Raman spectra of *Z1* – **Cy7** 16
   5.4. Calculated Raman spectra of ***Z2*** – **Cy7** 17
   5.5. Calculated Raman spectra of ***Z3*** – **Cy7** 18
   5.6. Calculated Raman spectra of ***Z4*** – **Cy7** 19
   5.7. Comparison of calculated Raman spectra 20
   5.8. Interpretation of calculated Raman spectra 24
   5.9. Calculated potential energy surfaces 26
6. FSR spectra 30
   6.1. Experimental FSR spectra of **Cy7** in acetonitrile 30
   6.2. TR-Raman spectra of species formed upon **Cy7** excitation 35
   6.3. Superoxide radical anion 38
   6.4. TR-Raman spectra of **Cy7**$^{\bullet+}$ / the DCA experiment 41
7. Target analysis 44
   7.1. Target analysis models 45
   7.2. Fitted FSR data 47
8. Synthesis of **Cy7**-2',6'-d_2 49
   8.1. NMR spectra 51
9. Cartesian Coordinates for structures optimized at the ωB97X-D/6-31G* level of theory 55
10. References 75

1. General information

1.1. Materials and sample preparation

Acetonitrile and methanol of the highest purity available were used as purchased. Deionized water used was obtained using a Milli-Q® system. 9,10-Dicyanoanthracene (97%) was purchased and recrystallized from toluene. All cyanine derivatives were synthesized and purified according to our previously published work from the corresponding pyridine derivatives via the Zincke reaction.[1] Di-(**Cy7**-2′,6′-d_2) and pentadeuteriated (**Cy7**-2′,3′,4′,5′,6′-d_2) **Cy7** derivatives were prepared from commercially available pyridine-d_5 or from pyridine-2,6-d_2. The latter derivative was prepared from commercially available 2,6-dibromopyridine according to the literature procedure (page 39 for details).[2] The analytical data of the deuterated cyanine derivatives were in line with literature values.[3]

Flash column chromatography was performed using silica gel (230–400 mesh). ^{1}H NMR spectra were recorded on a 500 MHz spectrometer; ^{13}C NMR spectra were obtained on a 125 MHz instrument in DMSO-d_6 or CD_3OD. ^{1}H chemical shifts are reported in ppm relative to tetramethylsilane (δ = 0.00 ppm) using the residual solvent signal as an internal reference. ^{13}C chemical shifts are reported in ppm using DMSO-d_6 (δ = 39.52 ppm) and CD_3OD-d_4 (δ = 49.00 ppm) as internal references. The deuterated solvents were kept under nitrogen atmosphere.

Sample solutions for FSR spectroscopy measurements (A = 1 ± 0.05 at 735 nm / 1 mm optical path length) were prepared by diluting 100 mM stock solutions prepared in the corresponding solvents. For water solutions, the addition of methanol (5%) was necessary to increase the solubility of **Cy7** derivatives. The degassed samples were prepared using the freeze-pump-thaw method; the cycles were repeated until there was no significant pressure change; at least 4 cycles).

1.2. Femtosecond transient absorption spectroscopy (fs-TA)

fs-TA measurements were conducted using homebuilt 1-kHz transient absorption and femtosecond-stimulated Raman spectroscopy setups constructed around femtosecond Ti:sapphire amplifiers Femtopower (Spectra Physics) and Solstice amplifier (Spectra Physics), which shared a common oscillator. The amplifiers were synchronized by electronic triggering and an optical delay of the seed prior to amplification, allowing setting the delay between their pulses up to <1 ms with fs precision. Two laser beams, a pump and a probe, were used in the TA experiment. White light supercontinuum generated in an argon-filled hollow core fiber (Ultrafast Innovations, Savannah, USA) driven by the Femtopower amplifier served as the probe. The pump beam (centered at 735 nm, 18 nJ per pulse) was generated by an optical parametric amplifier (OPA; TOPAS, Light Conversion, Vilnius, Lithuania) driven by a Solstice amplifier. The pump and probe beams were overlapped and focused on the same spot in the sample. Each beam was interrupted by an optomechanical chopper on a shot-to-shot basis to acquire all four possible pulse combinations (pumped, not-pumped, dark background, and pump-only). The spectrum of the probe beam that passed through the sample was captured by a homemade prism spectrometer with a 1 kHz CCD camera (Entwicklungsbuero Stresing, Berlin, Germany). To reduce noise caused by white light fluctuations, a second identical detector was used to acquire a reference spectrum of the probe replica without the sample, and a correction was performed as described in the literature.[4] During all experiments, the sample was kept in a 1-mm thick optical cell. 361 exponentially spaced time delays ranging from 10 fs to 0.6 ms were implemented. All experiments were conducted under the magic angle (54.7°) condition to eliminate orientation relaxation effects.

1.3. Femtosecond stimulated Raman spectroscopy

A femtosecond-stimulated Raman spectroscopy setup is an upgraded version[5] of the one described in our previous work.[6] In brief, in the current design, we seeded two independent 1 kHz chirped pulse amplifiers (CPAs) with fs pulses from one shared Ti:sapphire oscillator. The seed pulses were delayed electronically and optically to trace processes beyond six ns. The 735 nm pulses (150 nJ found to be an optimal energy after testing the energy in the 20–200 nJ range). from OPA driven by a Solstice amplifier focused into a 100 µm spot were used as the actinic pump (phototrigger) with ≈50 fs (full-width half maximum) pulse duration. Meanwhile, by focusing a 1450 nm signal beam from a second OPA system on a moving CaF_2 plate, we generated a white-light supercontinuum as a probe, and the probe was focused on the sample at a spot of approximately 50 µm. In the detection apparatus, the probe was split into two beams. One part was sent to a grating-based high-resolution imaging spectrograph (Acton, Princeton instruments) for Raman analysis in

the 750–950 nm region. The other part was directed to a prism spectrograph to obtain transient absorption spectra in the 370–1200 nm range. In both spectrographs, a 58×1024 pixels CCD camera (Entwicklungsbuero Stresing) was used as a linear image sensor via operation in a full vertical binning mode. The cameras were triggered from the lasers at 1 kHz and provided shot-to-shot detection. The 800 nm fs pulses from the second amplifier passed through a home-built pulse shaper to create a series of frequency-locked ps pulses as the Raman pump, with a total of 96 wavelength-shifted Raman pumps. The energy of the Raman pump was 2 µJ. We implemented 98 exponentially spaced time delays from 10 fs to 51.2 µs to sample the photoinduced dynamics. All the experiments were taken under the magic-angle (54.7°) condition to remove the influence of orientation relaxation. To reduce the impact of photodamage, we moved the sample in the beam at a speed of approximately 10 cm/s in a sample scanner in the case of degassed samples. For aerated samples, a peristaltic pump and flow cuvette were used to continuously flow the sample through the beam path to reduce photoinduced damage. The path length in both cases was 1 mm, and the sample absorbance at the excitation wavelength was ≈1 (with a 1-mm optical path length). All steady-state stimulated Raman spectra were taken with the actinic pulse off. To ensure that photodegradation did not occur to a significant extent during the recording of the FSR spectra, we measured the UV-Vis spectra of the solution both before and after the FSR measurements were taken.

Comment on the energy of the Raman pulse. Raman-pulse energy values are strongly influenced by two parameters that are difficult to characterize with high accuracy: the Raman spot size and the pulse duration. The experimental setup used in this study was optimized for long focal lengths and thick samples (up to ~3 mm). Consequently, the Raman spot size was larger than that in many other setups. Additionally, the Raman pulse in our system was narrower and longer than is common in the field. Rather than the usual 1 ps duration, ours was closer to 3 ps. Using the existing setup, we conducted experiments with Raman pulse energies up to 8 µJ without encountering significant issues. This applies to the off-resonant regime. Under resonant conditions, the FSR signals are strong enough that substantially lower pulse energies can be used, as was the case for the system studied here. A major limitation is the Gaussian temporal profile of the Raman pulse. Ideally, the Raman pump in FSRS would have an asymmetric Lorentzian temporal profile; however, producing such a shape is difficult with the amplitude-only pulse-shaping capabilities available in our setup. The leading Gaussian tail of the Raman pulse can deplete the excited state, reducing the absolute signal magnitude. Since this depletion depends on the temporal evolution of the excited state itself, it can introduce spurious kinetics. For this reason, fs-TA was always measured simultaneously in our setup. This enabled us to compare the unperturbed fs-TA dynamics with the Raman-pulse-perturbed FSR dynamics. FSR experiments always involved a compromise between maximizing the signal-to-noise ratio and operating in a perfectly bilinear regime, which is rarely fully achievable. Raman-pulse energies in the range of hundreds of nJ were used, with energies close to 1 µJ being more common. However, these typically use shorter, more nonlinear Raman pulses and focusing geometries optimized for thinner samples. In our opinion, the best indicator of the actual degree of nonlinearity is the absolute FSRS signal magnitude. Since our signals were generally in the sub-mOD range, we consider the presence of substantial nonlinearities to be unlikely. Ultimately, the consistency of interpretations based on combined FSRS and TA measurements is the most reliable arbiter in each case.

Data analysis. Our setup was designed to use 4 pulses out of each 100 and 96 to measure fs-TA and FSR spectroscopies, respectively. This setup is motivated by much weaker signals observed in a typical FSR spectroscopy experiment compared to that of fs-TA experiment running in the background. A dwell time of 2 min was used for each time delay between the pump and the probe, which means that the fs-TA spectra are the result of an average of ≈5 s (5000 laser shots) and the FSR spectra are the result of an average of ≈115 s (115 000 shots) average. However, both the FSR spectroscopy and fs-TA methods run effectively in parallel within a single experiment. To reduce the raw data production, an on-fly algorithm was used to reject 15% of the most outstanding shots and to apply a mathematical mean to the remaining ones. The processed data from the fs-TA methods was globally fitted by the previously described homemade software[7,8] written in Python, using a partitioned variable projection algorithm.[9,10] Coherent artifacts (cross-phase modulation, stimulated Raman scattering) resulting from the overlap of the pump and probe pulses in time and space were modeled as a Gaussian function and its derivatives.[11,12] The presented data are corrected for group velocity dispersion (chirp) by linear interpolation, but the fit was performed on the raw data.

Quantum yields. Since the Raman cross section and total concentrations of the short-lived species are unknown, the quantum yields for their formation could not be determined. The ratio of ground-state bleach before and after the process is often used to estimate the relative quantum yield of the transition. However, spurious bleaching, which often occurs in FSR spectra, and signal overlap cancel out part of the ground-state signal, making it appear smaller. Therefore, we did not calculate relative quantum yield ratios.

1.4. FSR spectroscopy data analysis in Origin – Target analysis

The deconvolution (peak fitting) of both the ground state and transient Raman spectra was performed using OriginPro2023 (64-bit), 10.0.0.154 (Academic), OriginLab Corporation software. Tool: peak and baseline → multiple peak fit (nonlinear curve fit) using the Levenberg Marquardt iteration algorithm with a Lorentz model, 500 iterations, and 1×10^{-12} tolerance set as a default. It is important to note that the X-scale output of the fit was changed to be the same as the input data, as Origin produces an X-axis following the peak shape by default. This was done under Settings → Fitted Curves → x Data Type: the same as the input data. In the case of FSR spectra, the baseline was fixed at 0. The adj. R^2 of >0.98 was deemed satisfactory. It was necessary to perform noise filtering for a few FSR spectra; this was done by applying the Savitzky-Golay smoothing (points of window: 15, polynomial order: 2). For target analysis, we created a custom function in Origin: Y = A×C1 + B×C2 + C×C3 + D×C4 + E×C5 + F×C6. Where Y represents an experimental FSR spectrum at the given time, A, B, C, D, E, and F are the extracted (constructed) transient Raman spectra of ***Z*3 Cy7**, ***Z*4 Cy7**, S_1, T_1, hot S_1, and **Cy7**$^{\bullet+}$ species respectively, as an input of the model. The C1–C6 are the associated coefficients, the values by which the transient Raman spectra (A to F) need to be multiplied to make the final sum equal to the experimental FSR spectrum, and this is the output of the fit. The Levenberg Marquardt iteration algorithm was used with 500 iterations and a 1×10^{-12} tolerance set as a default. For details on how to create a custom function, see page 135 of the Origin tutorial pdf document.[13] The photophysical/photochemical models for the temporal evolution of different species were created using the Bounds option in the function dialogue. This is accomplished by setting the range in which the C1–C6 coefficients have positive values via the Higher bounds values set to >0. The lower Bounds of 0 was fixed for all C1–C6, as it is unrealistic to expect species to have less than 0 population at any given time. Several chemically plausible models were constructed and applied to the experimental data (Figures S53 and S54). The output coefficients C1–C6 were fitted with an exponential fit (ExpGrowDec function) in order to obtain the approximate lifetimes of the species (Figures S55 – S58).

1.5. Computational details

Cyanine structures have previously been successfully characterized at the density functional theory (DFT) level using range-separated functionals.[14] The choice of basis set influences the prototypical heptamethine cyanine geometries very little, as pointed out by Jacquemin et. al.[15] Therefore, we opted for the *ω*B97X-D/6-31G* combination. Jacquemin et al. also suggested that solvent-induced geometry relaxation tends to be small as well.[15] In preliminary computations, we tested these effects for the ***all-E*** **Cy7** structure. The effects of the solvent were modeled using the implicit solvation model IEF-PCM[16] with acetonitrile, methanol, and water for structure optimization and subsequent Raman spectral modeling. Only a modest change in the calculated Raman shifts was observed compared to the gas-phase results. Therefore, we proceeded with all calculations of Raman spectra in the gaseous phase using the vibrational scaling factor of 0.9485.[17] However, we accounted for solvent effects in complexation energy calculations and superoxide solvent shift characterization. For these types of calculations, we choose a more advanced IEF-PCM/SMD(Acetonitrile) model.[16]

Excited state geometries were characterized using time-dependent DFT (TDDFT) at the same level of theory. According to the literature, TDDFT fails to provide accurate transition energies for these systems, however, it tends to correctly describe the ground and excited state geometries and vibrational frequencies,[18] which play a key role in Raman spectra modeling. All optimizations and frequency calculations regarding Raman spectra modelling were performed using Q-Chem v5.4.1 quantum chemistry package.[19,20]

The superoxide Raman shift solvation effect was determined using CCSD(T)/aug-cc-pVQZ level of theory. The calculated Raman shifts were scaled by a factor of 0.961.[21] We modelled solvation effects as a dielectric continuum via IEF-PCM/SMD(Acetonitrile). The calculations were conducted in Gaussian 16, rev. A.03.[22]

The complexation energy was calculated as a sum of the gas phase Gibbs energy, the solvation energy, and the correction term corresponding to different bonding regions of the oxygen to cyanine structure.[23] The gas phase Gibbs energy was calculated as the sum of DLPNO-CCSD(T)/cc-pVTZ electronic energies and ωB97X-D/6-31G* Gibbs correction energy terms using ωB97X-D/6-31G* geometries. The solvation energy was calculated via the IEF-PCM/SMD(Acetonitrile) model on the M05-2X/6-31G* level of theory. The Gibbs energy correction term was calculated using ωB97X-D/6-31G* level of theory as:

$$\Delta G_{\text{corr.}} = -RT \ln\left(\sum_i \sigma e^{-\frac{E_i - E_{\min}}{RT}}\right),$$

where E_i is the electronic energy of a conformer, $E_{\min}$ is the electronic energy of the global minimum structure and σ is the symmetry factor corresponding to the number of equivalent structures.

First, we used the GOAT algorithm in combination with the XTB level of theory in ORCA 6.0.1 for the localization of possible minima.[24,25] Subsequently, we reoptimized the minima at the ωB97X-D/6-31G* level and discarded the duplicate and isomeric structures. The remaining structures were used for the calculation of the correction term (the Cartesian coordinates of the structures are presented in section 9). The DLPNO-CCSD(T) calculations were conducted in ORCA 6.0.1 quantum chemistry package, whereas DFT calculations were conducted in Gaussian 16, rev. A.03.

Photoisomerization potential energy surface (PES) schemes were constructed as recalculated energies for stationary points obtained at the CIS(D)/TZVP level of theory using the ωB97X-D/6-31G* method (combined with Int=UltraFine option) optimized geometries. The combination of CIS(D)/TZVP vertical excitation energies and DFT/(small basis set) geometries is a recommended approach for vertical excitation energy calculation by Jacquemin et al. for prototypical heptamethine cyanine **Cy7**.[18] We also show PES schemes calculated at the TD-ωB97X-D/6-31G* // ωB97X-D/6-31G* level of theory for a comparative purpose. All calculations regarding the PES schemes were conducted in Gaussian 16, rev. A.03. The Cartesian coordinates of optimized structures are shown further in the text (see section 9).

1.6. Calculation of ΔG_{PET}

Photoinduced electron transfer between ***Cy7*** *(S_1) and ground state O_2.* The ΔG_{PET} was calculated according to the formula $\Delta G_{PET} = -F(E_{red}(S/S^{\bullet -}) - E^*_{OX}(pc^{\bullet +}/pc^*))$,[26] where $\boldsymbol{F}$ is the Faraday constant (23.061 kcal V^{-1} mol^{-1}), E_{red} is the ground-state redox potential of the substrate ***S*** (O_2 in this case), and E^*_{OX} is the excited state oxidation potential of photocatalyst ***pc*** (**Cy7**). The first step was to find the E^*_{OX} of **Cy7**; the value of –0.99 V *vs* NHE (in CH_3CN)[27] was subtracted from the value of $E_{0,0}$ (1.48 eV),[27] providing E^*_{OX} = –0.49 V *vs* NHE. The E_{red} value for O_2 was obtained from the literature (–1.00 V *vs* SCE in CH_3CN/0.1 M tetraethylammonium perchlorate, with glassy carbon electrode)[28] and recalculated to be –0.75 V vs NHE. Finally, the value of –6.92 kcal mol^{-1} for ΔG_{PET} was obtained. The electrostatic term was considered to have minimal contributions and was not accounted for in the calculation.

1.7. Transient Raman spectrum of cyanine radical dication ($Cy7^{\bullet +}$)

The ΔG_{PET} was calculated in a similar manner to the previous one, $\Delta G_{PET} = -F(E^*_{red}(pc^*/pc^{\bullet -}) - E_{ox}(S^{\bullet +}/S))$. Here, the S_1 state of DCA is photoreduced, and ground-state **Cy7** is oxidized. The DCA E^*_{red} = +2.24 V *vs* NHE was obtained from the literature,[26] and E_{ox} –0.56 V *vs* NHE (in CH_3CN)[27] was used, providing ΔG_{PET} = –64.57 kcal mol^{-1}. The electrostatic term was considered to have minimal contributions and was not accounted for in the calculation. Many other cyanine radical (di)cations have been prepared using chemical and electrochemical oxidation and characterized by absorption and EPR spectroscopies.[29] The **Cy7**$^{\bullet +}$ was shown to be a short-lived radical, with the lifetime shorter than ≈1 µs, estimated from the lack of continuous wave EPR detection.[30] Unfortunately, this made it impossible for us to record its Raman spectrum through its chemical or electrochemical production. Our experiment features the same FSR spectroscopy setup as that used for the **Cy7** study, except that the actinic (pump) wavelength was set to 360 nm, which is the wavelength where DCA has very high and **Cy7** has very low absorbance (ratio of *A* >10, Figure S50). In this way, the actinic pulse excites mostly DCA molecules initiating PET and producing **Cy7**$^{\bullet +}$ which due to the resonance conditions with the 800 nm Raman pulse results in an enhanced Raman signal intensity for **Cy7**$^{\bullet +}$ (Figure S51). The FSR spectroscopy measurement with DCA and **Cy7** was performed under degassed conditions in acetonitrile.

2. Ground state stimulated Raman spectra

Cy7 is a vibrationally very active molecule with many energetically close and mechanically coupled vibrational modes. Therefore, our ground-state Raman spectrum assignment focused on higher amplitude vibrations. The experimental ground state Raman (GSR) spectra were recorded in acetonitrile (Figure S1), methanol (Figure S2), and water with 5% methanol as a co-solvent to enhance solubility (Figure S5). The assignment of all bands was supported by calculations (see Table S1 in section 5.8). An intense vibrational band at 1618 cm^{-1} was attributed to the C-C stretching in aromatic rings coupled with stretching vibration of the C=C bond (ν_s) with the *E*-configuration (Table S1**,** entry 1 and 2). The corresponding C–H in-plane bending ($\delta_{i.p.}$) vibrational band of the polyene chain is located at 1309 cm^{-1} (Table S1, entry 11). The experimental assignment of the C–H $\delta_{i.p.}$ band was supported by GSR recordings of Di- (**Cy7**-2',6'-d_2, Figure S3) and pentadeuteriated (**Cy7**-2',3',4',5',6'-d_5, Figure S4) **Cy7** derivatives. In addition, the deuterated derivatives served a secondary purpose by replacing the C–H δ(i.p.) with C–D $\delta_{i.p.}$, energetically decoupling it from C=C ν_s and allowing us to evaluate which of the vibrational bands in the parent **Cy7** arose, because C-H $\delta_{i.p.}$ and C=C ν_s form a coupled oscillator. The remaining bands in the GSR are C–H vibration (δ_{as}) of N–CH_3 found at 1478 cm^{-1} (Table S1 entry 4). The C–H deformation vibration (δ_s) bands of N–CH_3 were located at 1477 cm^{-1} along with two bands of approximately equal intensity for the dimethylmethylene moiety at 1381 cm^{-1} and 1413 cm^{-1} (Table S1 entry 5 and 9). These bands are of the same intensity in water and methanol as solvents, whereas in acetonitrile, a lower energy band has additional contributions from the CH_3 vibrational mode. The benzene ring vibrational bands: quadrant stretching (1552 cm^{-1} Table S1, entry 1 and 2), semi-circle stretching and C–H in-plane bending vibrational bands (1238, 1180, 1142, 1087, 1032, cm^{-1} Table S1 entry 12 – 18), and C–H out-of-plane bending vibrational band at 915 cm^{-1} (Table S1 entry 19). In general, the overall differences in GSR between solvents are small and mainly limited to the relative differences in the band intensities between different solvents (Figure S7).

2.1. Ground state Raman spectrum of Cy7 in acetonitrile

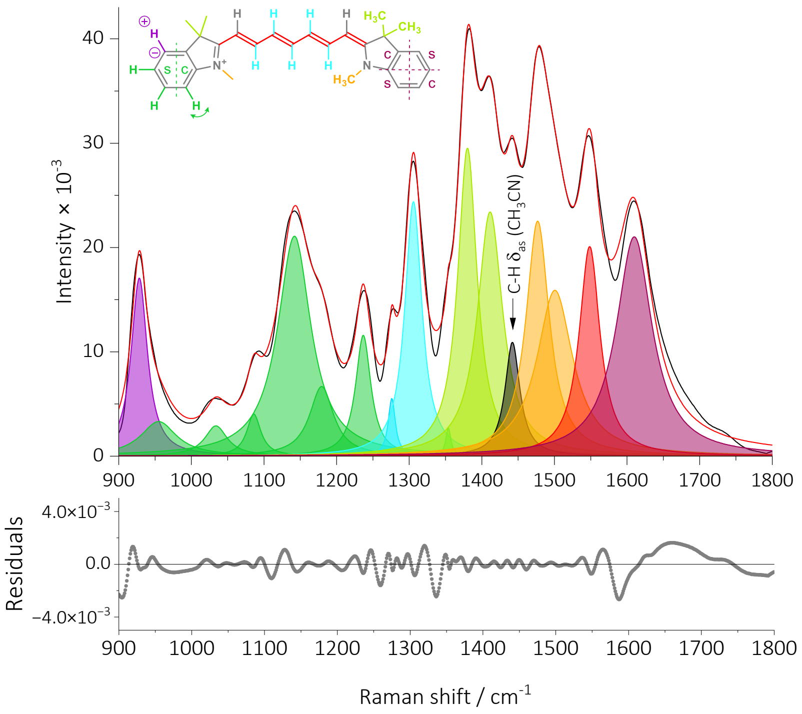

Figure S1. Deconvoluted experimental ground-state Raman spectrum of **Cy7** in acetonitrile (black line), cumulative fit (red line) and (below). The difference between the fitted and experimental spectra, as well as the broad residual peak above 1600 cm^{-1}, is due to peak broadening caused by the baseline correction method, this difference does not contain any signals.

2.2. Ground state Raman spectrum of Cy7 in methanol

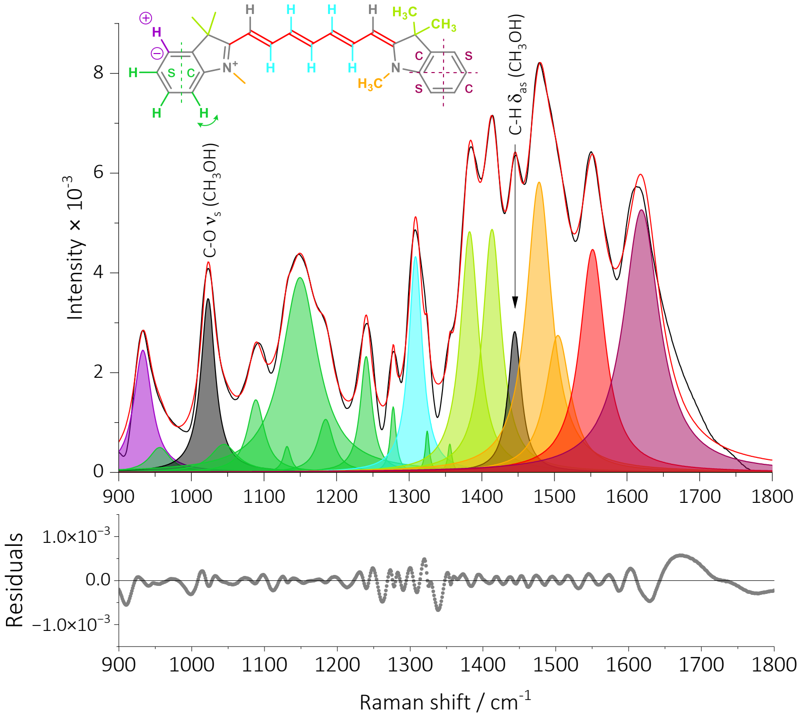

Figure S2. Deconvoluted experimental ground-state stimulated Raman spectrum of **Cy7** in methanol (black line), cumulative fit (red line) and fitting residuals (below). The difference between the fitted and experimental spectra, as well as the broad residual peak above 1600 cm^{-1}, is due to peak broadening caused by the baseline correction method, this difference does not contain any signals.

2.3. Ground state stimulated Raman spectra of deuterated Cy7 derivatives in methanol

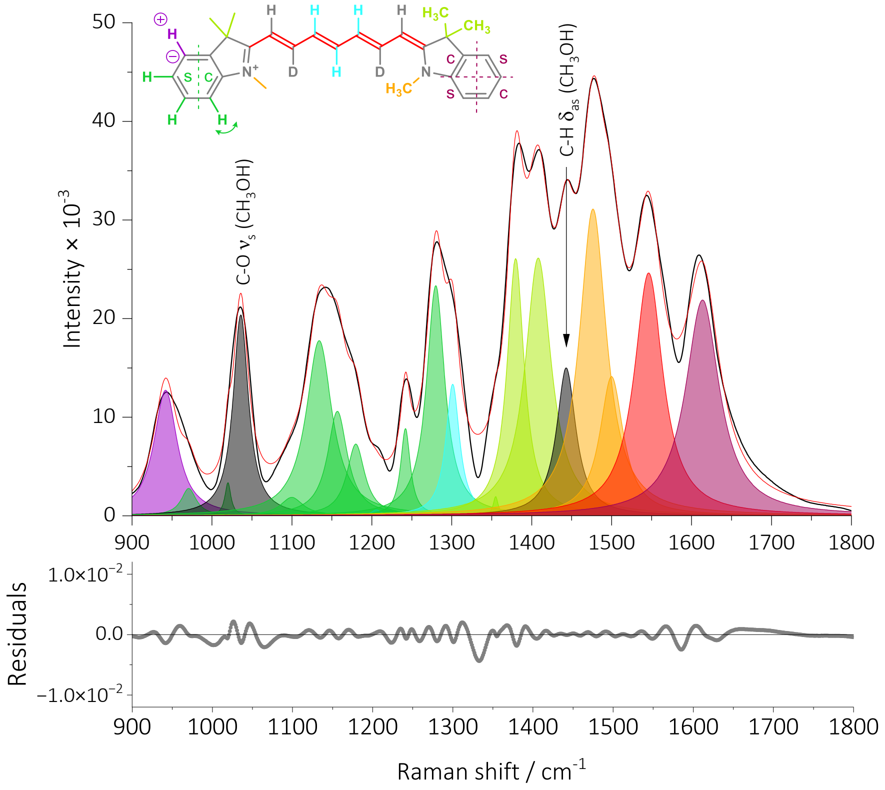

Figure S3. Deconvoluted experimental ground-state stimulated Raman spectrum of **Cy7**-2’,6’-d_2 in methanol (black line), cumulative fit (red line) and fitting residuals (below).

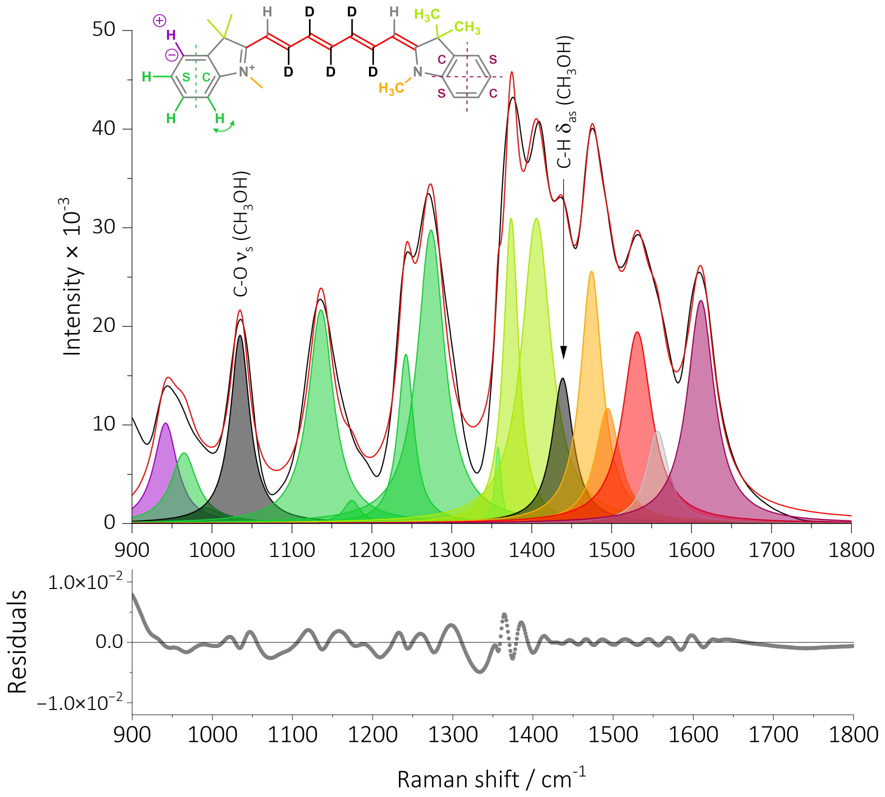

Figure S4. Deconvoluted experimental ground-state stimulated Raman spectrum of **Cy7**-2’,3’,4’,5’,6’-d_5 in methanol (black line), cumulative fit (red line) and fitting residuals (below).

2.4. Ground state stimulated Raman spectrum of Cy7 in water with 5% methanol

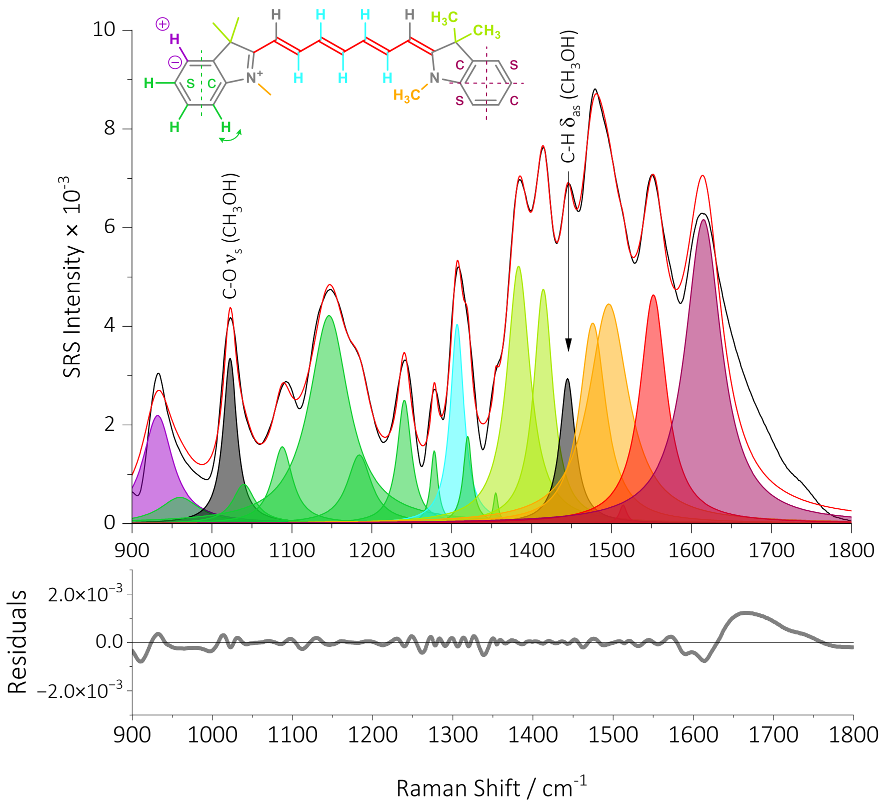

Figure S5. Deconvoluted experimental ground-state stimulated Raman spectrum of **Cy7** in water (black line), cumulative fit (red line) and fitting residuals (below). The difference between the fitted and experimental spectra, as well as the broad residual peak above 1600 cm^{-1}, is due to peak broadening caused by the baseline correction method, this difference does not contain any signals.

2.5. Comparison of ground state Raman spectra

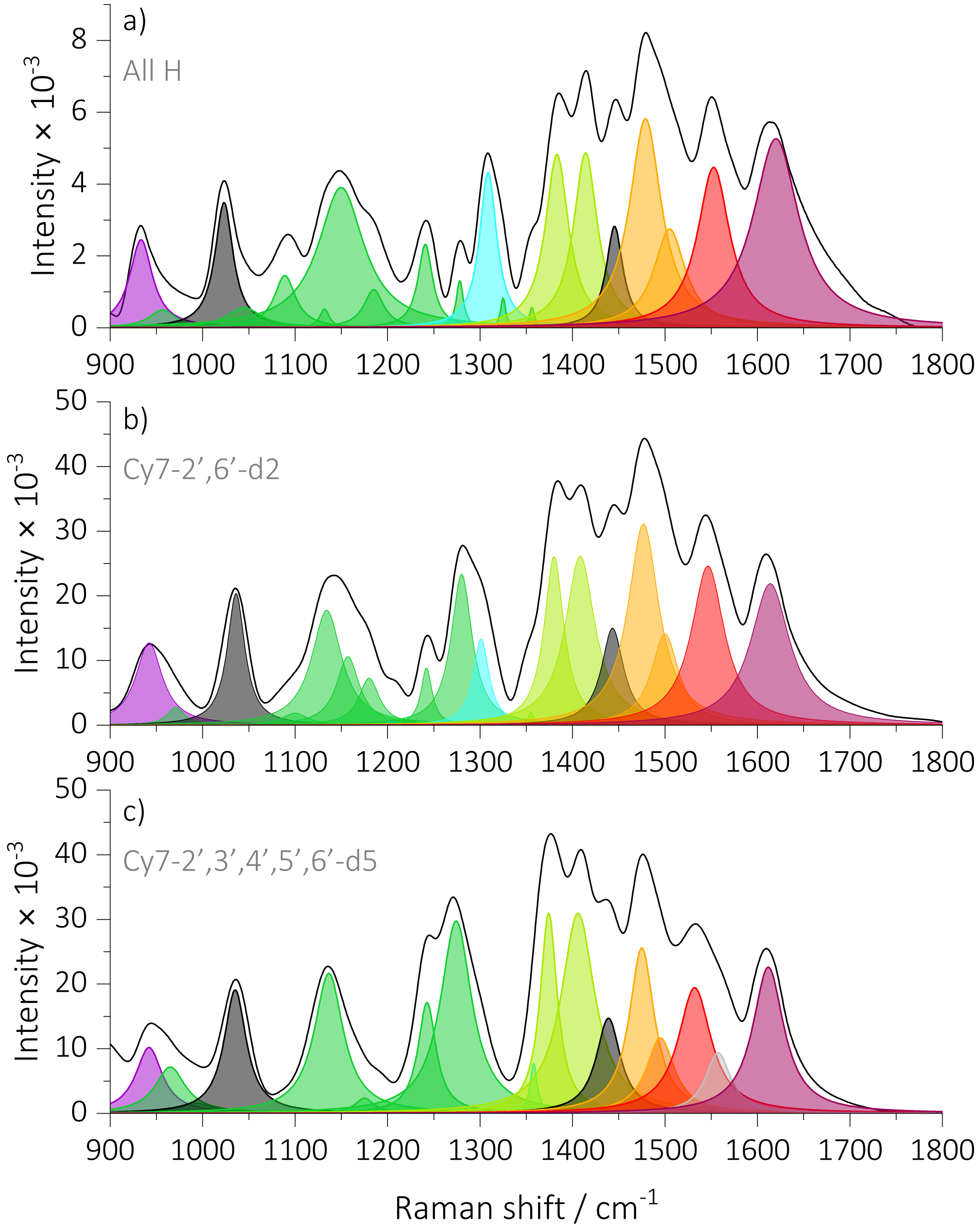

Figure S6. Comparison of deconvoluted experimental ground-state stimulated Raman spectra of a) **Cy7**, b) **Cy7**-2',6'-d_2 and c) and **Cy7**-2',3',4',5',6'-d_5 in methanol.

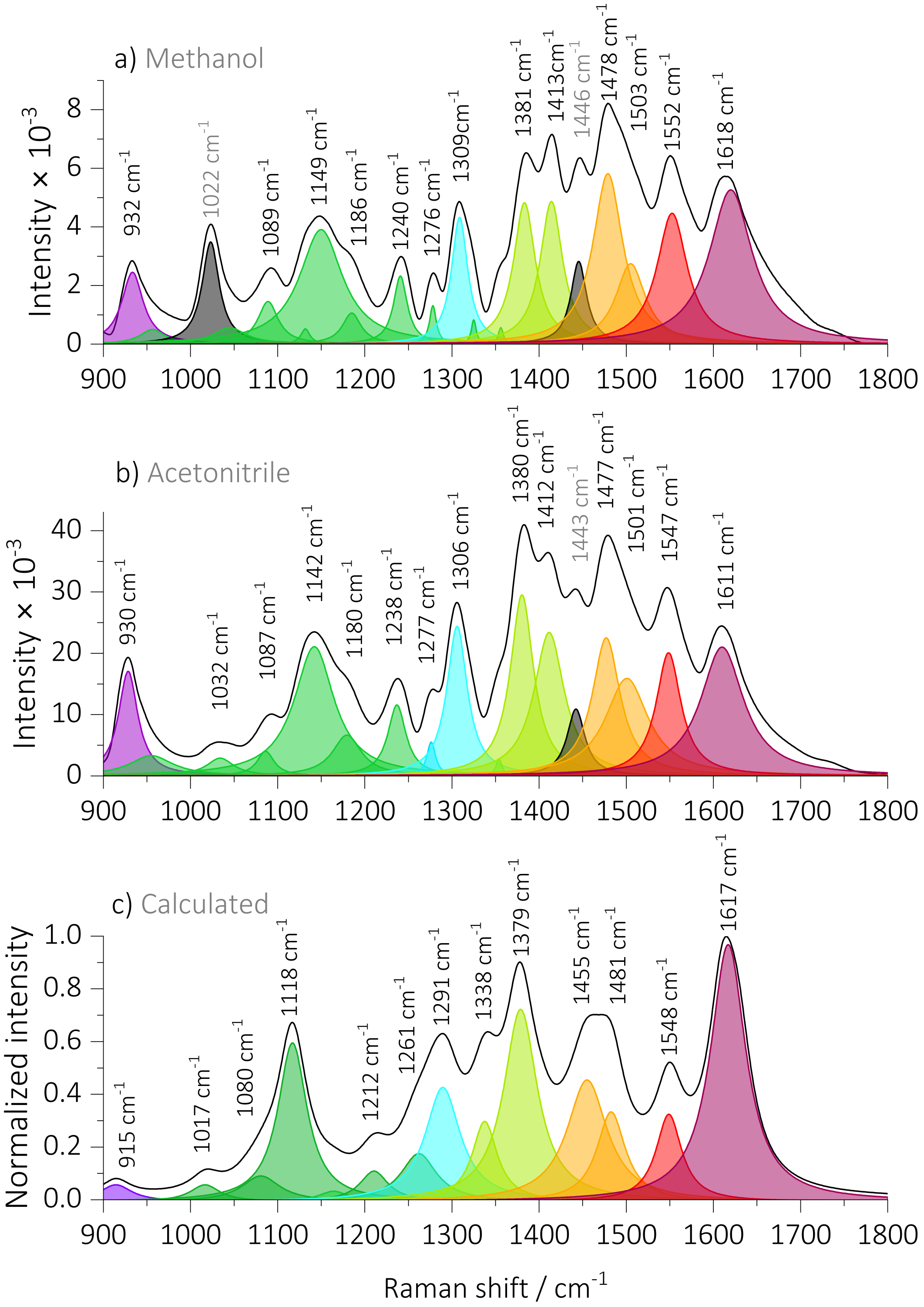

Figure S7. Comparison of deconvoluted experimental ground-state stimulated Raman spectra of all hydrogen **Cy7** in; a) methanol and b) acetonitrile, and c) calculated Raman spectra of ***all-E*** **Cy7**.

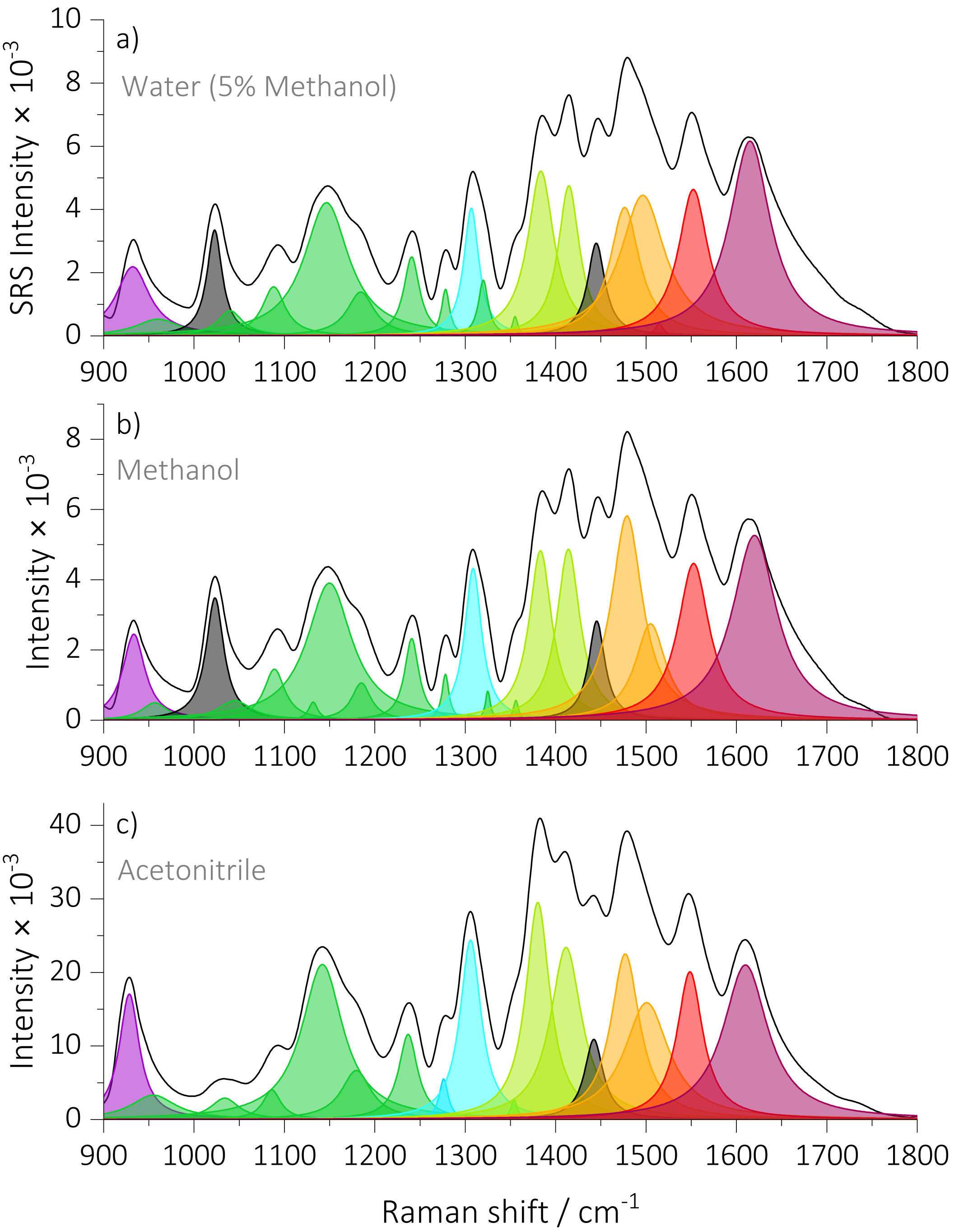

Figure S8. Comparison of deconvoluted experimental ground-state stimulated Raman spectra of **Cy7** in; a) water with 5% methanol, b) methanol and c) acetonitrile.

3. Femtosecond transient absorption spectroscopy (fs-TA) of Cy7

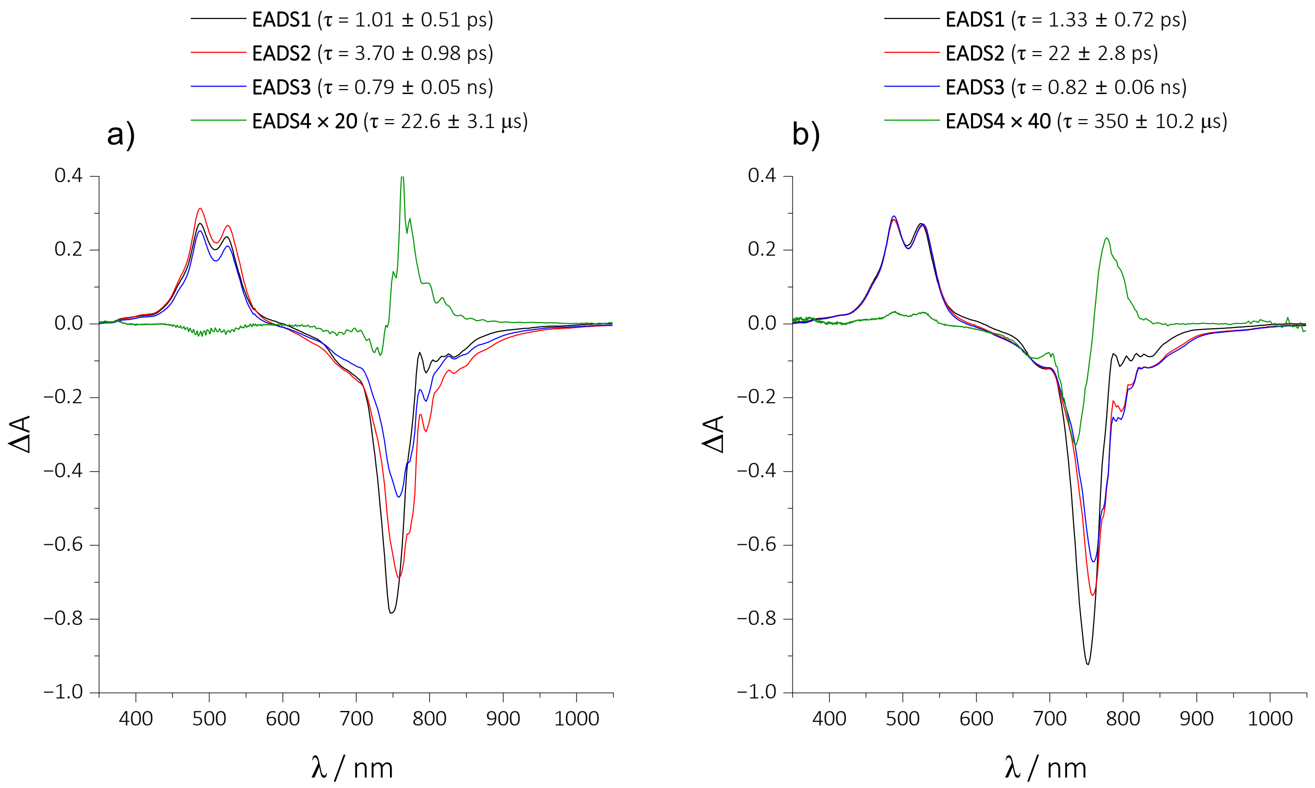

Figure S9. The fs-TA data of **Cy7** in a) aerated and b) degassed methanol.

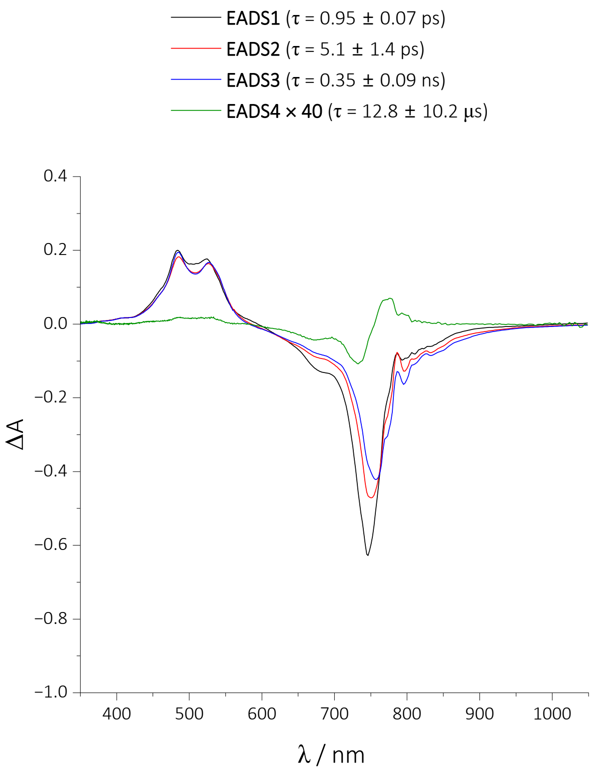

Figure S10. The fs-TA data of **Cy7** in aerated water (with 5% methanol for solubility).

4. UVVis spectra of O_2–Cy7 ground state complex

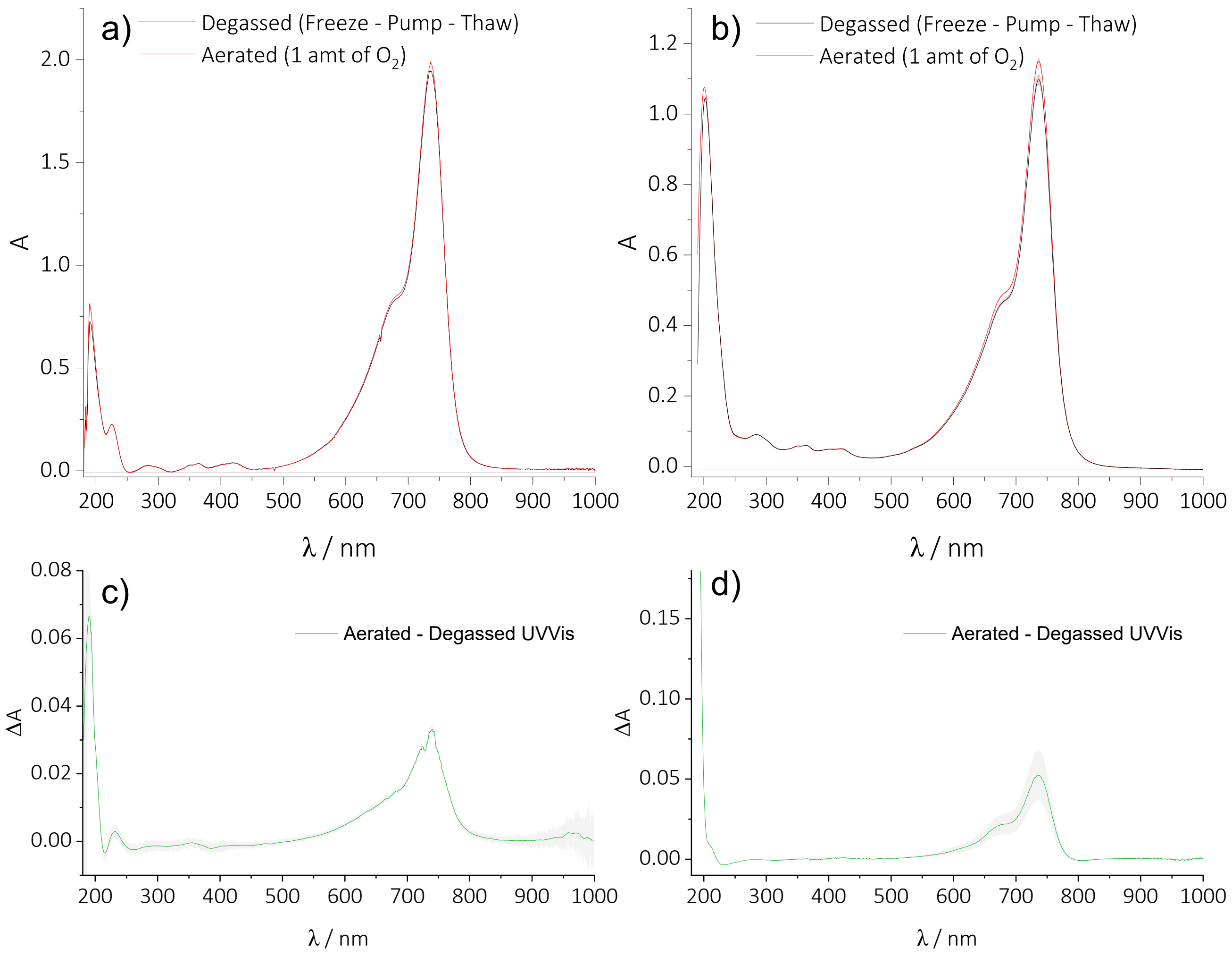

Figure S11. Absorption spectra of **Cy7** in aerated (1 atm of O_2, red line) and degassed (>4 cycles of freeze–pump–thaw, black line) acetonitrile. The figures a) and b) show the UV-Vis spectra of **Cy7** in the presence and absence of O_2, while figures c) and d) are the difference spectra of aerated vs. degassed samples from the same experiments. Measurements were taken using an instrument that had been powered on for at least two hours in a temperature-controlled room to stabilize the light source. The cuvette was placed in the same position each time. Due to **Cy7** adsorption on the walls of the degassing cell, the degassed sample was measured first, followed by opening the degassing cell and connecting it to a balloon containing pure O_2, which saturated the solution with O_2 (aerated). The gray shaded area represents the determined standard deviation of the instrument from ten measurements of the calibration standard.

5. Computational data

5.1. Calculated Raman spectra for *all-E* Cy7

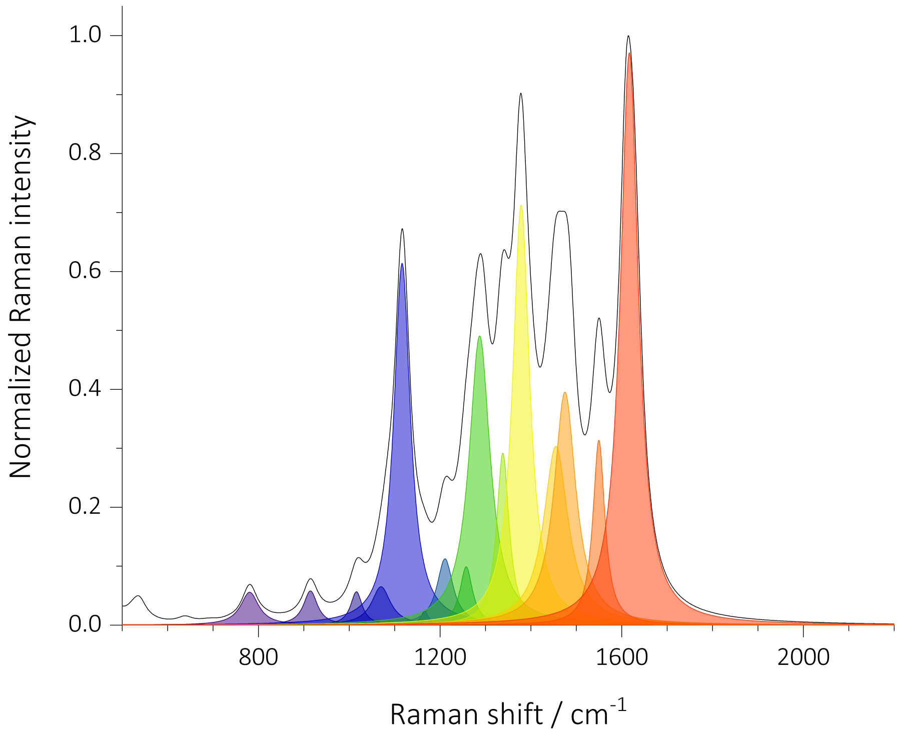

Figure S12. Normalized, calculated Raman spectrum of **all-*E* Cy7** in S_0 state.

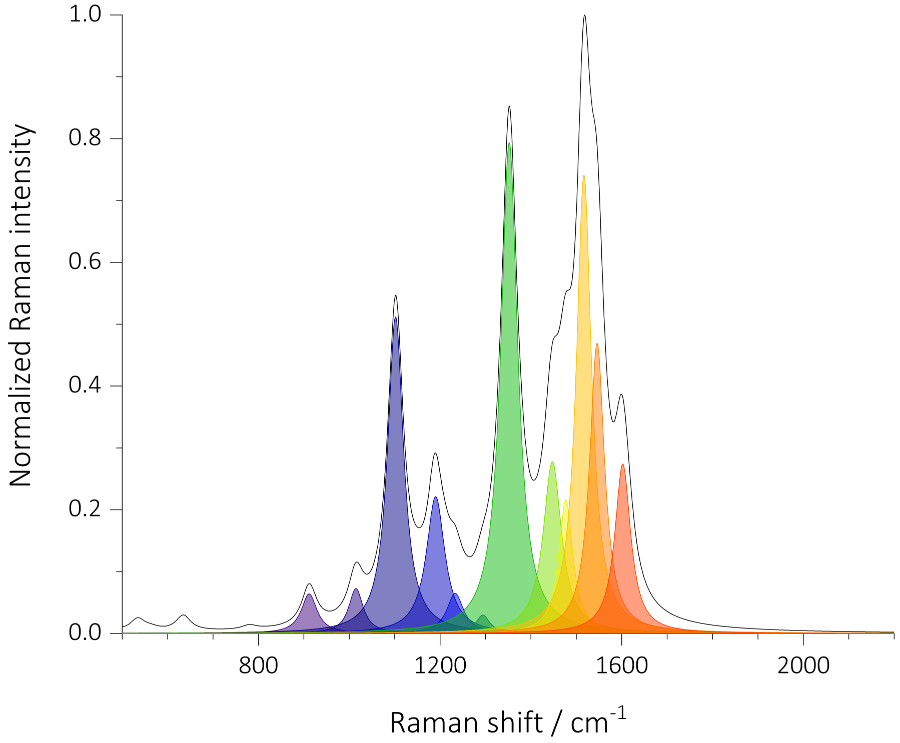

Figure S13. Normalized, calculated Raman spectrum of **all-*E* Cy7** in S_1 state.

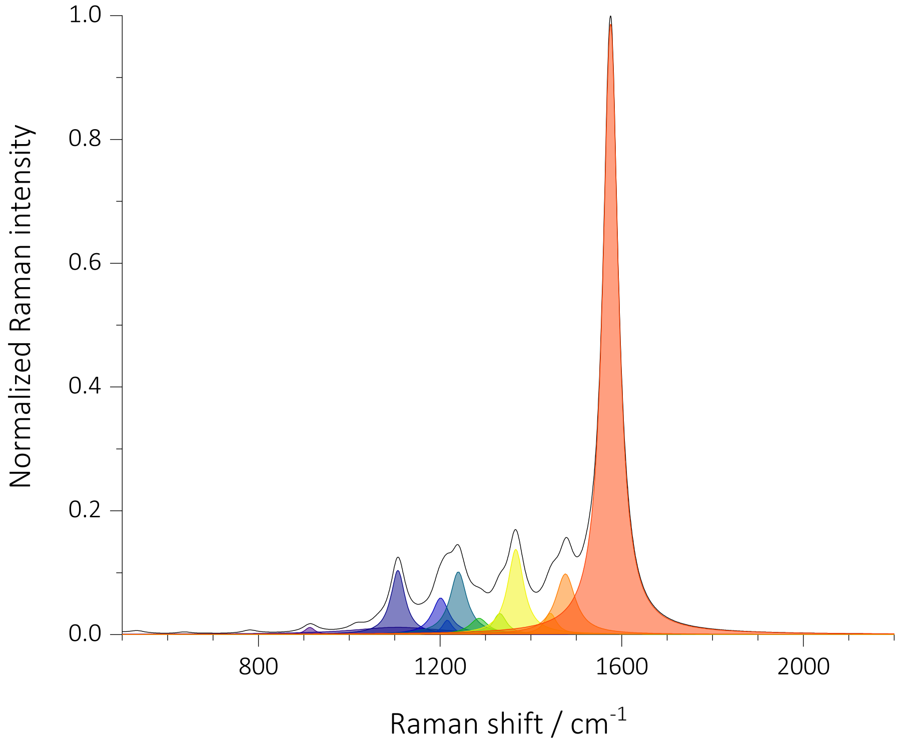

Figure S14. Normalized, calculated Raman spectrum of **all-*E* Cy7** in T_1 state.

5.2. Calculated Raman spectra of Cy7$^{•+}$ with *all-E* Configuration

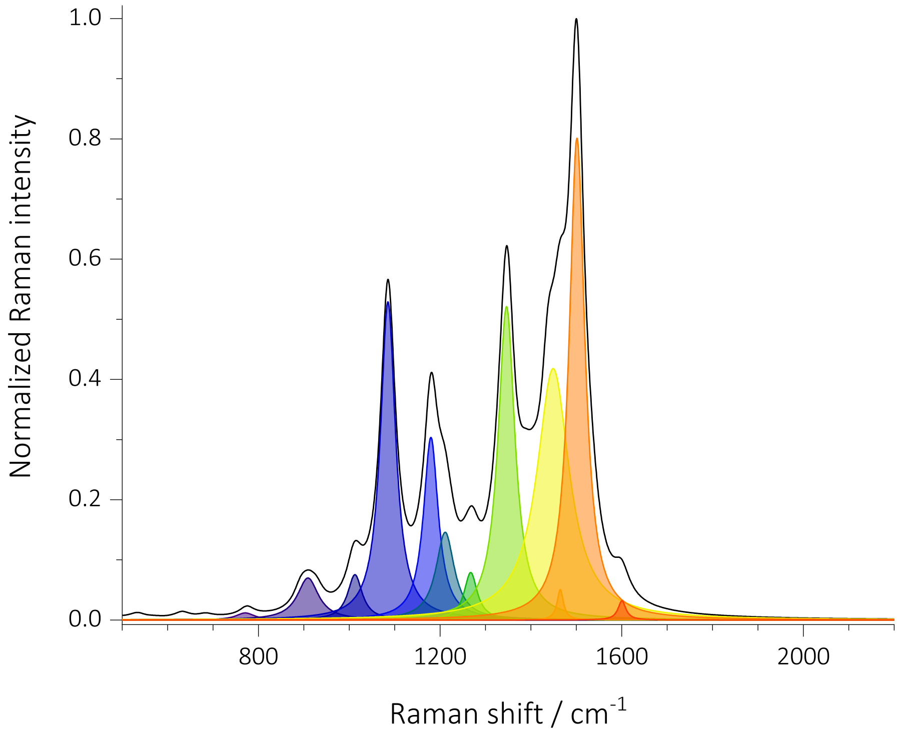

Figure S15. Normalized, calculated Raman spectrum of **Cy7** radical dication (**Cy7$^{•+}$**) in all *E*-conformation.

5.3. Calculated Raman spectra of Z1 – Cy7

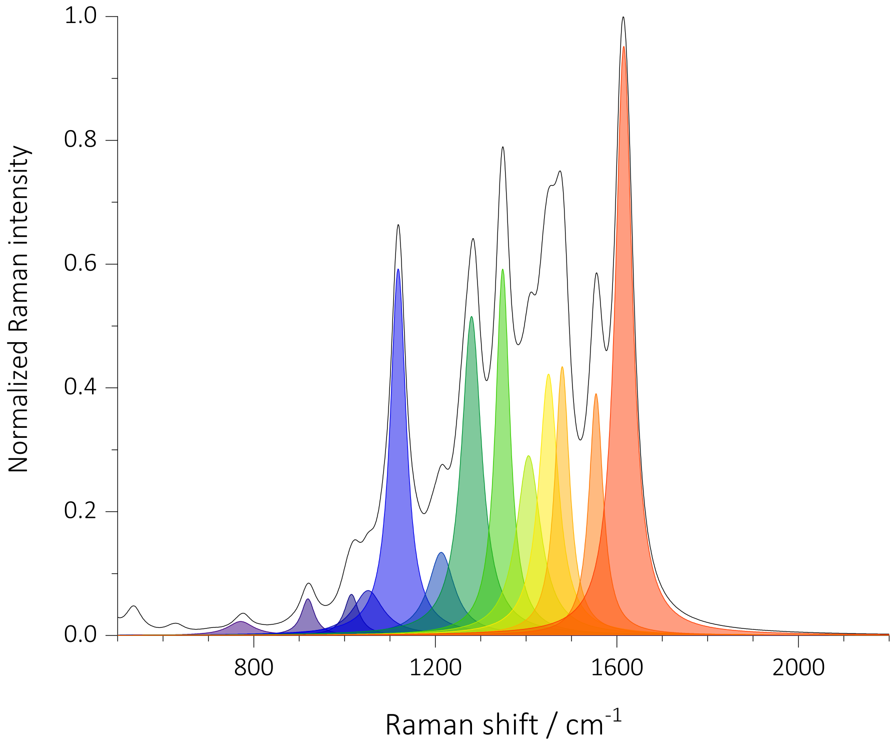

Figure S16. Normalized, calculated Raman spectrum of ***Z1* Cy7** in S_0 state

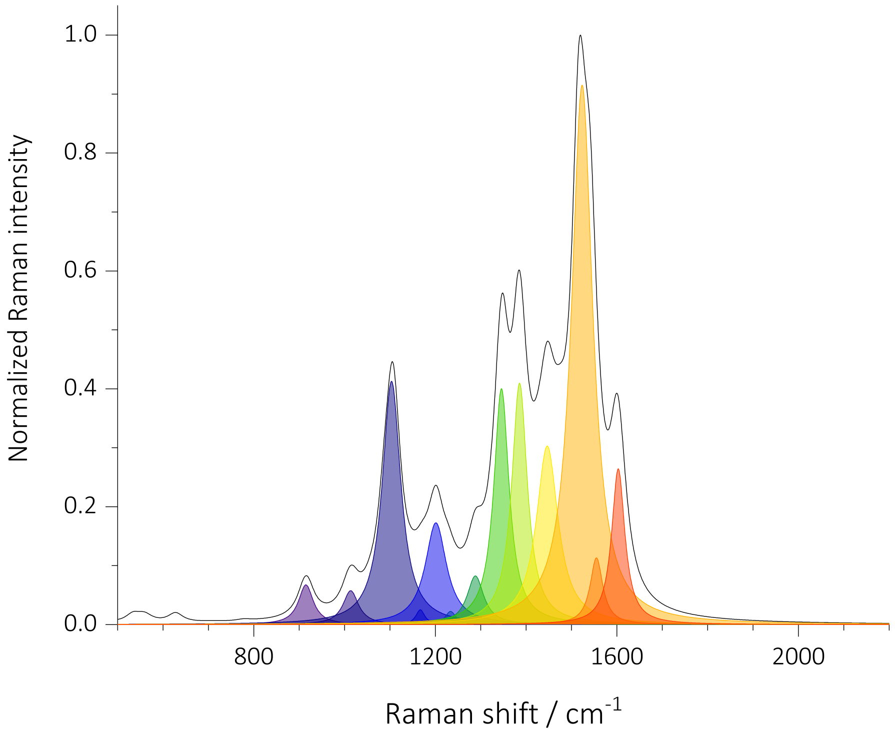

Figure S17. Normalized, calculated Raman spectrum of ***Z1* Cy7** in S_1 state.

5.4. Calculated Raman spectra of Z2 – Cy7

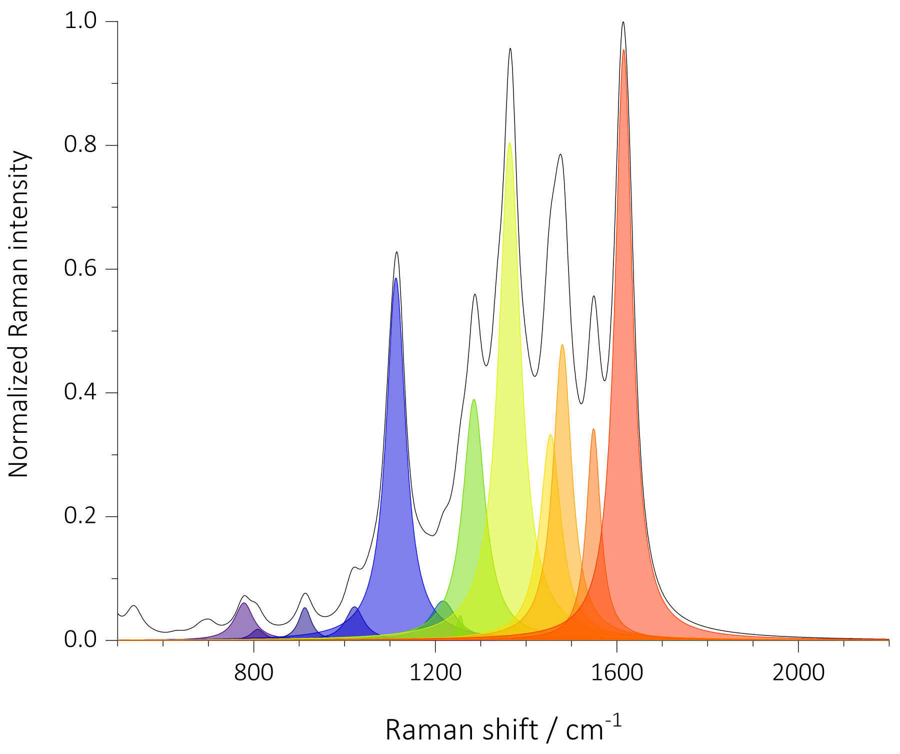

Figure S18. Normalized, calculated Raman spectrum of ***Z2* Cy7** in S_0 state.

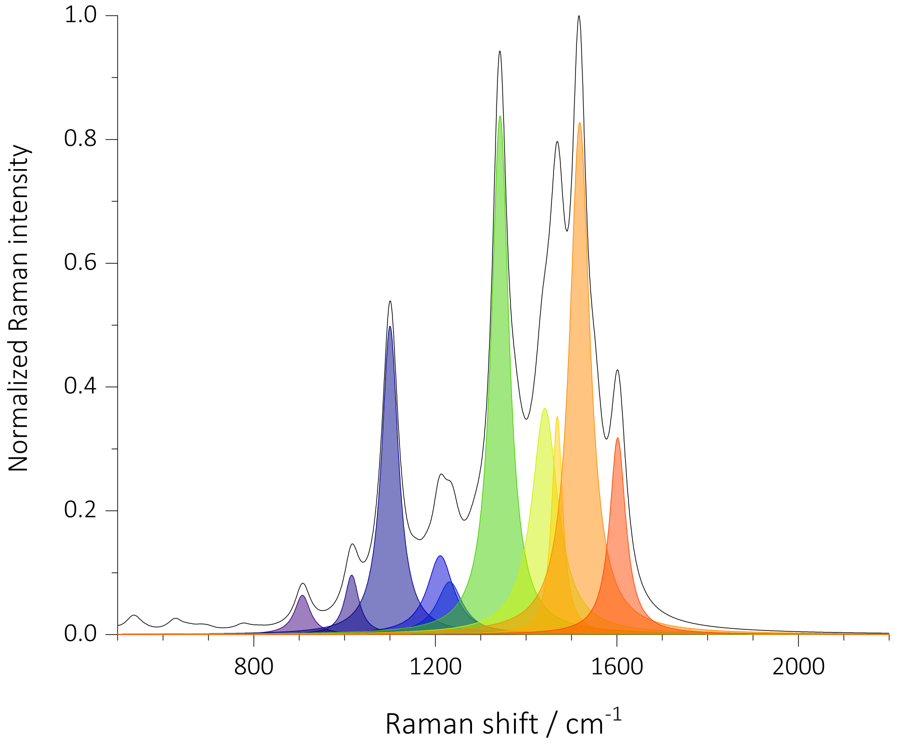

Figure S19. Normalized, calculated Raman spectrum of ***Z2* Cy7** in S_1 state.

5.5. Calculated Raman spectra of Z3 – Cy7

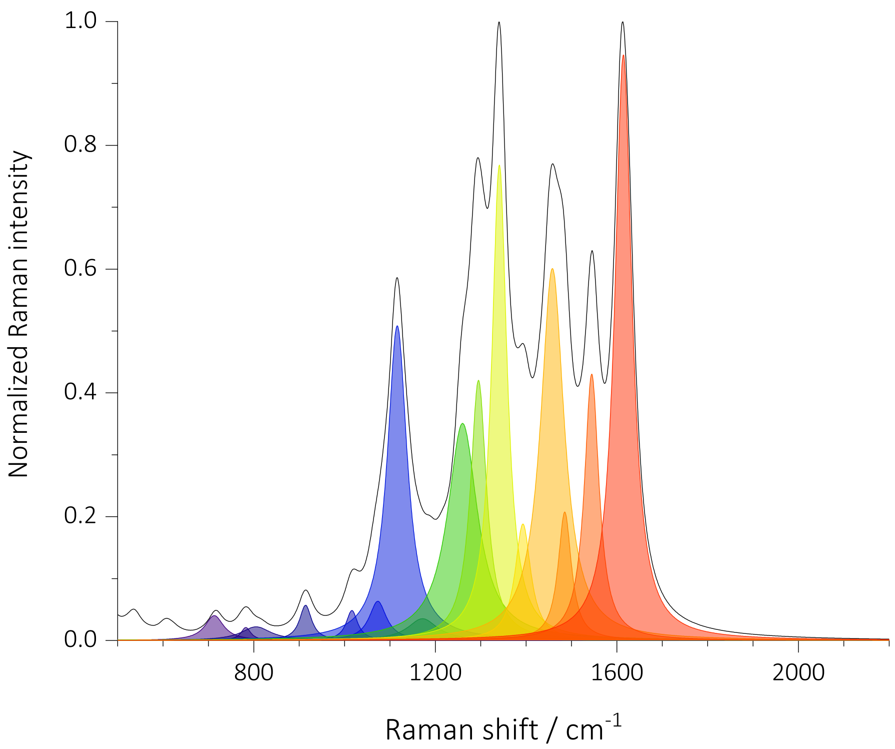

Figure S20. Normalized, calculated Raman spectrum of ***Z3* Cy7** in S_0 state.

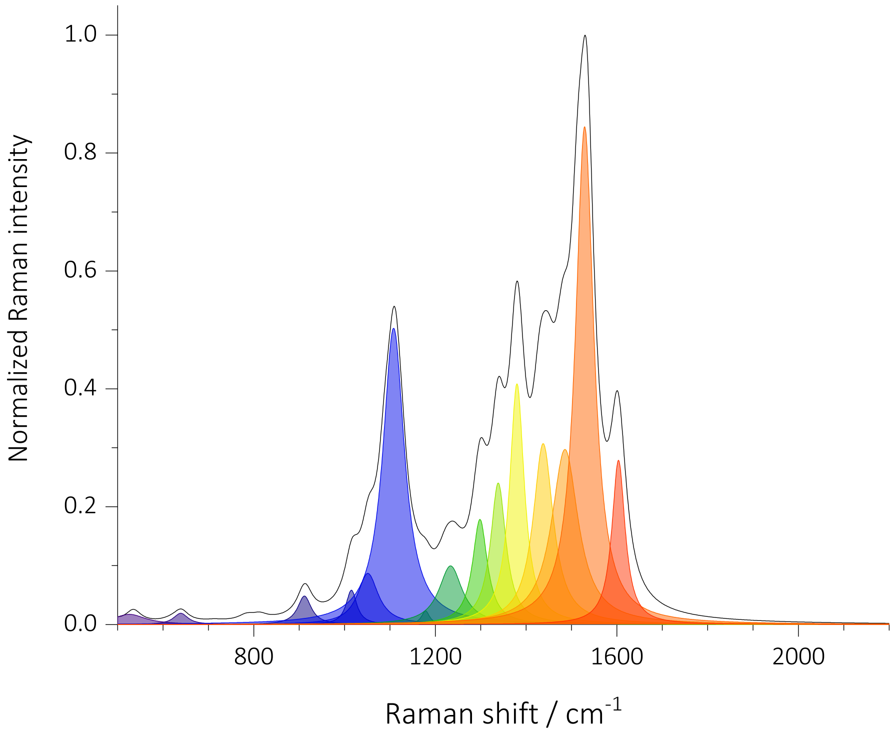

Figure S21. Normalized, calculated Raman spectrum of ***Z3* Cy7** in S_1 state.

5.6. Calculated Raman spectra of Z4 – Cy7

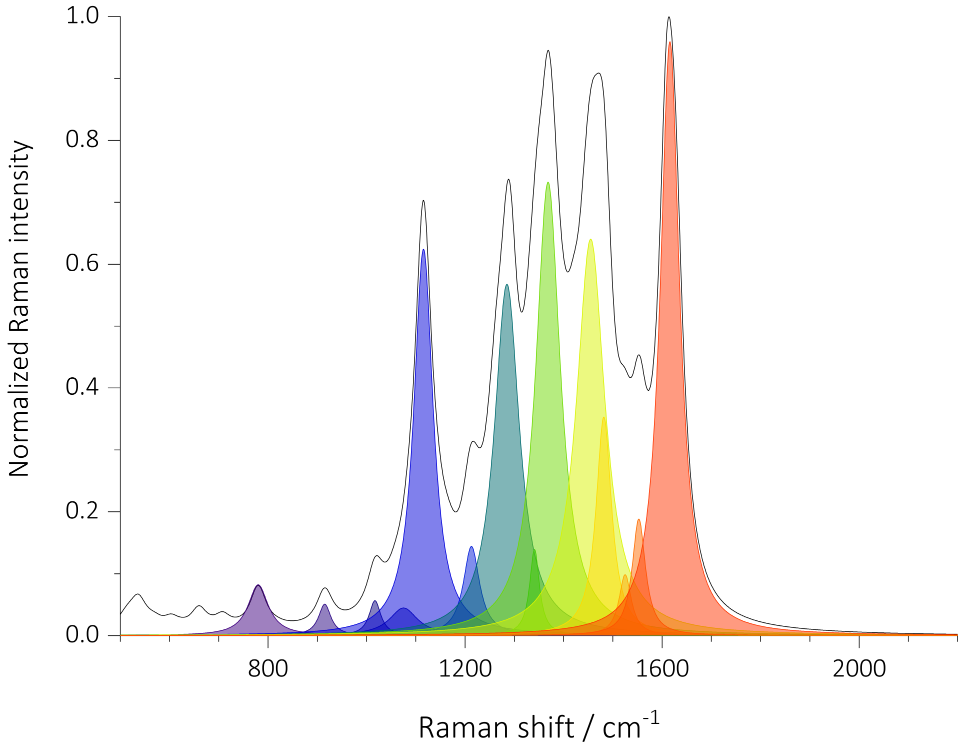

Figure S22. Normalized, calculated Raman spectrum of ***Z4*** **Cy7** in S_0 state.

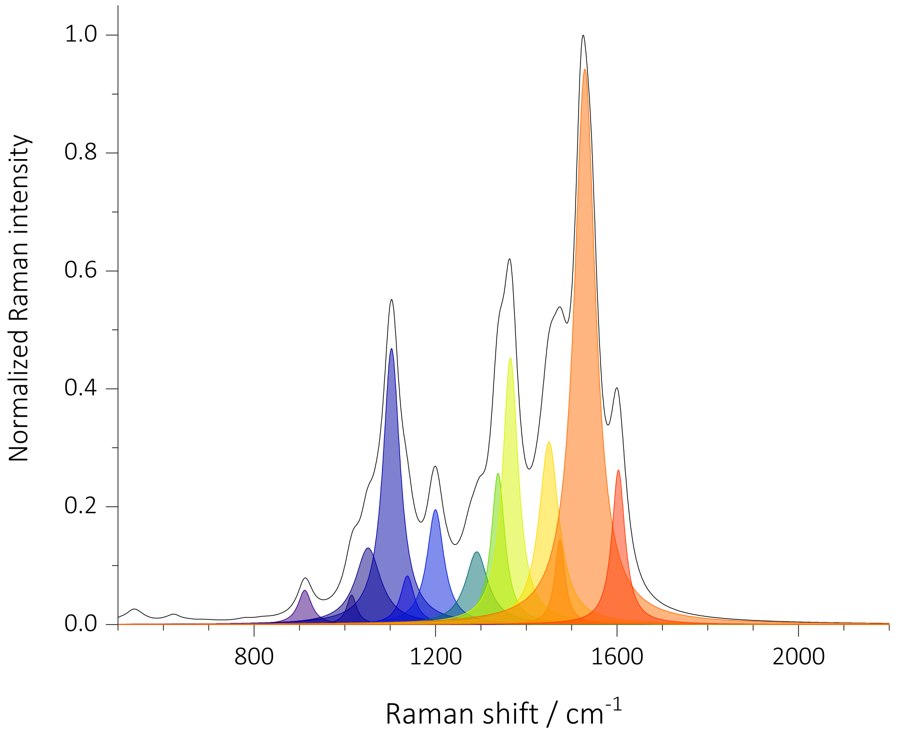

Figure S23. Normalized, calculated Raman spectrum of ***Z4*** **Cy7** in S_1 state.

5.7. Comparison of calculated Raman spectra

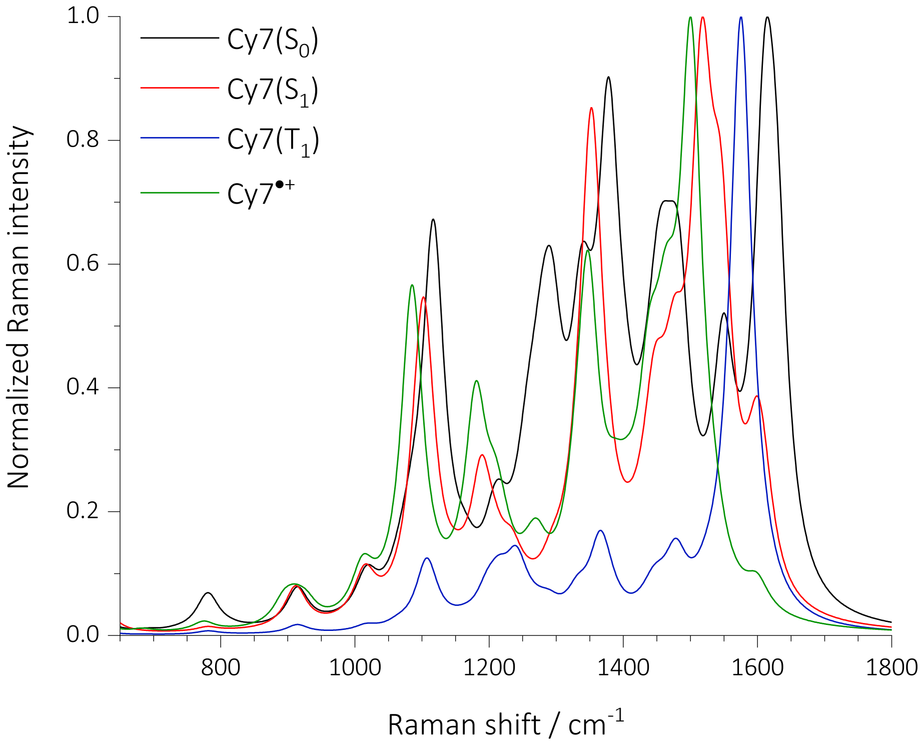

Figure S24. Comparison of normalized, calculated Raman spectra of **all-*E* Cy7**, S_0, S_1, T_1, and **Cy7**$^{\bullet+}$.

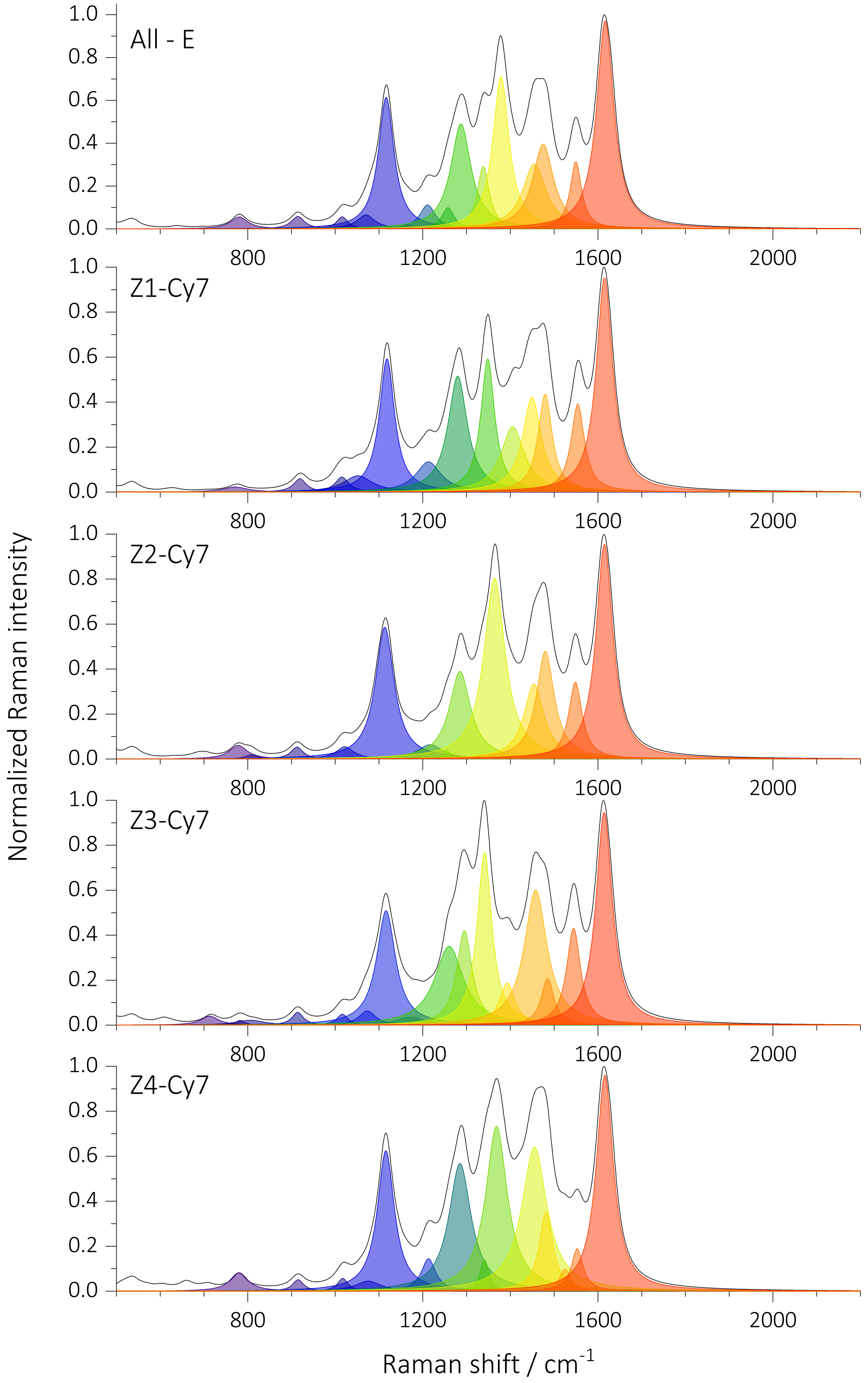

Figure S25. Comparison of normalized, calculated Raman spectra of ***all-E*** **Cy7** (S_0) and **Z1–Z4 Cy7** in S_0 states.

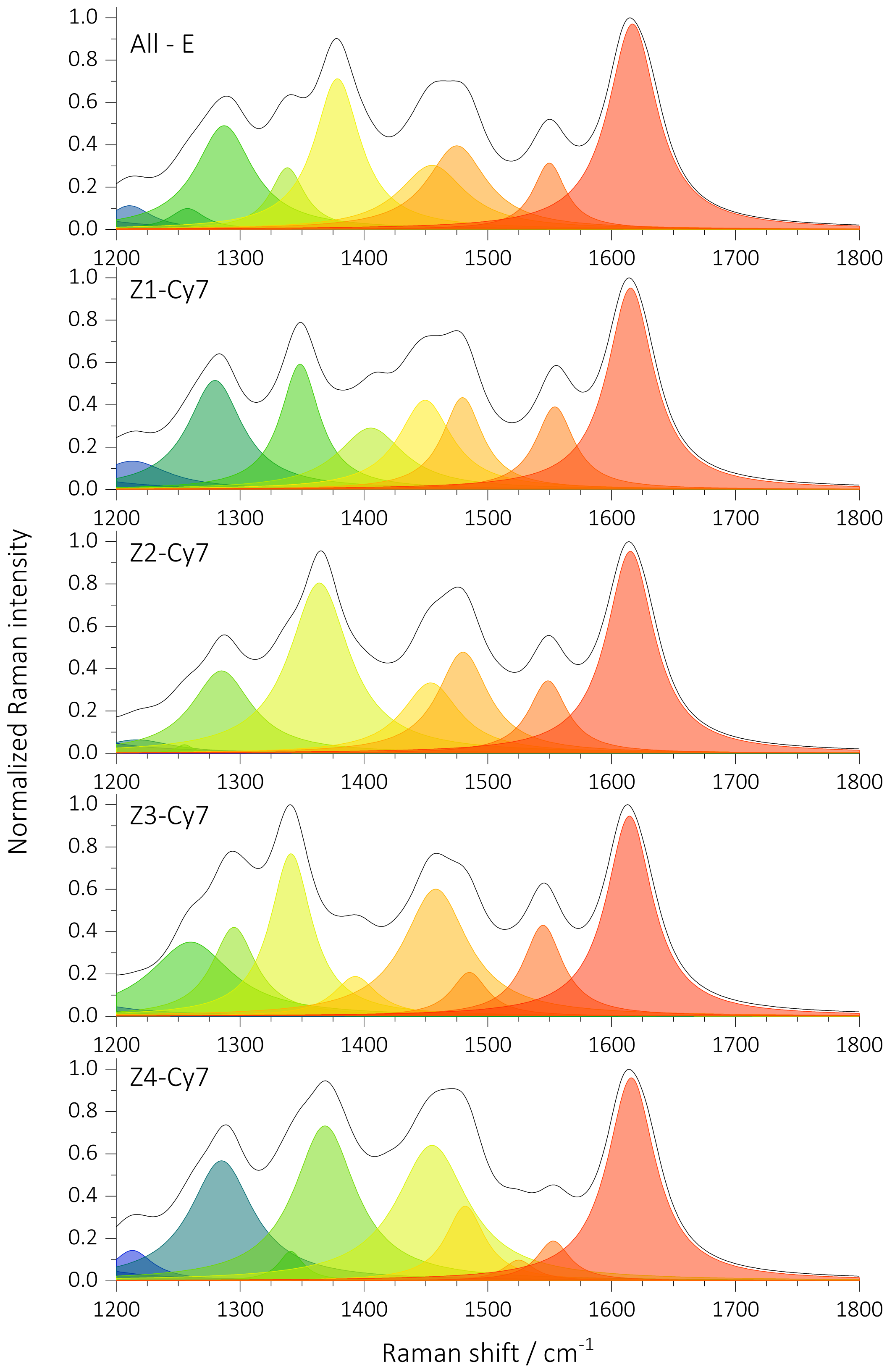

Figure S26. Comparison of normalized, calculated Raman spectra of ***all-E*** **Cy7** (S_0) and **Z1-Z4 – Cy7** in S_0 states (zoom at the 1200 – 1800 cm^{-1} region).

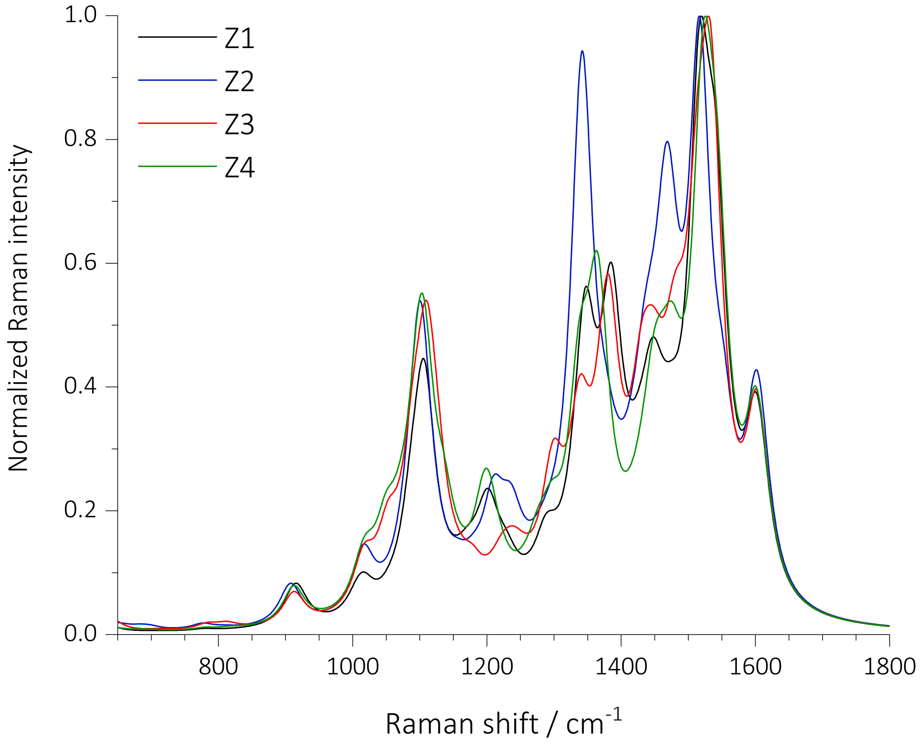

Figure S27. Comparison of normalized, calculated Raman spectra of **Z1–Z4 Cy7** S_1 states.

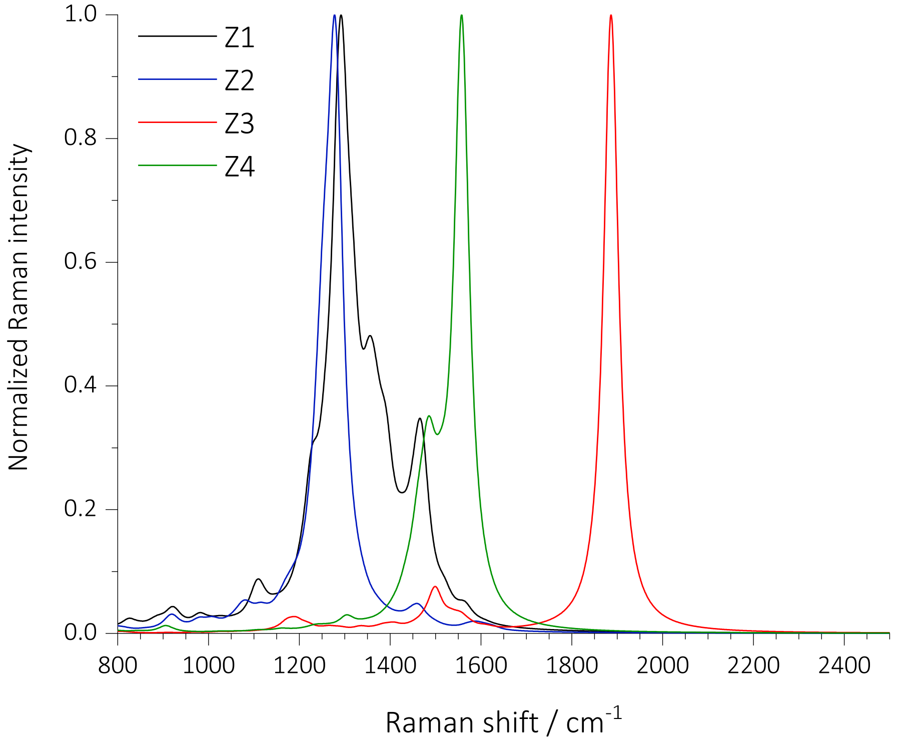

Figure S28. Comparison of normalized, calculated Raman spectra of **Z1–Z4 Cy7** twisted states.

5.8. Interpretation of calculated Raman spectra

Table S1. Calculated Raman shifts in cm^{-1} with the most significant intensities and the corresponding vibrational motions for **all-*E***, ***Z*1**, ***Z*2**, ***Z*3** and ***Z*4** structures in the ground state. Most of the molecular motions are delocalized. In the first column, we show typically the most dominant motion between the two. Note on the shifts in parentheses: The original vibration attributed to both end groups simultaneously for the **all-*E*** structure (due to molecular symmetry) splits upon isomerization into two separate vibrational modes each contributing to the same spectral band. The shifts marked by asterisk (*) exhibit significantly lower intensity than their counterparts for other isomers in a row.

Entry	Motion 1 (Dominant)	Motion 2	all-*E*	*Z*1	*Z*2	*Z*3	*Z*4
1	C–C stretching in aromatic rings	C=C stretching in polyene chain	1628	1625	1626	1625	1627
2	C–C stretching in aromatic rings	C=C stretching in polyene chain	1610	1611<br>(1603)	1611<br>(1607)	1611<br>(1609)	1610<br>(1610)
3	C=C stretching in polyene chain	Symmetric bending of H atoms in –N–CH_3 groups	1549	1554	1548	1545	1553
4	C–C stretching between aromatic rings	C–H stretching in –N–CH_3	1483	1480	1484<br>(1481)	1483	1483
5	Scissor bending of H atoms in all CH_3	Scissor bending of H atoms on aromatic rings	1451	1448	1474<br>(1450)	1463*	-
6	Scissor bending of H atoms in –N–CH_3 groups	Asymmetric bending of H atoms on aromatic rings	-	-	-	-	1473
7	Scissor bending of H atoms in CH_3 groups	Asymmetric bending of H atoms on aromatic rings	-	-	-	1451	1451
8	Scissor bending of H atoms in CH_3 groups	C–N stretching in heterocycle	-	-	-	-	1382
9	Symmetric bending of H atoms in CH_3 groups	Symmetric bending of H atoms on polyene chain	1376	1436<br>(1409)	1365	1393	1368
10	Symmetric bending of H atoms on aromatic rings	Symmetric bending of H atoms on polyene chain	1336	1348	1336	-	-
11	Symmetric bending of H atoms on polyene chain	Symmetric bending of H atoms on aromatic rings	1293	-	-	1342<br>(1337)	1347<br>(1334)
12	Symmetric bending of H atoms on polyene chain	Asymmetric bending of H atoms on aromatic rings	1278	1284	1286	1290<br>(1286)	1290
13	Symmetric bending of H atoms on polyene chain	C–C stretch on aromatic rings	-	1264	1255*	-	1264*
14	Bending of H atoms + C=C stretch on polyene chain	Symmetric bending of H atoms on aromatic rings	1207	1211	1216*	-	1209
15	Symmetric bending of H atoms in double-CH_3	Symmetric bending of H atoms on aromatic rings	1164	-	-	-	-
16	Asymmetric bending of H atoms in –N–CH_3 groups	Symmetric bending of H atoms on aromatic rings	1117	1117	1118<br>(1113)	1117<br>(1110)	1116
17	C=C stretching in polyene chain	Asymmetric bending of H atoms in –N–CH_3 groups	1083	1086	1098	-	1086*
18	C–C stretching of outer carbon atoms in aromatic rings	Symmetric bending of H atoms on aromatic rings	1017	1021	1016	1017<br>(1014)	1018
19	H bending in double-CH_3	H bending in –N–CH_3 groups	914	921	913	914<br>(910)	914

Table S2. Calculated Raman shifts (in cm^{-1}) with the most significant intensities and the corresponding vibrational motions for **all-*E* Cy7** in the S_1 and T_1 states and **Cy$^{\bullet+}$** state. Most of the molecular motions are delocalized. In the first column, we show typically the most dominant motion between the two.

Entry	Motion 1 (Dominant)	Motion 2	**all-*E*** (S_1)	**all-*E*** (T_1)	**Cy$^{\bullet+}$** (D_0)
1	C–C stretching in aromatic rings	-	1611	-	-
2	C–C stretching in aromatic rings	-	1599	-	1598
3	C–C stretching in aromatic rings	C=C stretching in polyene chain	-	1603	-
4	C=C stretching in polyene chain	-	1545	1575	1532
5	C=C stretching in polyene chain	Symmetric bending of H atoms in –N–CH_3 groups	1516	1524	-
6	C–C stretching between aromatic rings	C–H stretching in –N–CH_3	1476	-	-
7	Scissor bending of H atoms in all CH_3	Scissor bending of H atoms on aromatic rings	1456	1478	-
8	Asymmetric bending of H atoms on aromatic rings	Scissor bending of H atoms in CH_3 groups	-	-	1467
9	Scissor bending of H atoms in –N–CH_3 groups	-	-	-	1462
10	Asymmetric bending of H atoms on aromatic rings	-	-	-	1436
11	Scissor bending of H atoms in –N–CH_3 groups	Asymmetric bending of H atoms on aromatic rings	1446	1430	-
12	Scissor bending of H atoms in CH_3 groups	Symmetric –N–CH_3 bending	-	1376	-
13	Symmetric bending of H atoms on polyene chain	Symmetric –N–CH_3 bending	1352	1365	-
14	Symmetric bending of H atoms in CH_3 groups	Symmetric bending of H atoms on polyene chain	-	1331	1388
15	Symmetric bending of H atoms on aromatic rings	Symmetric bending of H atoms on polyene chain	-	1289	-
16	Symmetric bending of H atoms on aromatic rings	Symmetric bending of H atoms on polyene chain	-	1240	-
17	Symmetric bending of H atoms on polyene chain	C=C stretching in polyene chain	-	-	1347
18	Symmetric bending of H atoms on polyene chain	Asymmetric bending of H atoms on aromatic rings	1233	1213	1269
19	Symmetric bending of H atoms on polyene chain	Asymmetric bending of H atoms on aromatic rings	1188	1196	1208
20	Symmetric bending of H atoms on polyene chain	-	-	-	1179
21	Bending of H atoms + C=C stretch on polyene chain	Symmetric bending of H atoms on aromatic rings	-	1106	-
22	Asymmetric bending of H atoms in –N–CH_3 groups	Symmetric bending of H atoms on aromatic rings	1102	-	1084
23	C–C stretching of outer carbon atoms in aromatic rings	Symmetric bending of H atoms on aromatic rings	1014	1016	1010
24	Asymmetric bending of H atoms on polyene chain	-	-	-	927
25	H bending in double-CH_3	H bending in –N–CH_3 groups	911	913	909

5.9. Calculated potential energy surfaces

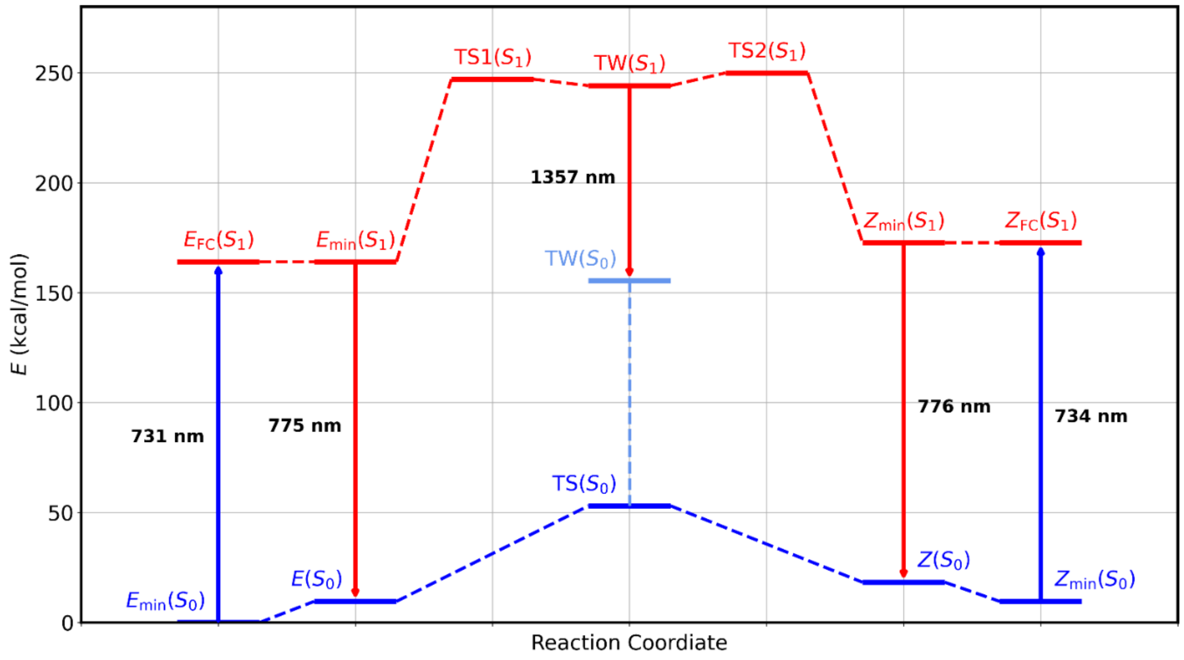

Figure S29. Calculated energy profile for isomerization around the C=C bond to get **Z1 Cy7** in the S_0 and S_1 states. The energy calculations were conducted at the CIS(D)/TZVP level of theory using the ωB97X-D/6-31G* optimized geometries.

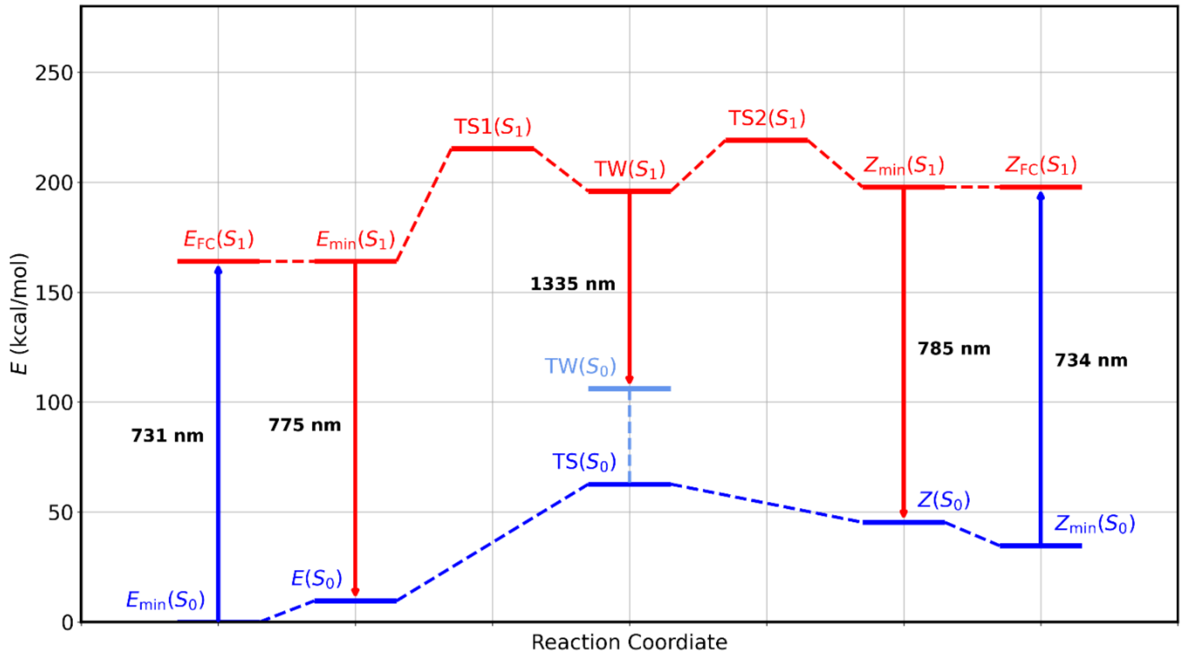

Figure S30. Calculated energy profile for isomerization around the formal C=C bond to get **Z2 Cy7** in the S_0 and S_1 states. The energy calculations were conducted at the CIS(D)/TZVP level of theory using the ωB97X-D/6-31G* optimized geometries.

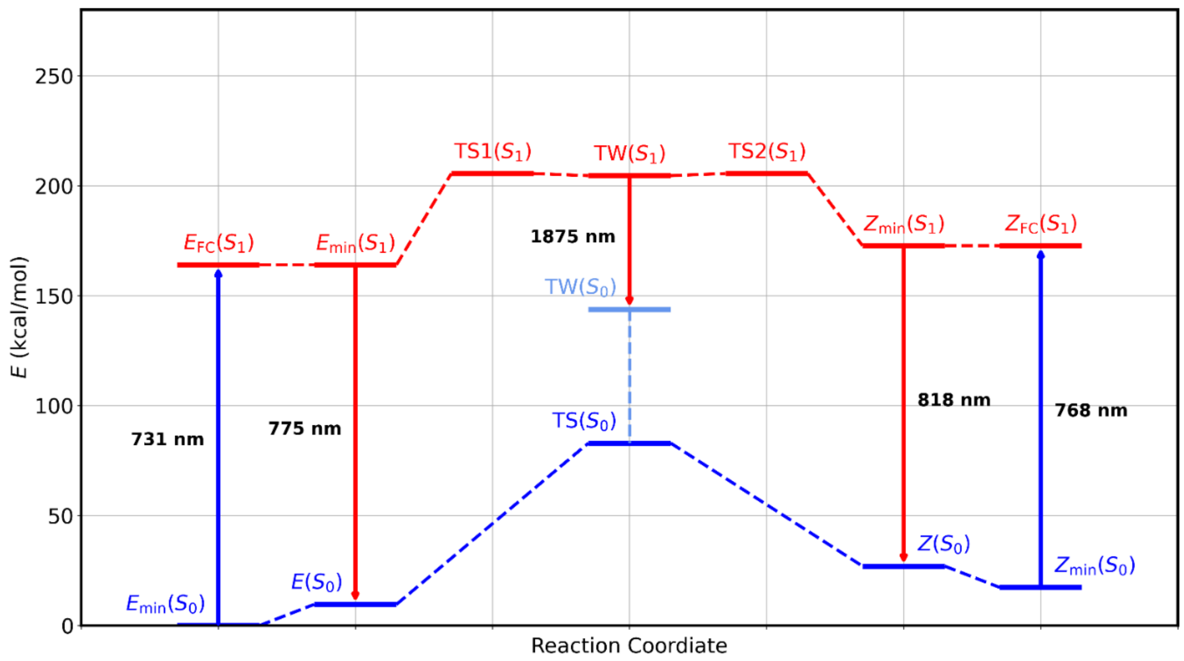

Figure S31. Calculated energy profile for isomerization around the formal C=C bond to get **Z3 Cy7** in the S_0 and S_1 states. The energy calculations were conducted at the CIS(D)/TZVP level of theory using ωB97X-D/6-31G* optimized geometries.

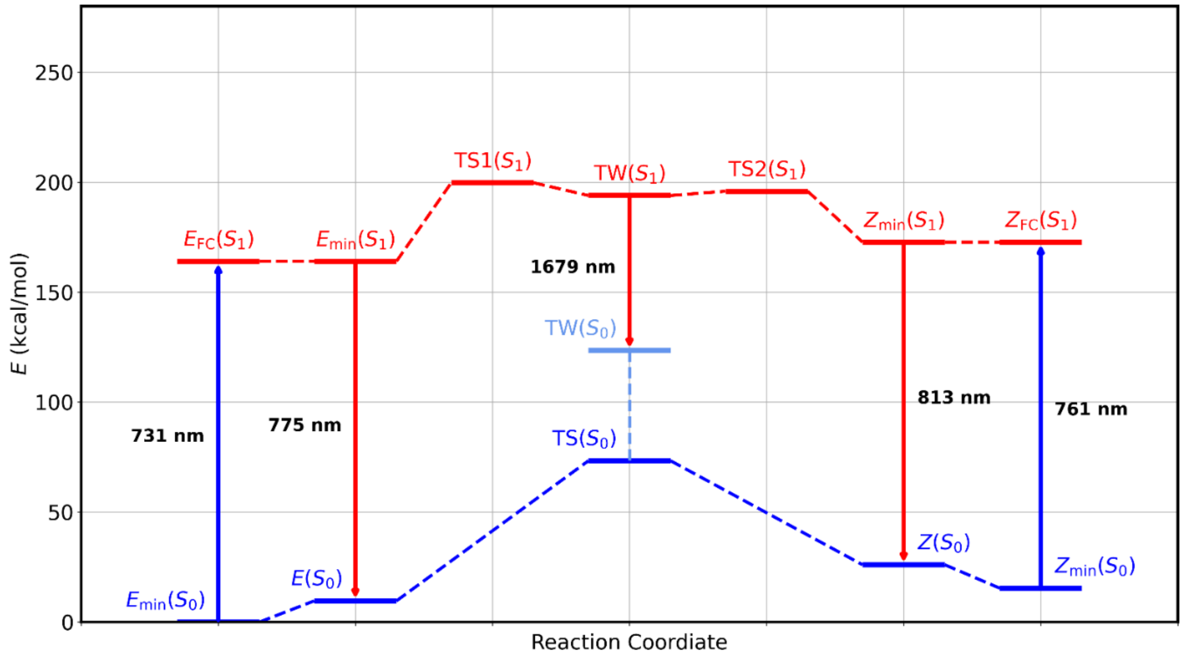

Figure S32. Calculated energy profile for isomerization around the formal C=C bond to get **Z4 Cy7** in the S_0 and S_1 states. The energy calculations were conducted at the CIS(D)/TZVP level of theory using the ωB97X-D/6-31G* optimized geometries.

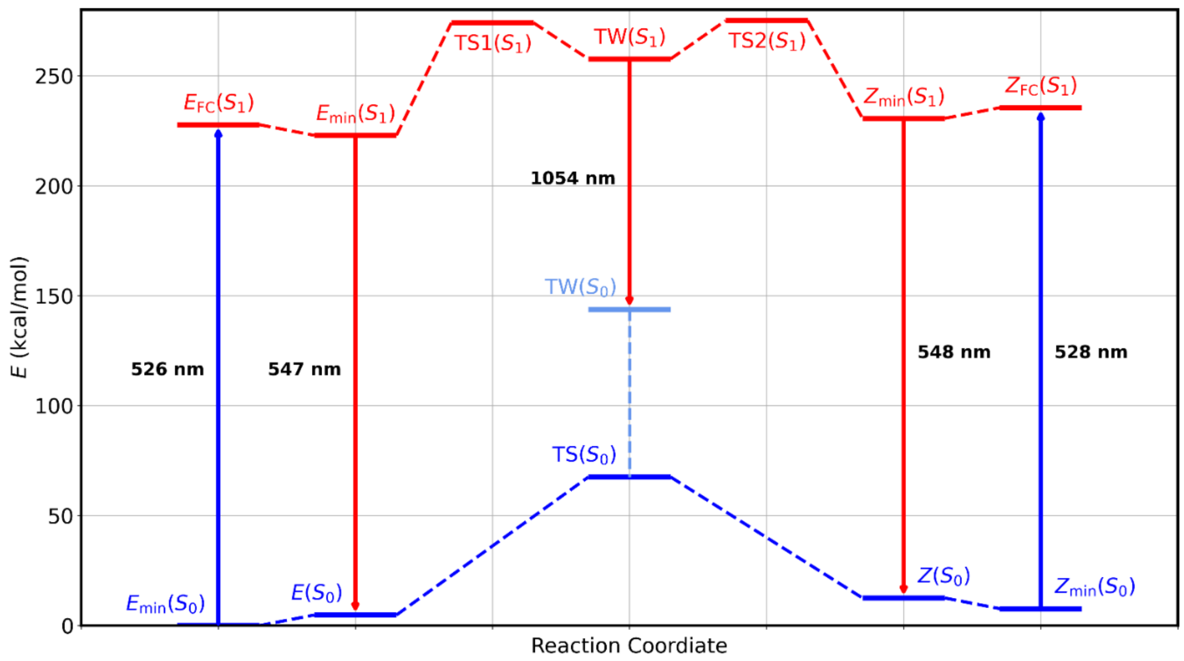

Figure S33. Calculated energy profile for isomerization around the formal C=C bond to get **Z1 Cy7** in the S_0 and S_1 states. The energy calculations were conducted at the TD-ωB97X-D/6-31G* level of theory using ωB97X-D/6-31G* optimized geometries.

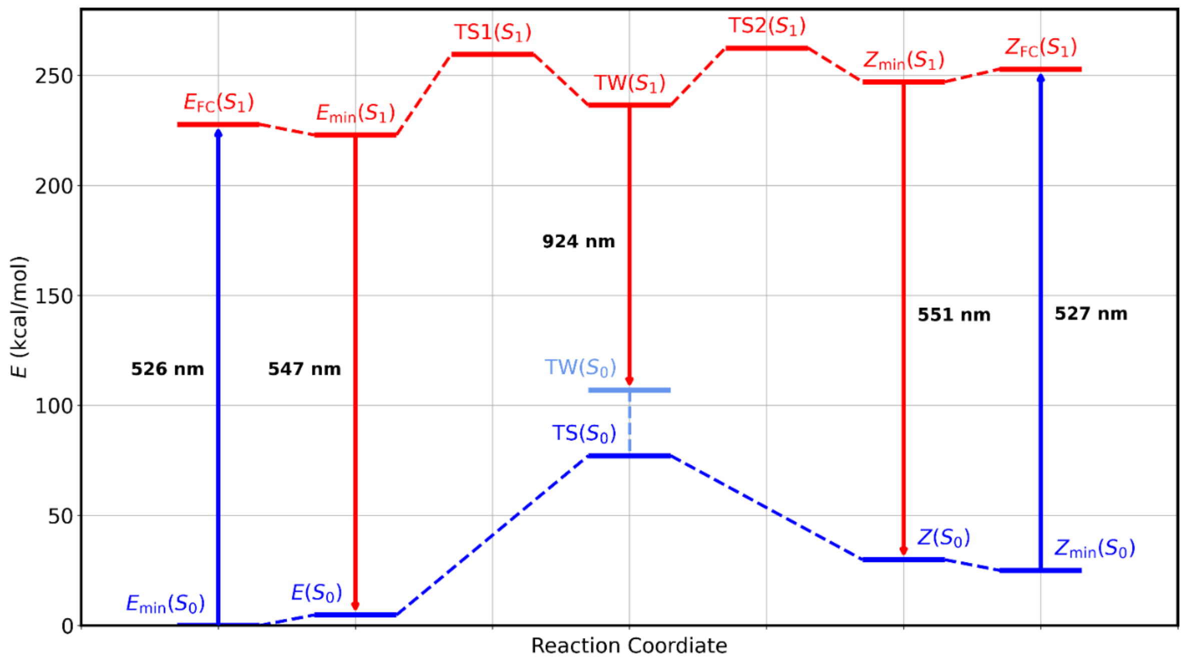

Figure S34. Calculated energy profile for isomerization around the formal C=C bond to get **Z2 Cy7** in the S_0 and S_1 states. The energy calculations were conducted at the TD-ωB97X-D/6-31G* level of theory using the ωB97X-D/6-31G* optimized geometries.

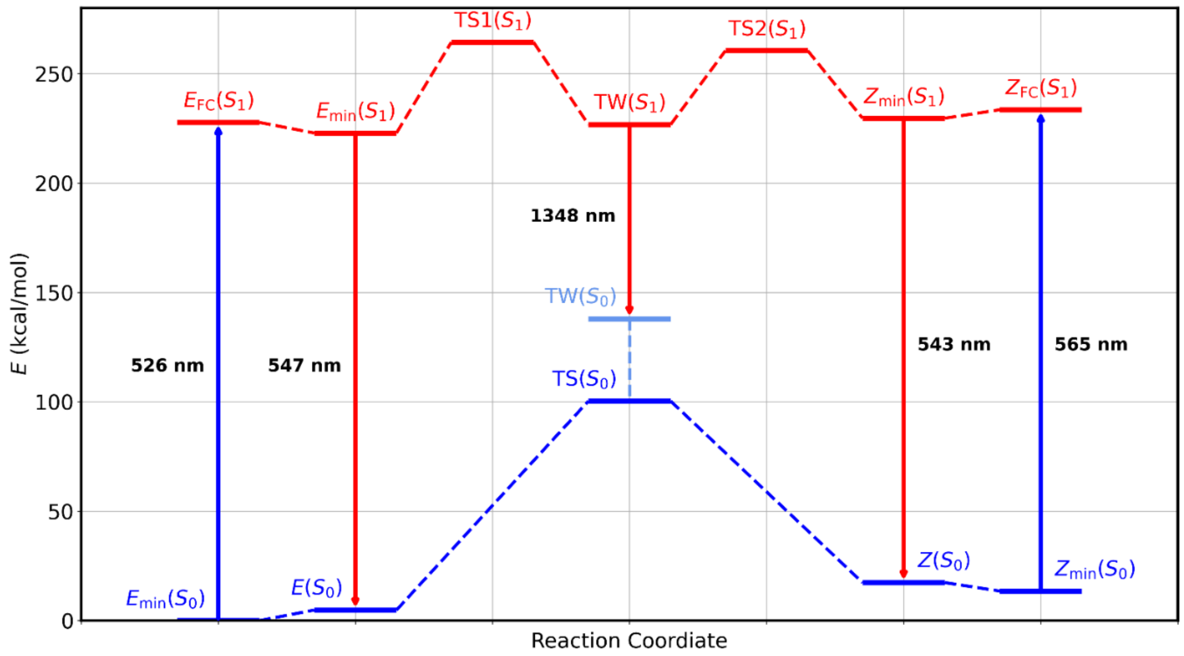

Figure S35. Calculated energy profile for isomerization around the formal C=C bond to get **Z3 Cy7** in the S_0 and S_1 states. The energy calculations were conducted at the TD-*ω*B97X-D/6-31G* level of theory using the *ω*B97X-D/6-31G* optimized geometries.

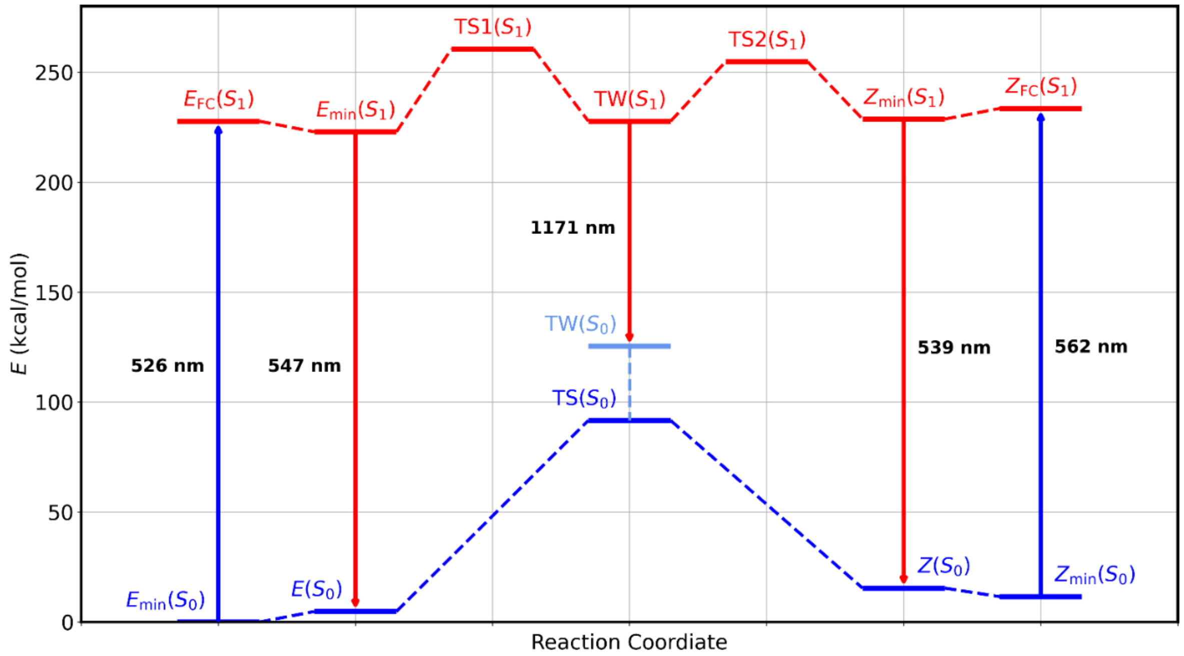

Figure S36. Calculated energy profile for isomerization around the formal C=C bond to get **Z4 Cy7** in the S_0 and S_1 states. The energy calculations were conducted at the TD-*ω*B97X-D/6-31G* level of theory using the *ω*B97X-D/6-31G* optimized geometries.

6. FSR spectra

6.1. Experimental FSR spectra of Cy7 in acetonitrile

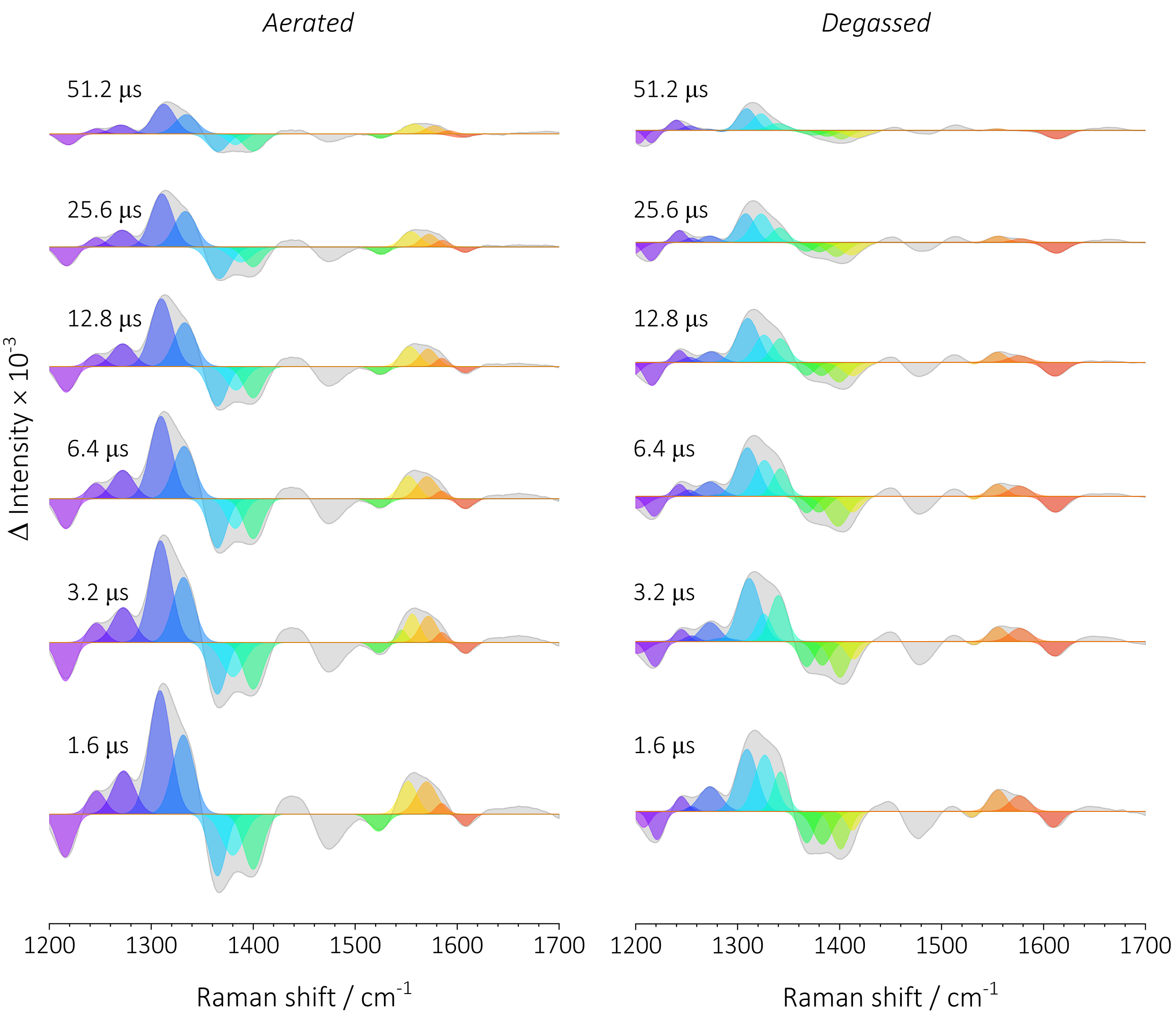

Figure S37. Deconvoluted selected experimental FSR spectra of **Cy7** in acetonitrile under aerated conditions (left side) and degassed conditions (freeze-pump-thaw, right side) at the µs time delays (the delay is indicated above each spectrum). All spectra are on the same vertical scale.

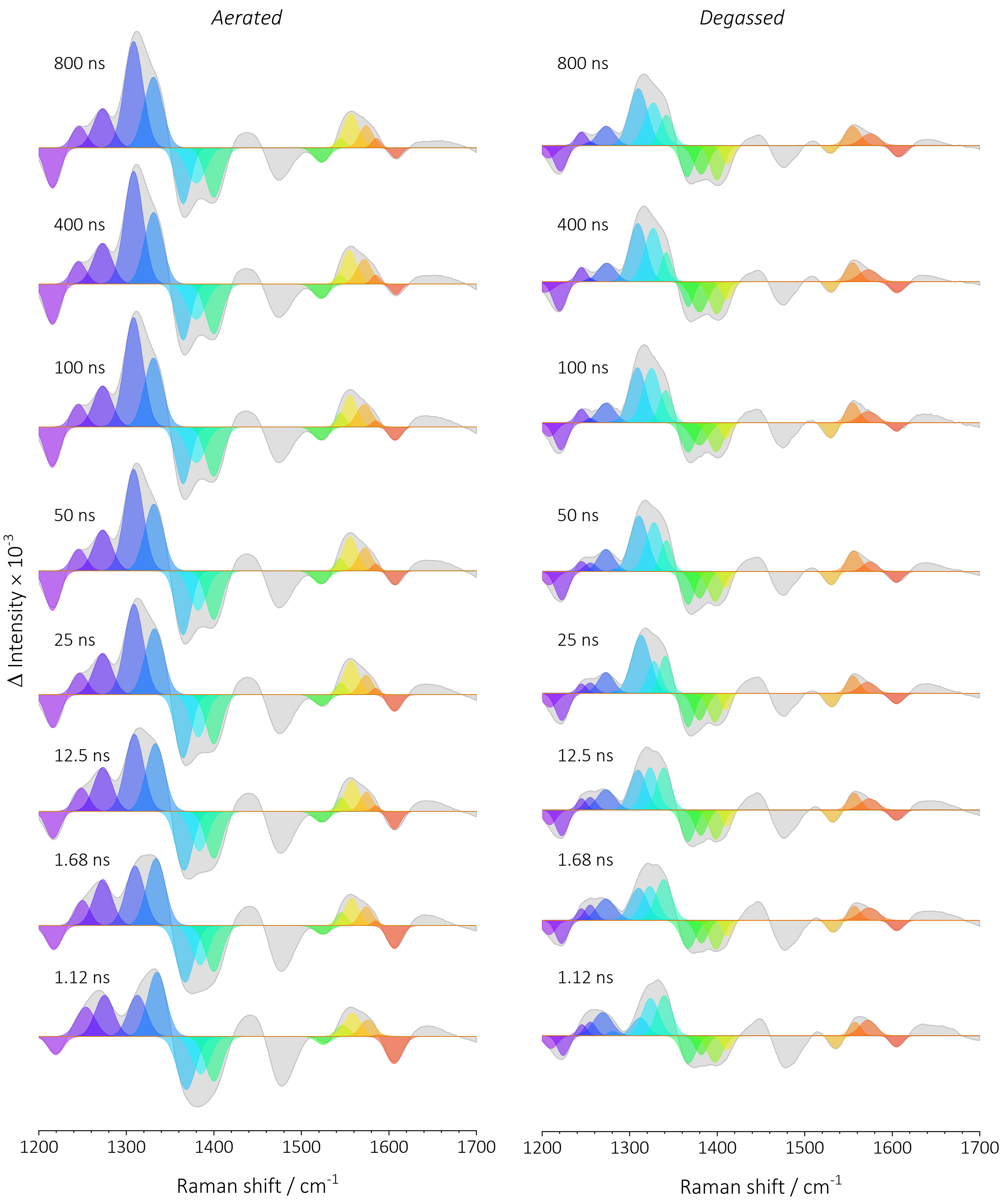

Figure S38. Deconvoluted selected experimental FSR spectra of **Cy7** in acetonitrile under aerated conditions (left side) and degassed conditions (freeze-pump-thaw, right side) at the ns time delays (the delay indicated above each spectrum). All spectra are on the same vertical scale.

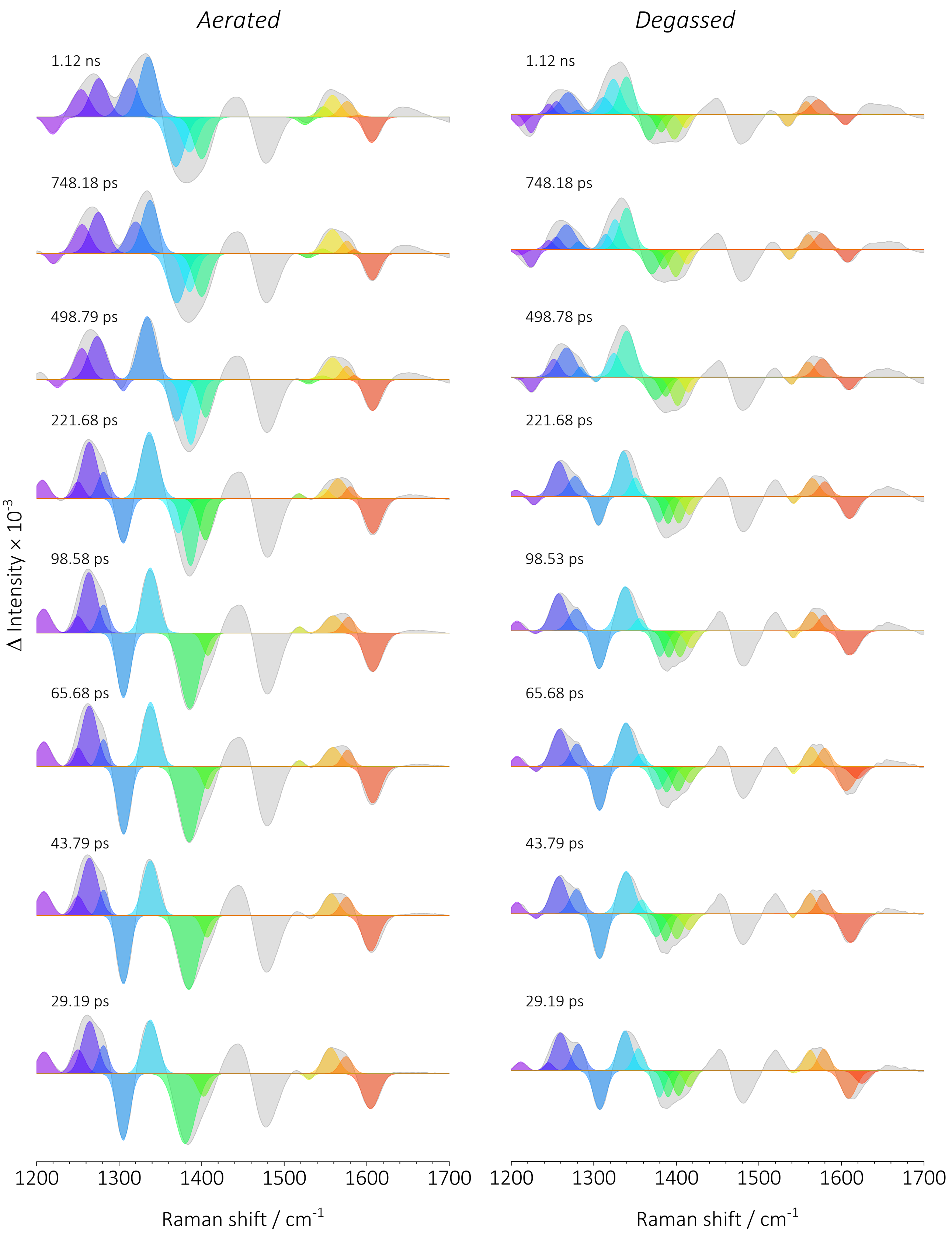

Figure S39. Deconvoluted selected experimental FSR spectra of **Cy7** in acetonitrile under aerated conditions (left side) and degassed conditions (freeze-pump-thaw, right side) at the 1.12 ns–29.19 ps time delays (the delay indicated above each spectrum). All spectra are on the same vertical scale.

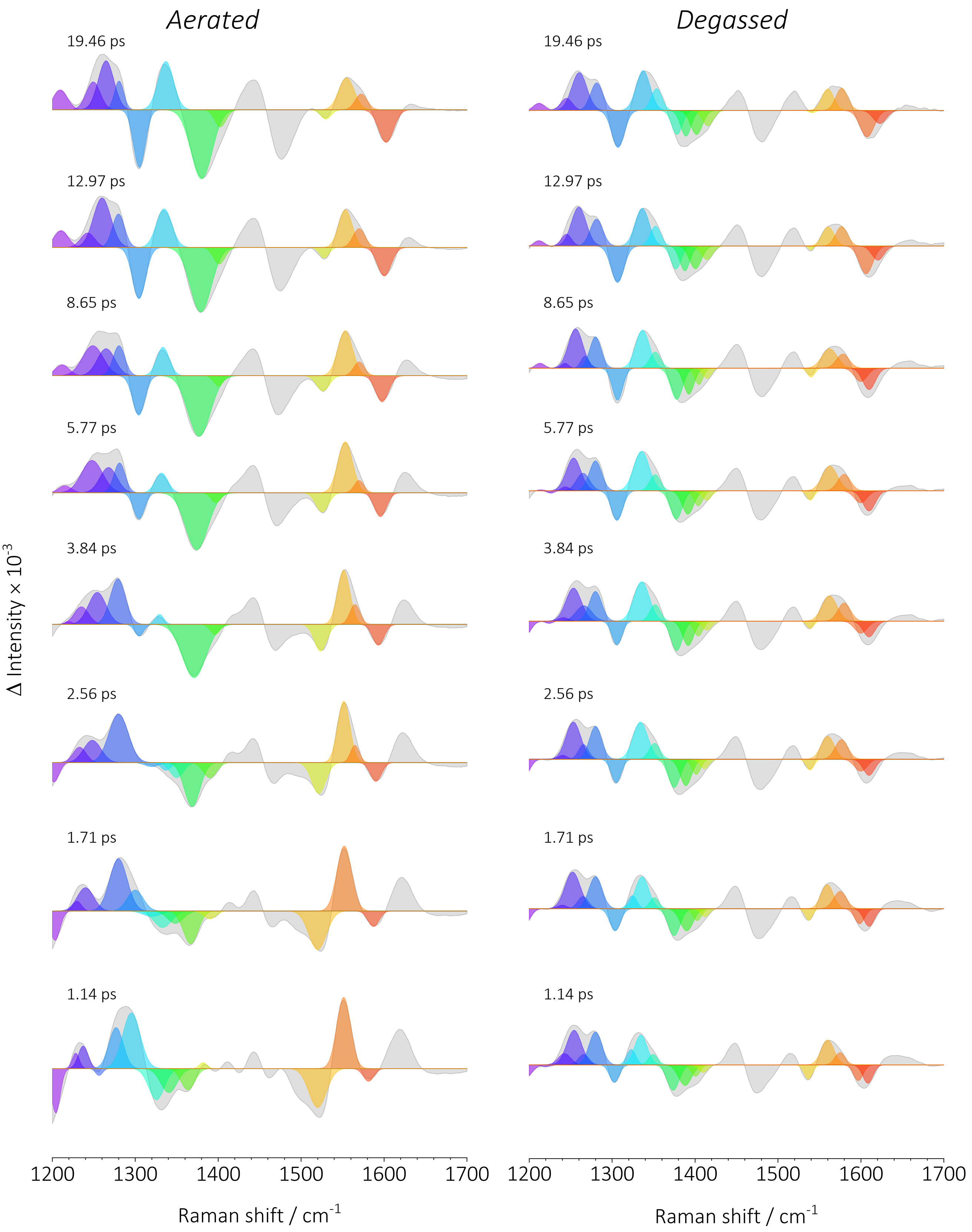

Figure S40. Deconvoluted selected experimental FSR spectra of **Cy7** in acetonitrile under aerated conditions (left side) and degassed conditions (freeze-pump-thaw, right side) at the 19.46–1.14 ps time delays (the delay indicated above each spectrum). All spectra are on the same vertical scale.

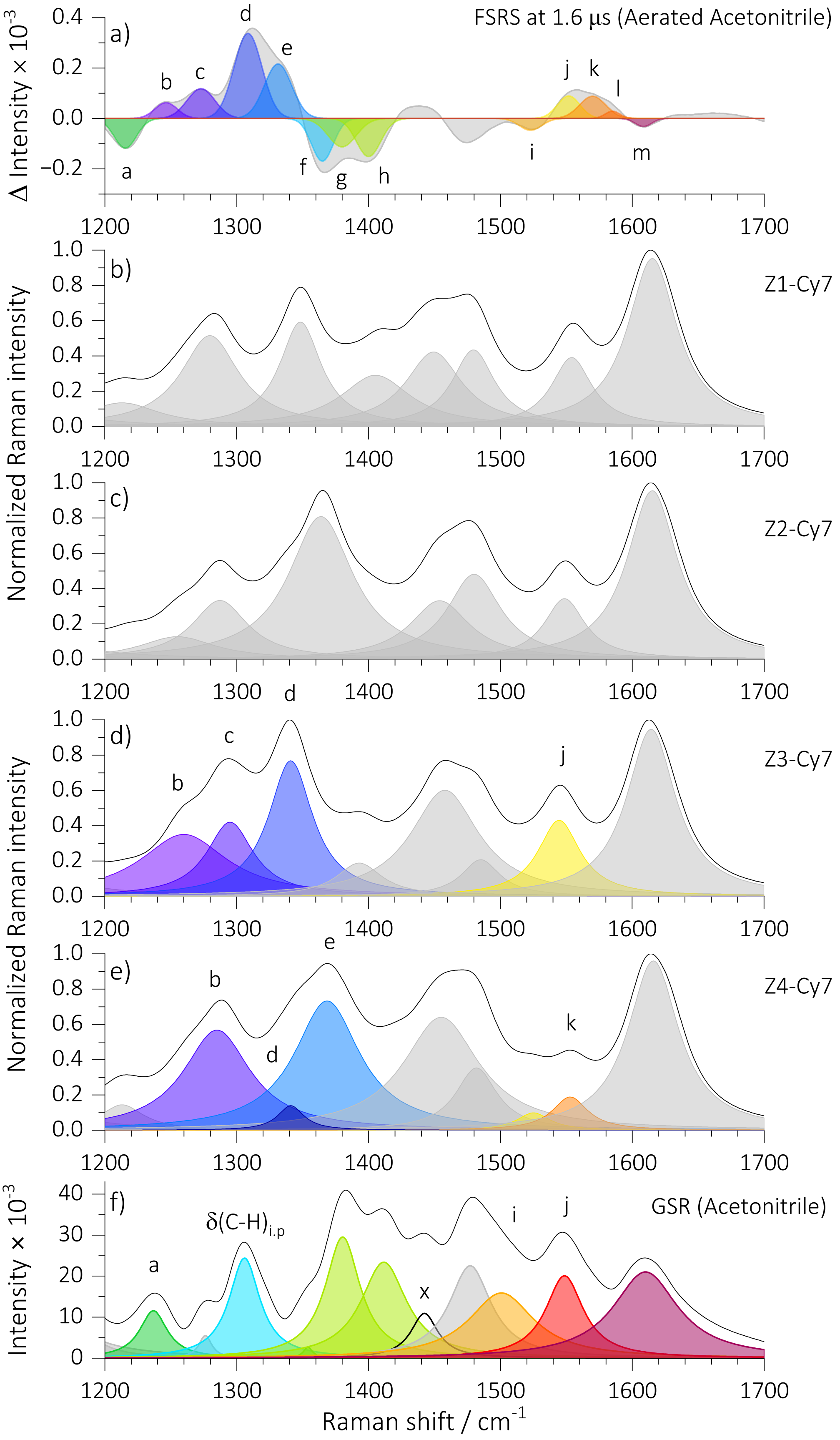

Figure S41. Comparison of a) experimental FSR spectrum at 1.6 us delay time, with calculated Raman spectra of b) ***Z1* Cy7**, c) ***Z2* Cy7**, d) ***Z3* Cy7**, e) ***Z4* Cy7**, and f) experimental GSR spectrum of **Cy7** in acetonitrile. The formation of two photoisomers, namely ***Z3*** and ***Z4* Cy7** was inferred from the spectra based on the band position and relative intensity comparison observed in experimental FSR, GSR and calculated spectra. One should bear in mind that the calculated spectra are normalized to the maximum intensity band, as a result, the band intensities are not directly comparable

between experimental and calculated spectra. Such comparison would not result in any meaningful conclusion. However, we compared the band position and the relative intensities, in other words, the ratios between bands within the calculated spectra were compared to the relative ratios observed in the FSR spectrum. The FSR spectrum (the relation between bands is connected through the color code): bands *a, f, g, h, l*, and *m* are the result of GSB in GSR. Bands *b, c* and *d* are the result of new vibration bands originating from ***Z*3 Cy7** (*b, c* and *d*) and ***Z*4 Cy7** (*b* and *d*) combined with GSB of $\delta_{i.p.}$ C-H in f). The band *e* is the result of new vibration bands originating from ***Z*4 Cy7** (*e*). Bands *j, k* and *l* are the result of new vibration bands originating from ***Z*3 Cy7** (*j*) and ***Z*4 Cy7** (*k*) combined with GSB of quadrant stretching vibration band of benzene ring (*j*) in f). The band *l* is a combination of GSB in GSR and a band *k* in ***Z*4 Cy7**, and lastly, the band labeled *x* in f) is a solvent signal (C–H bending) from CH_3CN.

6.2. TR-Raman spectra of species formed upon Cy7 excitation

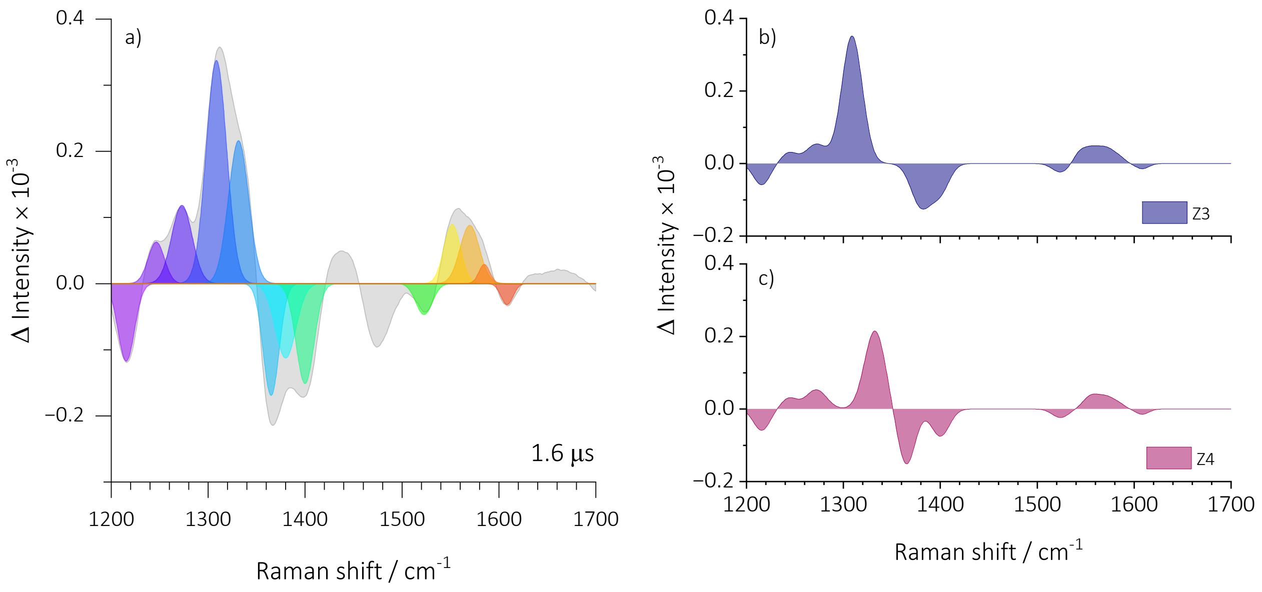

Figure S42. a) Experimental FSR spectrum of **Cy7** in aerated acetonitrile at the 1.6 µs. The obtained TR Raman spectra of b) ***Z*3 Cy7**, and c) ***Z*4 Cy7**.

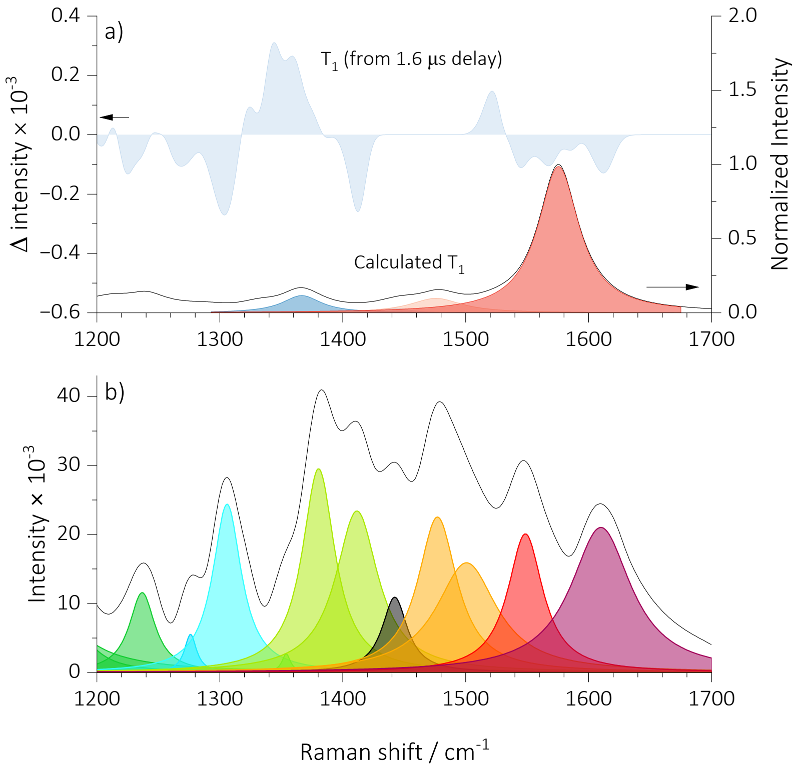

Figure S43. a) The TR-Raman spectrum of T_1 state obtained at 1.6 µs delay (top part of the figure) together with the calculated Raman spectrum of T_1 (bottom part of the figure), b) Experimental GSR of **Cy7** in aerated acetonitrile.

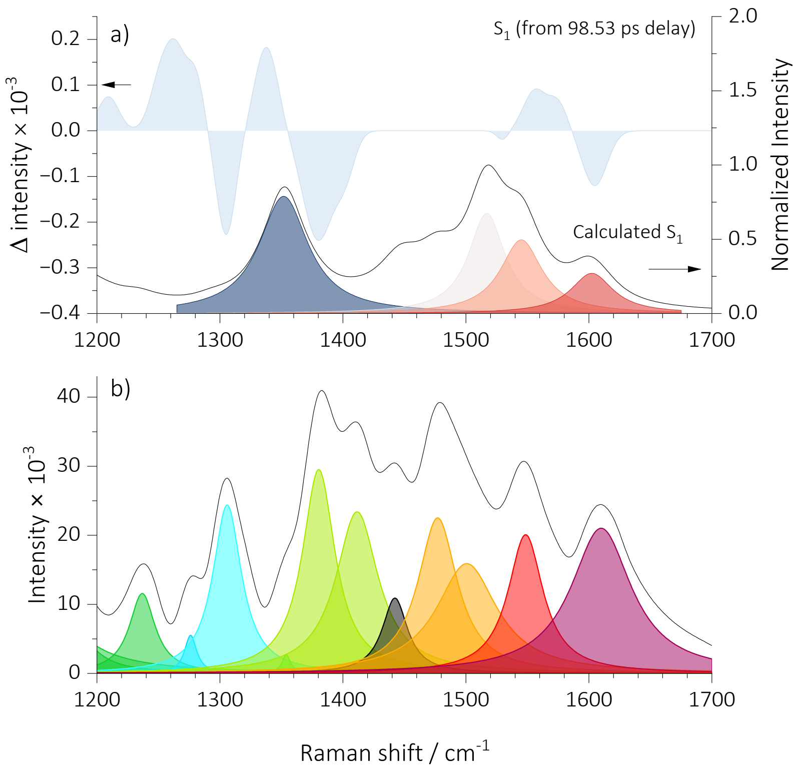

Figure S44. a) The TR-Raman spectrum of S_1 state obtained at 98.53 ps delay (top part of the figure) together with the calculated Raman spectrum of S_1 (bottom part of the figure); b) experimental GSR of **Cy7** in aerated acetonitrile.

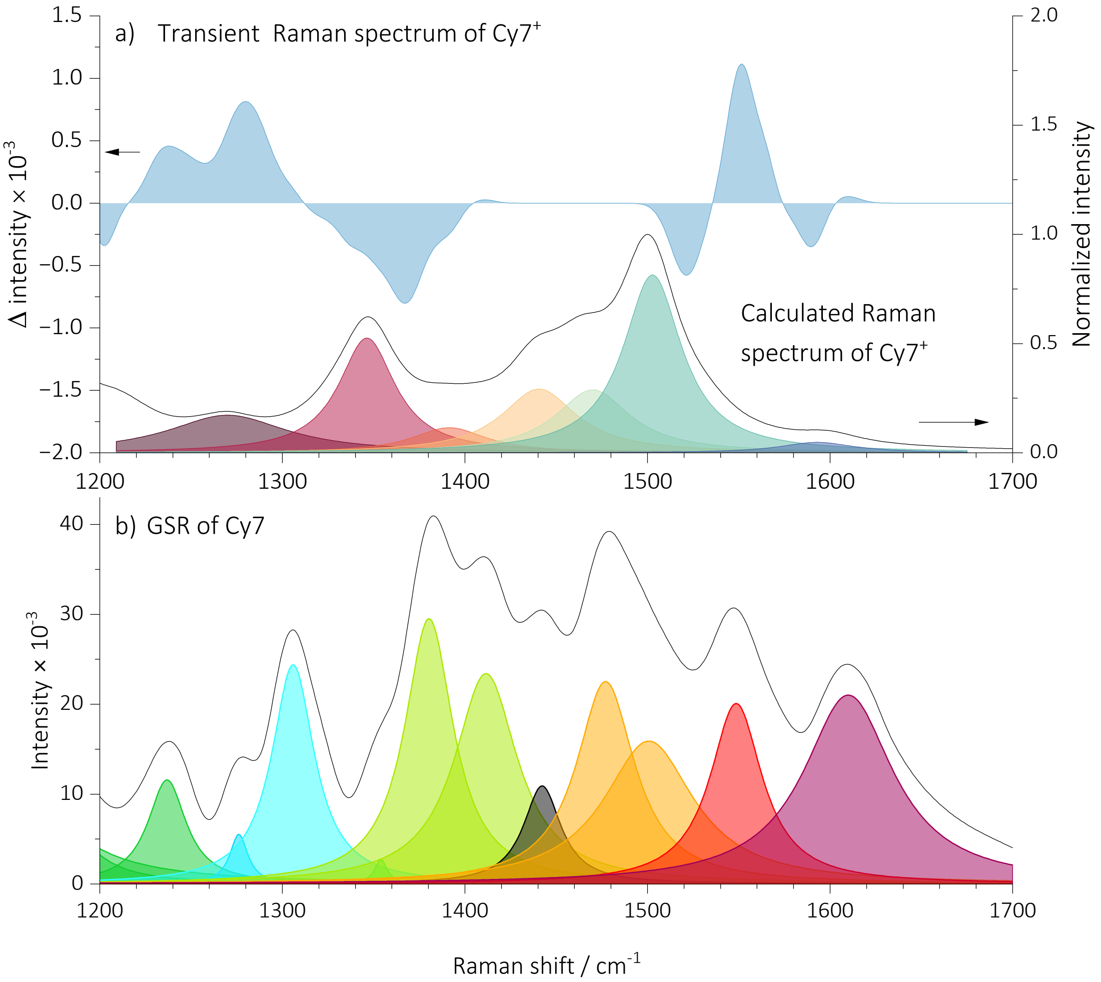

Figure S45. a) The TR-Raman spectrum of **Cy7•+** obtained at 2.56 ps delay (top part of the figure) together with calculated Raman spectrum of **Cy7•+** (bottom part of the figure), b) Experimental GSR of **Cy7** in aerated acetonitrile

6.3. Superoxide radical anion

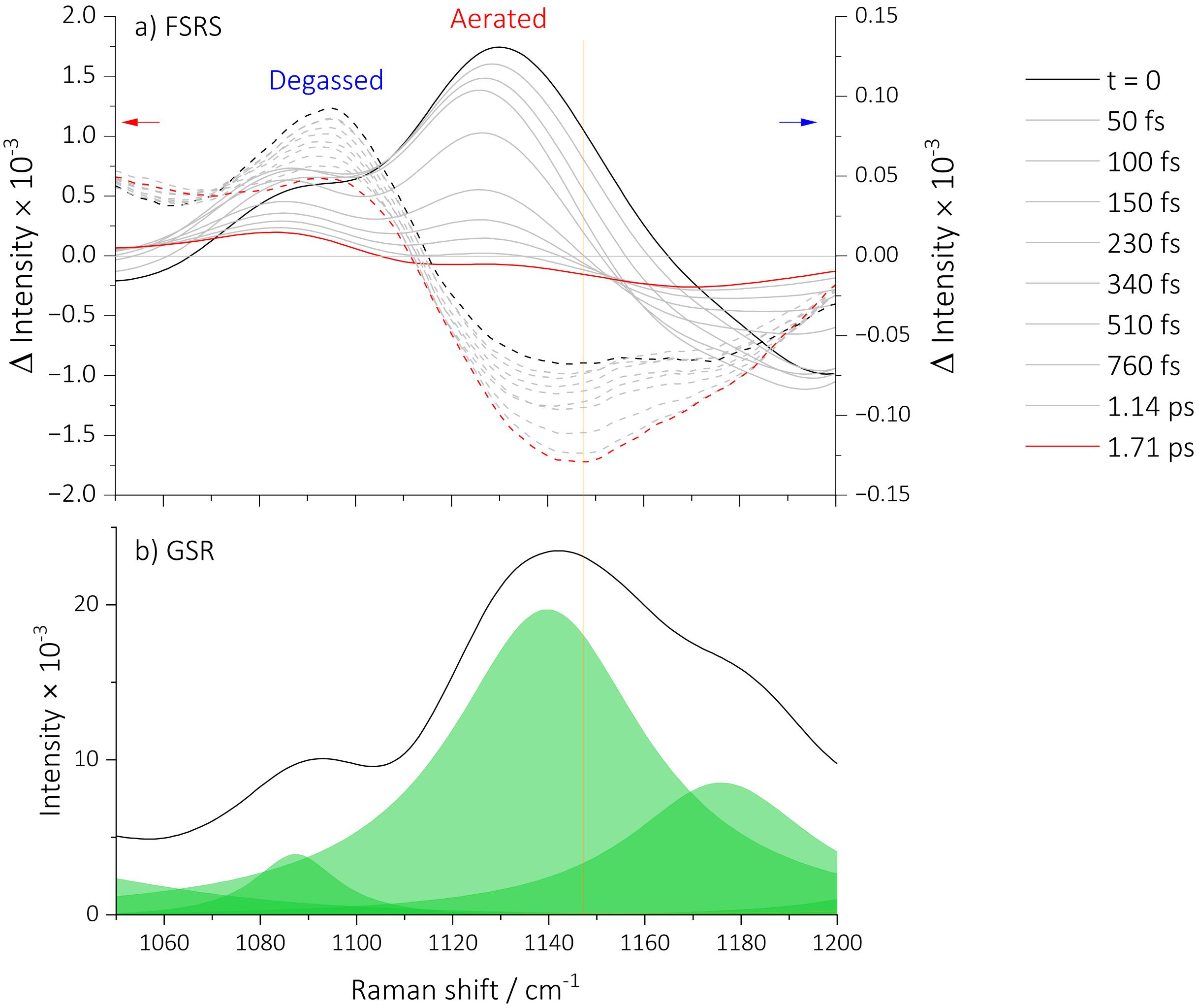

Figure S46. The a) experimental FSR spectra of **Cy7** in aerated (full line) and degassed (dotted line) acetonitrile, (0 to 1.71 ps time delays). b) The GSR of **Cy7** in acetonitrile. The expected ground-state bleach of the vibrational band at 1140 cm^{-1} is present under degassed conditions, on the other hand, under aerated conditions, there is a group of overlapping bands present (1060–1160 cm^{-1}), attributed to that of superoxide radical anion (1144 cm^{-1}) and **Cy7**$^{\bullet +}$ (1060–1140 cm^{-1}). If those bands would be the result of the solvent–excited **Cy7** interaction, they would be present under both (aerated and degassed) conditions. The superoxide radical anion band as a result of solvent-O_2 interaction is excluded because acetonitrile is one of the few solvents for which the formation of ground-state complex with O_2 was not observed.

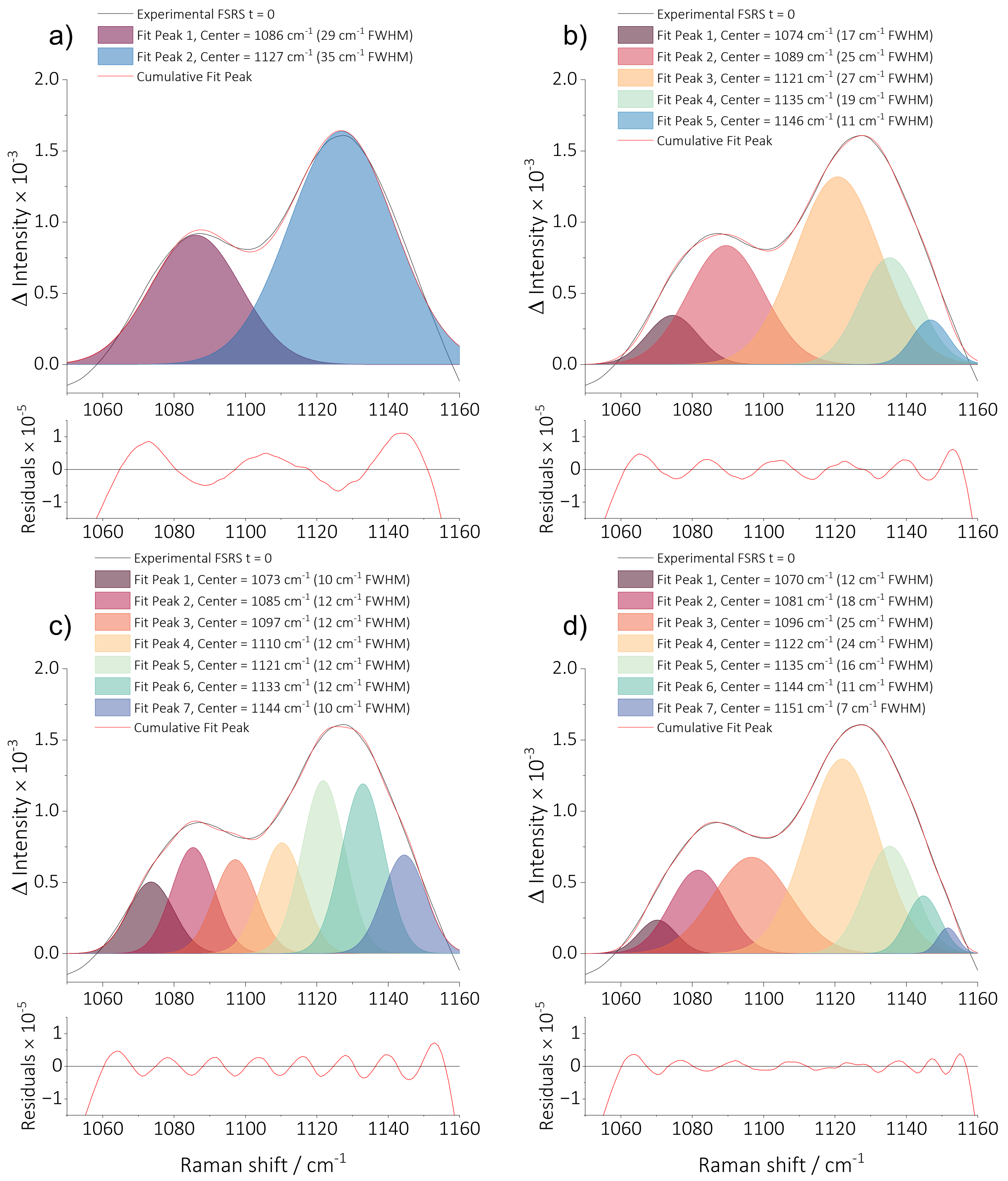

Figure S47. The experimental (black line) FSR spectrum at t = 0 recorded in aerated acetonitrile (Figure S46) in the 1050–1160 cm^{-1} range. The experimental bands were deconvoluted using a Gaussian function using the total of a) two, b) five, c) seven (FWHM limited to ≤ 12 cm^{-1}), or d) seven peaks. The red line is the cumulative peak fit, and the residuals are shown below each graph. The experimental FSR spectrum contains bands stemming from solvated **Cy7**$^{\bullet+}$, solvated hot S_1, and the superoxide radical anion band.

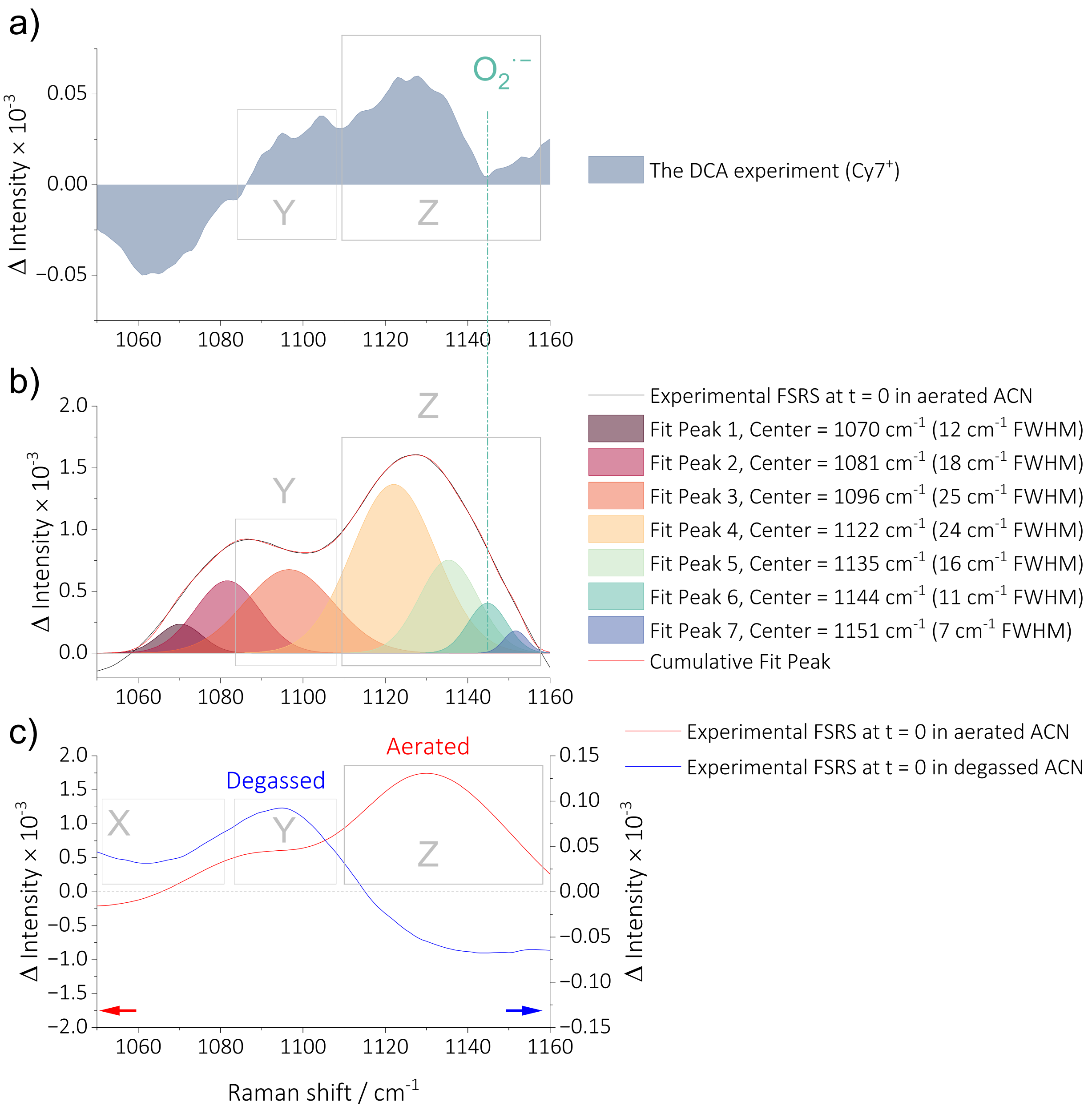

Figure S48. The superoxide radical anion band; a) The experimental FSR spectrum at 0 ps in the 1050–1160 cm^{-1} range from DCA experiment recorded in degassed acetonitrile. b) The experimental (black line) FSR spectrum at t = 0 in the 1050–1160 cm^{-1} range recorded in aerated acetonitrile. The experimental spectrum was deconvoluted using the total of seven gaussian peaks; the red line is the cumulative peak fit. c) The experimental FSR spectra of **Cy7** in aerated (red line) and degassed (blue line) acetonitrile at t = 0.

The comment on figure S48. Total of three different subregions are identified in the graphs on Figure S48, as indicated by rectangles **X**, **Y**, and **Z**. The rectangle **X** is assumed to contain bands from solvated hot S_1 state (FSR spectra recorded in degassed acetonitrile, *i.e.*, no **Cy7**$^{•+}$ formation is possible). This is further supported by the absence of the bands in this region in a DCA experiment (see Figure S48a)). The rectangle **Y** contains bands from both solvated hot S_1 state and solvated **Cy7**$^{•+}$ as indicated by the presence of the signals in FSR spectra under aerated and degassed conditions. Furthermore, the signals in this region are observed in DCA experiments as well where **Cy7**$^{•+}$ is present. Lastly, the rectangle **Z** contains exclusively the bands from solvated **Cy7**$^{•+}$ and superoxide radical anion bands, as the signals in this range are observed in acetonitrile under aerated conditions, in the DCA experiment, but are absent in acetonitrile under degassed conditions. The difference in a) and b) is that the DCA experiment is conducted under degassed conditions, where the band from $O_2^{•-}$ is not present. This clearly shows which is the $O_2^{•-}$ band.

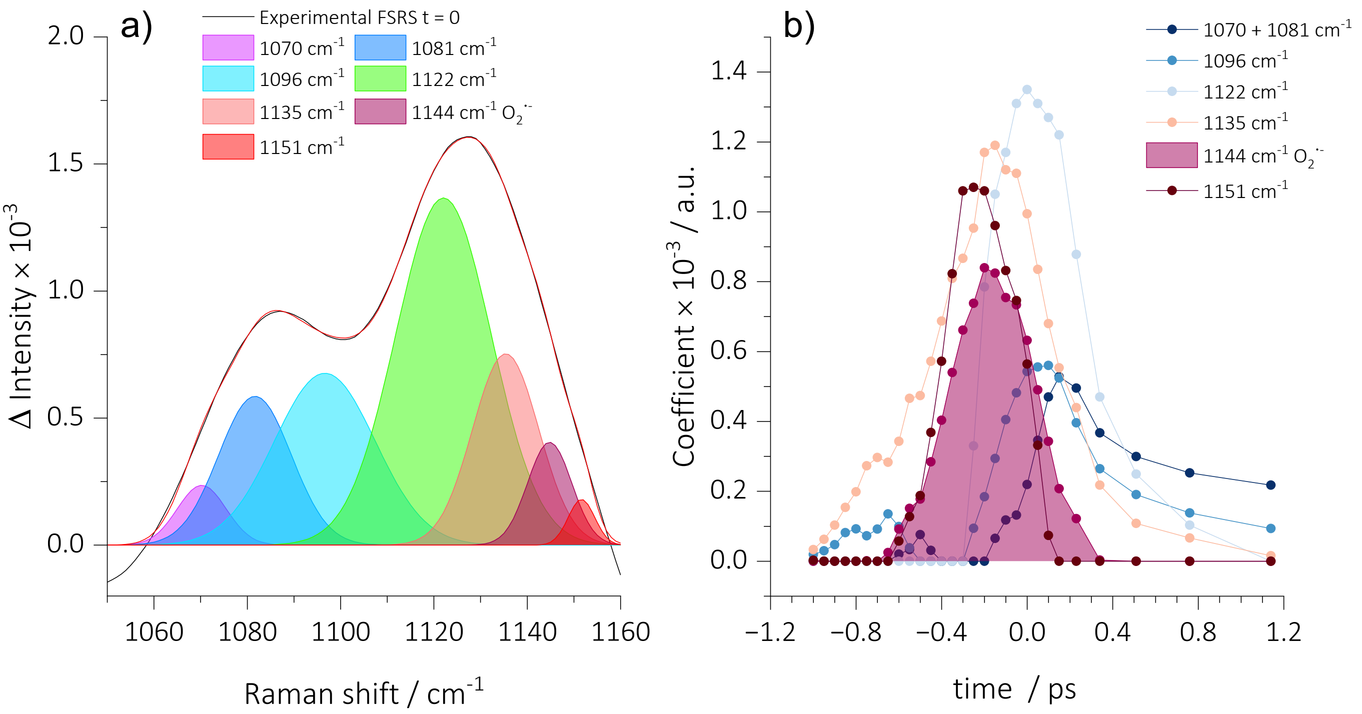

Figure S49. The superoxide radical anion band. a) Experimental (black line) FSR spectrum at the time delay t = 0 fitted with seven gaussian peaks (red line is the cumulative peak fit), some of which are assigned to **Cy7**$^{•+}$ and some to solvent (CH_3CN); the band at 1146 cm^{-1} is attributed to superoxide radical anion. b) Temporal evolution of the bands after fitting the experimental FSR spectra with six gaussian peaks from –1.0 to 1.14 ps

6.4. TR-Raman spectra of Cy7$^{•+}$ / the DCA experiment

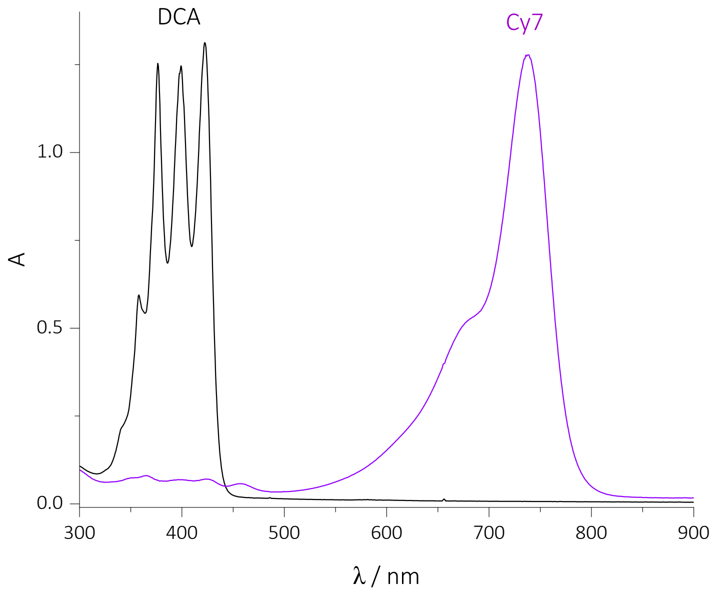

Figure S50. UVVis spectra of 9,10-Dicyanoanthracene (DCA) and **Cy7** in acetonitrile

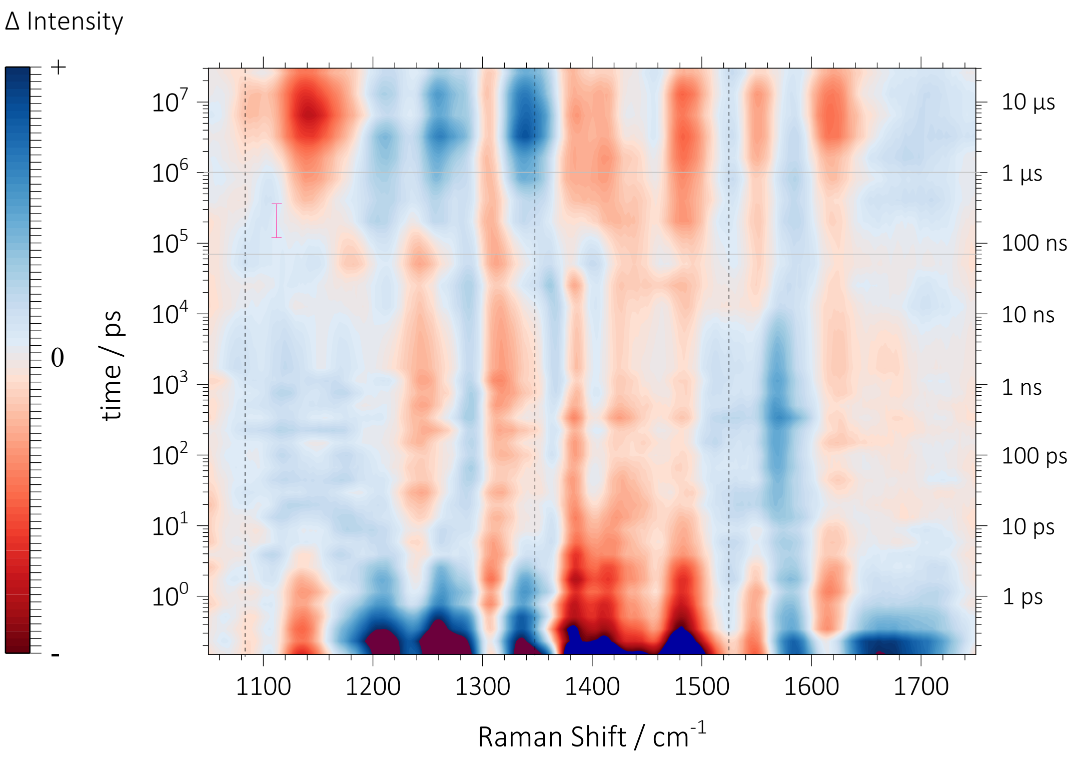

Figure S51. The DCA experiment. A contour plot of experimental FSR spectra obtained upon excitation of DCA (at 360 nm) in the presence of **Cy7** in degassed acetonitrile (freeze – pump – thaw).

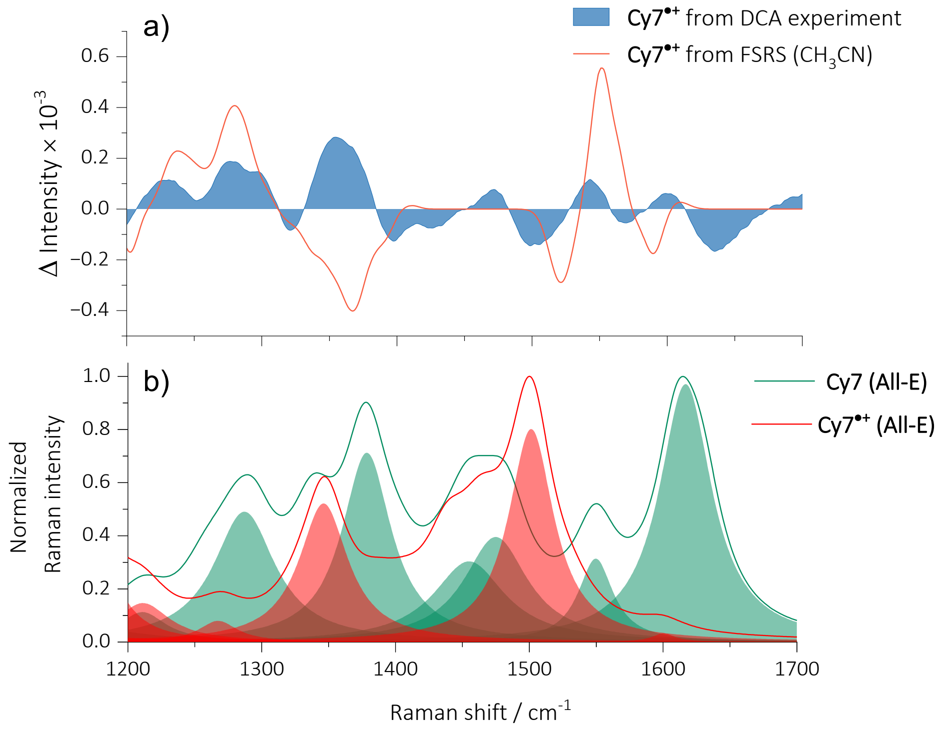

Figure S52. a) The TR-Raman spectrum of solvated **Cy7$^{•+}$** (orange solid line) obtained from experiment in aerated acetonitrile (Figure 7) at 2.56 ps delay time, together with the FSR Raman spectrum obtained from the DCA experiment (Figure S51) at 98 ps delay time. b) The calculated Raman spectrum of ***all-E* Cy7** (green line with) and ***all-E* Cy7$^{•+}$** (red line). The deconvoluted higher intensity bands of ***all-E* Cy7** (green) and ***all-E* Cy7$^{•+}$** (red) are those which are expected to contribute to the TR-Raman spectrum of **Cy7$^{•+}$** in terms of the new positive (***all-E* Cy7$^{•+}$** - red) and negative / GSB (***all-E* Cy7** -green) bands.

Table S3. Detailed assignment of transient Raman spectra of observed species, vibrational band shifts in cm^{-1}

Entry	Motion	Solvated Cy7$^{\bullet+}$	Solvated Hot S1	Cy7$^{\bullet+}$	Hot S_1	S_1	T_1	*Z*3	*Z*4
1	$\delta_{i.p.}$ (C–H), polyene chain			1300	1332	1337		1309	1332
	ν_s (C=C), polyene chain couple to ν_s C=C of aromatic rings							1572	1569
2	ν_s (C=C), polyene chain	1547		1549	1559	1558	1590<br>1519	1553	1554
3	$\delta_{i.p.}$ (C–H), aromatic protons	1301			1257	1257		1244	1244
4	Aromatic ring quadrant stretching	1603	1558		1577	1575			
5	CH_3 bending coupled with $\delta_{i.p.}$ (C–H, from polyene chain and acetonitrile)	1300	1325<br>And<br>1447						
6	Combination of C-H $\delta_{i.p.}$ from polyene chain and aromatic protons	1284<br>1254<br>1239	1281	1280<br>1251<br>1230	1282	1280	1323	1272	1271
7	(C–H) bending vibrations of CH_3	1473<br>1405<br>1383	1471		1351	1352	1360<br>1343		

7. Target analysis

time	FSR spectra in Aerated CH_3CN	FSR spectra in Degassed CH_3CN	Assumption	Extracting TR Raman spectrum
> 1 μs	Photoisomers	Photoisomers + T_1	T_1 quencehd by O_2 in aerated solvent	T_1 = Spectral difference between the FSR spectra recorded in aerated and degassed solvents, at 1.6 μs
≈ 100 ps	S_1 + T_1	S_1 + T_1		S_1 = Degassed FSR spectrum at 98.53 ps - T_1 Spectrum
≈ 3 ps	Hot S_1 + $Cy7^{•+}$	Hot S_1	Photoisomers not formed yet i.e., photoisomerisation proceeds from S_1 state[31]	Hot S_1 = Degassed FSR spectrum at 2.56 ps - S_1 Spectrum<br>$Cy7^{•+}$ = Spectral difference between the FSR spectra recorded in aerated and degassed solvents at 2.56 ps
500 fs	Solvated Hot S_1 + Solvated $Cy7^{•+}$	Solvated Hot S_1	Electron transfer to O_2	Solvated $Cy7^{•+}$ = Spectral difference between the FSR spectra recorded in aerated and degassed solvents at t = 0<br>Solvated Hot S_1 = Degassed FSR spectrum at t = 0

Figure S53. The schematic overview of the extraction procedure to obtain TR-Raman spectra of various species. Those TR-Raman spectra (blue color, right column) were used to construct target analysis models (see below).

Comparison of solvent-dependent FSR spectra

Due to the requirement of relatively high pulse intensities for FSR spectroscopy to obtain high-quality signals, photodegradation of the sample is a significant concern. It was determined that the use of acetonitrile at the requisite pulse intensities facilitates the acquisition of FSR spectra under both aerated (flow cell) and degassed (cuvette mounted on a movable platform for degassing) conditions. However, in other solvents, such as methanol and water, only aerated spectra could be obtained due to the combination of a large available sample volume and a continuously flowing fresh sample. The degassed spectra exhibited a predominant influence of sample degradation, attributable to the diminished photostability of **Cy7** in these solvents and the comparatively limited sample volume within the cuvette during the degassed measurements. Consequently, a comparison could only be made between the data obtained under aerated conditions for these solvents.

7.1. Target analysis models

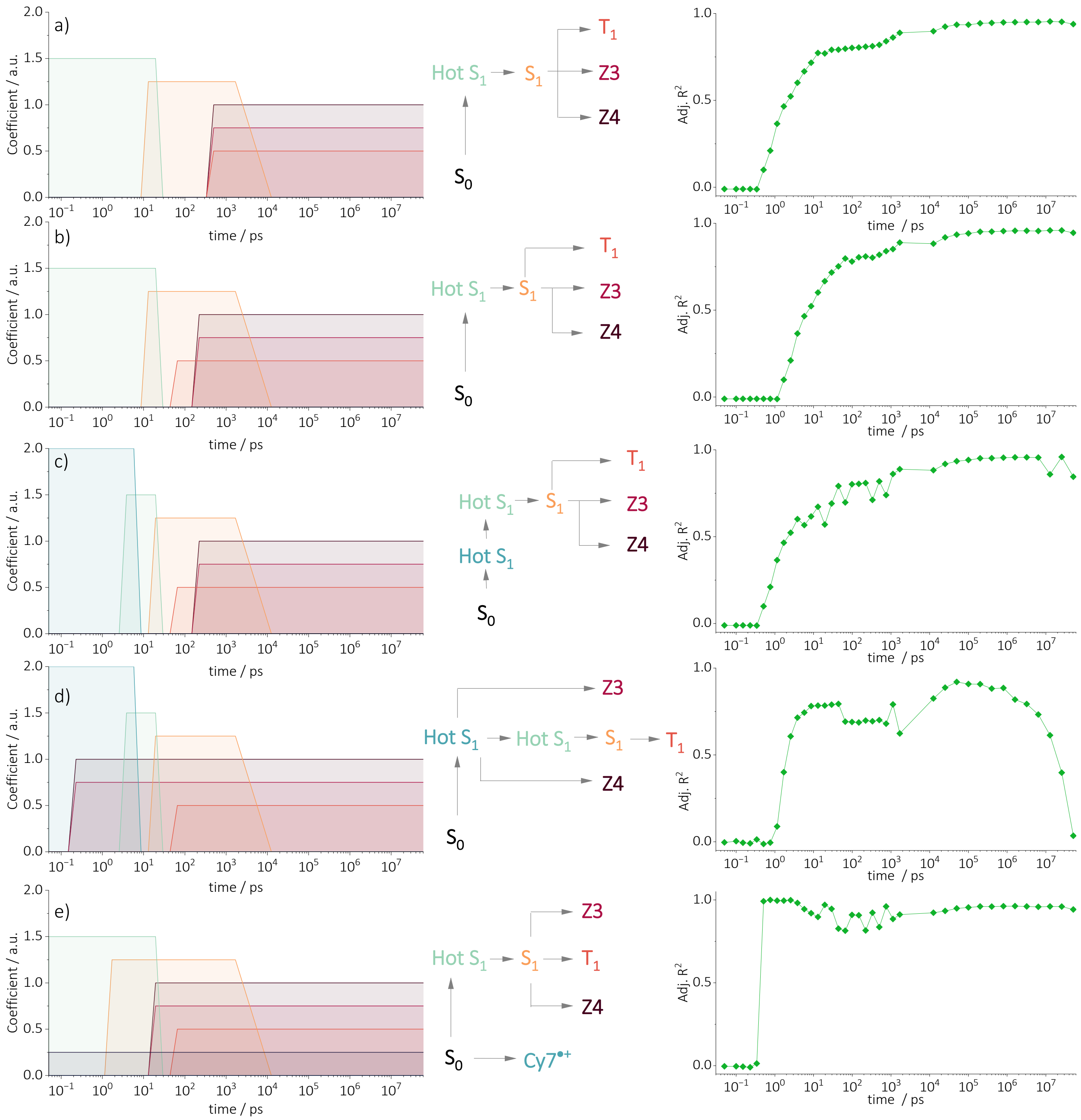

Figure S54. The first set of target analysis models used to fit the FSRS data, together with R^2 values obtained after fitting the FSRS data in aerated acetonitrile with respective models. The model e) produced a satisfactory fit and was used to fit the FSRS data in aerated acetonitrile (Figure S57), degassed acetonitrile (Figure S58), aerated methanol (Figure S59) and water (Figure S60).

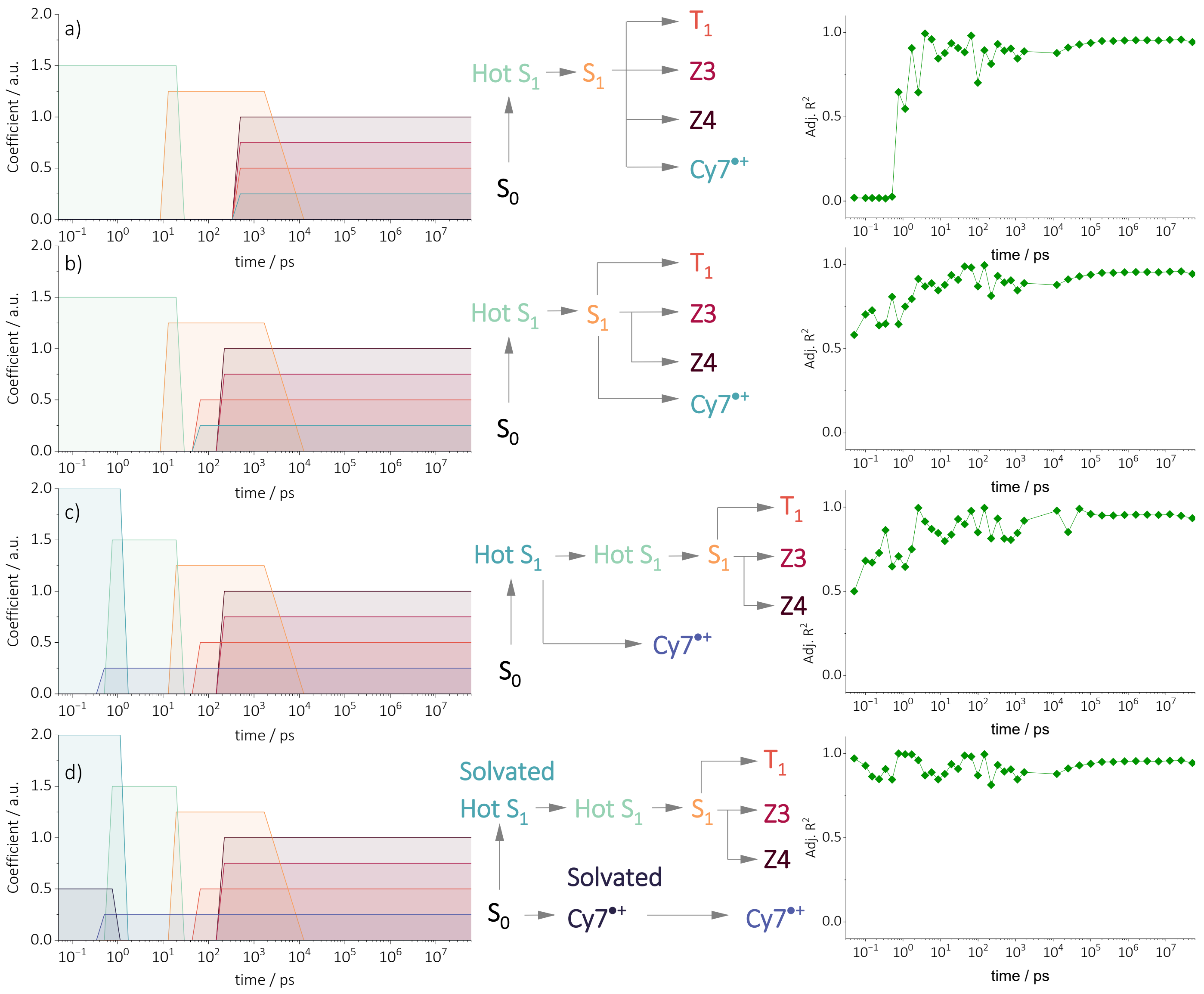

Figure S55. The set of second target analysis models used to fit the FSRS data, together with R^2 values obtained after fitting the FSRS data in aerated acetonitrile with respective models. The model d) produced satisfactory fit and was used to fit the FSRS data in aerated acetonitrile (Figure 9).

Best fitting kinetic model for FSR data

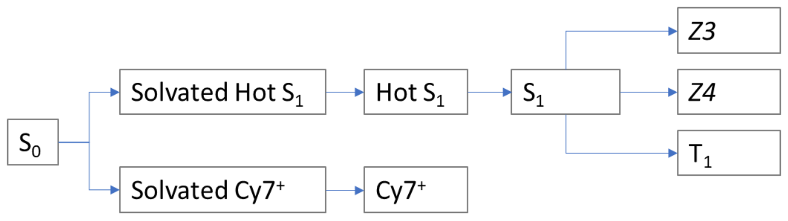

Best fitting kinetic model for fs-TA data

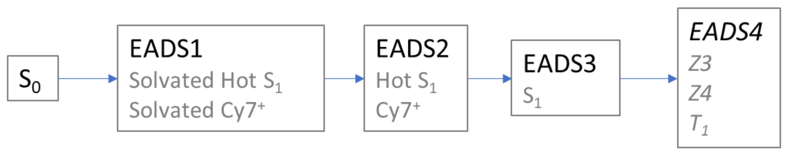

Figure S56. The comparison of the kinetic models used for fitting the FSR data (Figure S54d) and sequential 4 components model used for fs-TA data (Figure S9 and S10).

7.2. Fitted FSR data

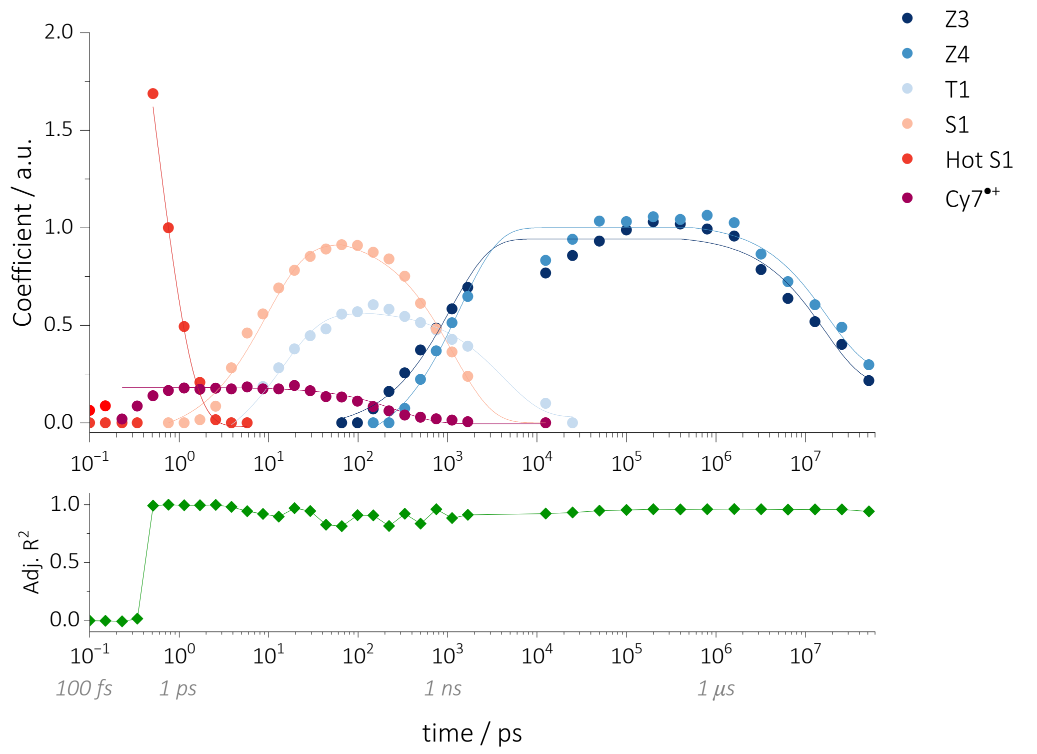

Figure S57. The output of target analysis model (Figure S54e) upon fitting the FSR spectra of **Cy7** in aerated acetonitrile with R^2 shown in the bottom of the figure. The solid lines show fit of the model output coefficients with *ExpGrowDec* function to extract lifetimes of various **Cy7** species.

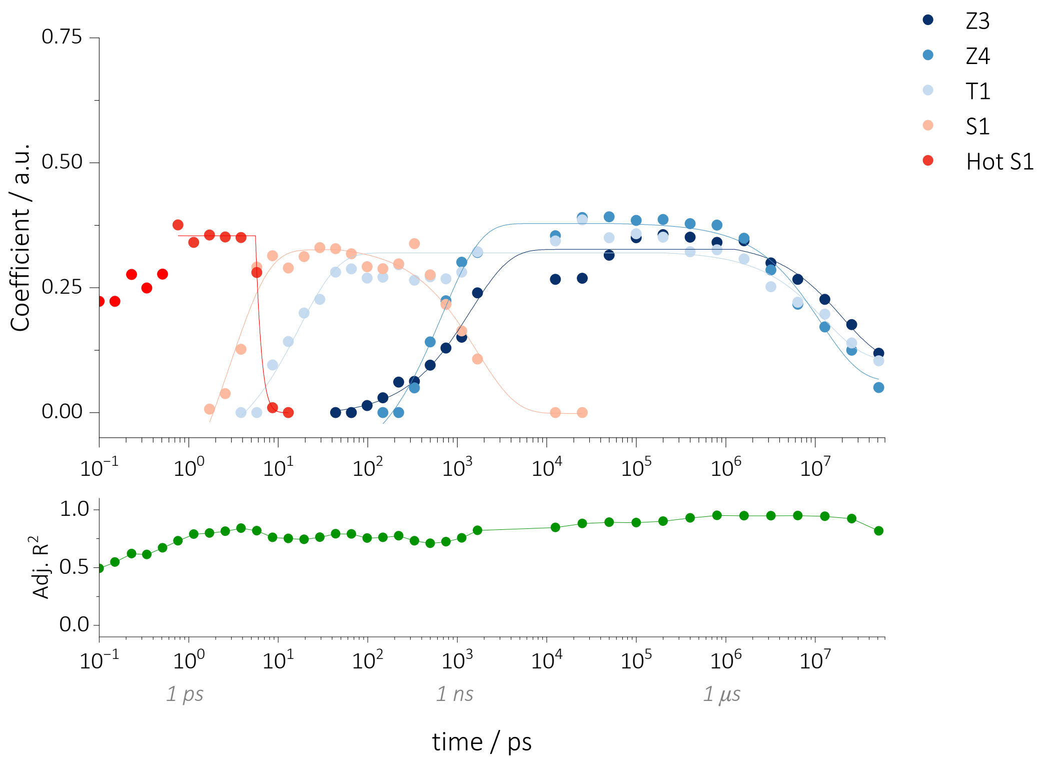

Figure S58. The output of the target analysis model (Figure S54e) upon fitting the FSR spectra of **Cy7** in degassed acetonitrile with R^2 shown in the bottom of the figure. The solid lines show fit of the model output coefficients with *ExpGrowDec* function to extract lifetimes of various **Cy7** species.

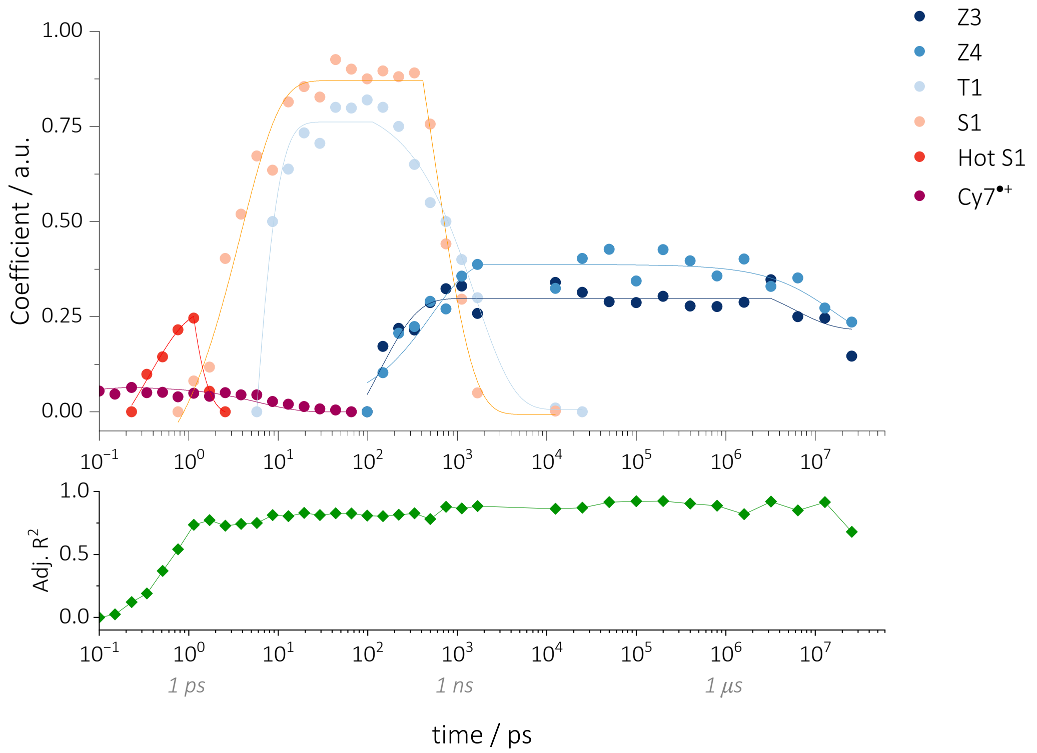

Figure S59. The output of target analysis model (Figure S54e) upon fitting the FSR spectra of **Cy7** in aerated methanol with R^2 shown in the bottom of the figure. The solid lines show fit of the model output coefficients with *ExpGrowDec* function to extract lifetimes of various **Cy7** species.

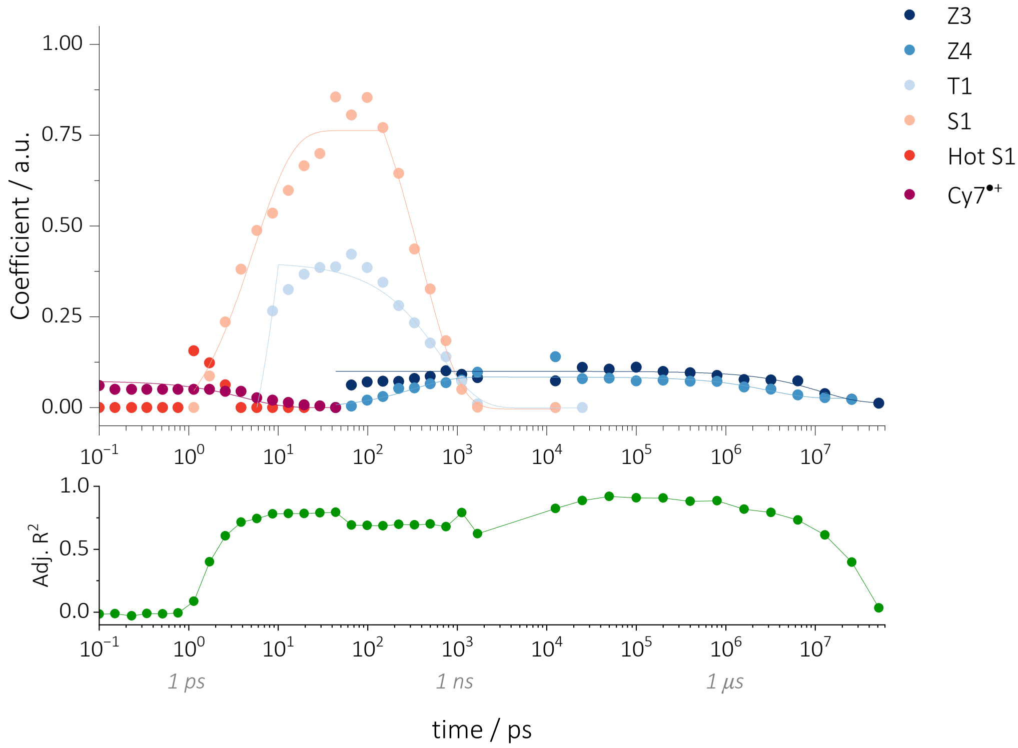

Figure S60. The output of target analysis model (Figure S54e) upon fitting the FSR spectra of **Cy7** in aerated water (+ 5% methanol) with R^2 shown in the bottom of the figure. The solid lines show fit of the model output coefficients with *ExpGrowDec* function to extract lifetimes of various **Cy7** species. Overall poor R^2 is observed due to the small signal intensity (lower S/N ratio).

8. Synthesis of Cy7-2’,6’-d_2

The synthesis of **Cy7**-2’,6’-*d*$_2$ is shown in Scheme 1. In brief, commercially available 2,6-dibromopyridine (**1**) was converted to pyridine-2,6-*d*$_2$ *via* lithium–halogen exchange followed by the reaction with D_2O, and the resulting crude product was converted to the corresponding Zincke salt (**3**, Figure S61 and S62). The final **Cy7**-2’,6’-*d*$_2$ (**5**) was obtained by the reaction of **5** with 1,2,3,3-tetramethyl-3*H*-indolium iodide (**4**) according to a previously published procedure.[1] The crude cyanine derivative was then purified by flash column chromatography (Figures S63 and S64).

Scheme 1. The synthesis of **Cy7**-2’,6’-d_2 (**5**).

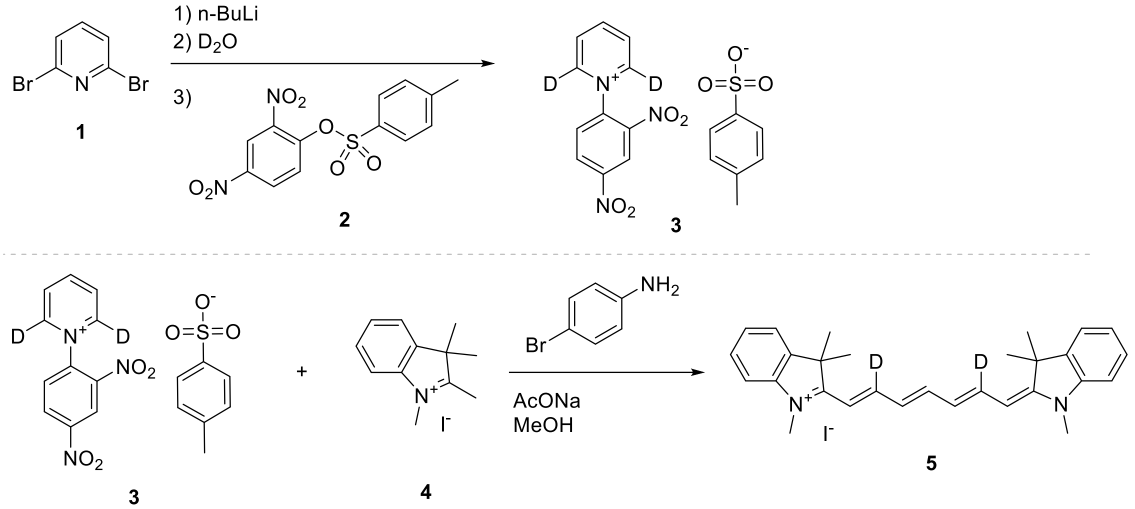

Preparation of 1-(2,4-Dinitrophenyl)pyridin-1-ium-2,6-d$_2$ (3).

Pyridine-2,6-d$_2$. 2,6-Dibromopyridine (**1**, 3.00 g, 12.6 mmol) in anhydrous Et_2O (40 mL) was added dropwise to a solution of *n*-BuLi (2.5 M in hexane, 13.5 mL, 1.2 mmol) in dry Et_2O (15 mL) under inert atmosphere at –78°C during 20 min. The resulting solution was stirred at –78°C for 1 h, after which D_2O (1.5 mL, 75.98 mmol) was added to the solution, and the reaction mixture was allowed to warm up to room temperature. The product (pyridine-2,6-*d*$_2$) was extracted with Et_2O (3 × 40 mL), the organic layers were combined and dried over Na_2SO_4, and the solvent was evaporated under reduced pressure. The crude product was used without purification in the next step.

*Zincke salt (**3**);* A mixture of 2,4-dinitrophenyl-*p*-toluenesulfonate (**2**, 450 mg, 1.36 mM) and pyridine-2,6-*d*$_2$ from the previous step (100 mg, 1.23 mM) were dissolved in toluene (8 mL). The reaction mixture was refluxed for 16 h, after which the precipitate was formed upon cooling to room temperature. The resulting precipitate was filtered, washed with toluene (2 × 5 mL), Et_2O (2 × 5 mL), and dried to give a white solid (**3**). Yield: 230 mg (45%). ^{1}H NMR (500 MHz, DMSO-d_6) δ 9.12 (d, *J* = 2.5 Hz, 1H), 9.01–8.92 (m, 2H), 8.42 (t, *J* = 8.4 Hz, 3H), 7.46 (d, *J* = 8.1 Hz, 2H), 7.10 (d, *J* = 7.8 Hz, 2H) ppm. ^{13}C NMR (126 MHz, DMSO-d_6) δ 149.1, 148.9, 145.9, 143.0, 138.6, 137.4, 131.8, 130.1, 128.0, 127.9, 125.4, 121.4, 40.1, 20.7. HRMS (ESI$^+$): calcd. for $C_{11}H_6D_2N_3O_4^+$ [M–pTsOH$^-$] 248.0635, found 248.0648.

Preparation of 1,3,3-trimethyl-2-((1E,3E,5E)-7-((E)-1,3,3-trimethylindolin-2-ylidene)hepta-1,3,5-trien-1-yl-2,6-d$_2$)-3H-indol-1-ium iodide (5).

The 4-bromoaniline (86 mg 0.50 mM) and **3** (100 mg, 0.24 mM) were dissolved in methanol (7 mL), and the reaction mixture was stirred at room temperature for 30 min. Next, 1,2,3,3-tetramethyl-3*H*-indolium iodide (**4**, 220 mg , 0.74 mM) and sodium acetate (117 mg, 1.43 mM) were added, and the reaction mixture was stirred for another 16 h at room temperature. Afterward, Et_2O (20 mL) was added, and the mixture was placed in a freezer (−16 °C). The resulting precipitate was filtered, washed with water (2 × 5 mL) and Et_2O (2 × 5 mL) and dried on air. The crude product was purified by flash column chromatography (silica gel, 5% methanol/dichloromethane); the solvents were removed under reduced pressure to give **5** as a green solid. Yield: 85 mg (66%). ^{1}H NMR (500 MHz, MeOD-d_4) δ 7.63 (t, *J* = 12.7 Hz, 1H), 7.47 (d, *J* = 7.4 Hz, 2H), 7.43–7.35 (m, 2H), 7.24 (dd, *J* = 14.3, 7.5 Hz, 4H), 6.55 (d, *J* = 12.7 Hz, 2H), 6.25 (s, 2H), 3.60 (s,

6H), 1.69 (s, 12H) ppm. ^{13}C NMR (126 MHz, MeOD-d_4) δ 174.0, 144.4, 142.3, 129.7, 126.6, 123.2, 111.6, 104.6, 50.2, 31.5, 27.9. HRMS (ESI$^+$): calcd. for $C_{29}H_{31}N_2^+$ [M–I$^-$] 411.2764, found 411.2768.

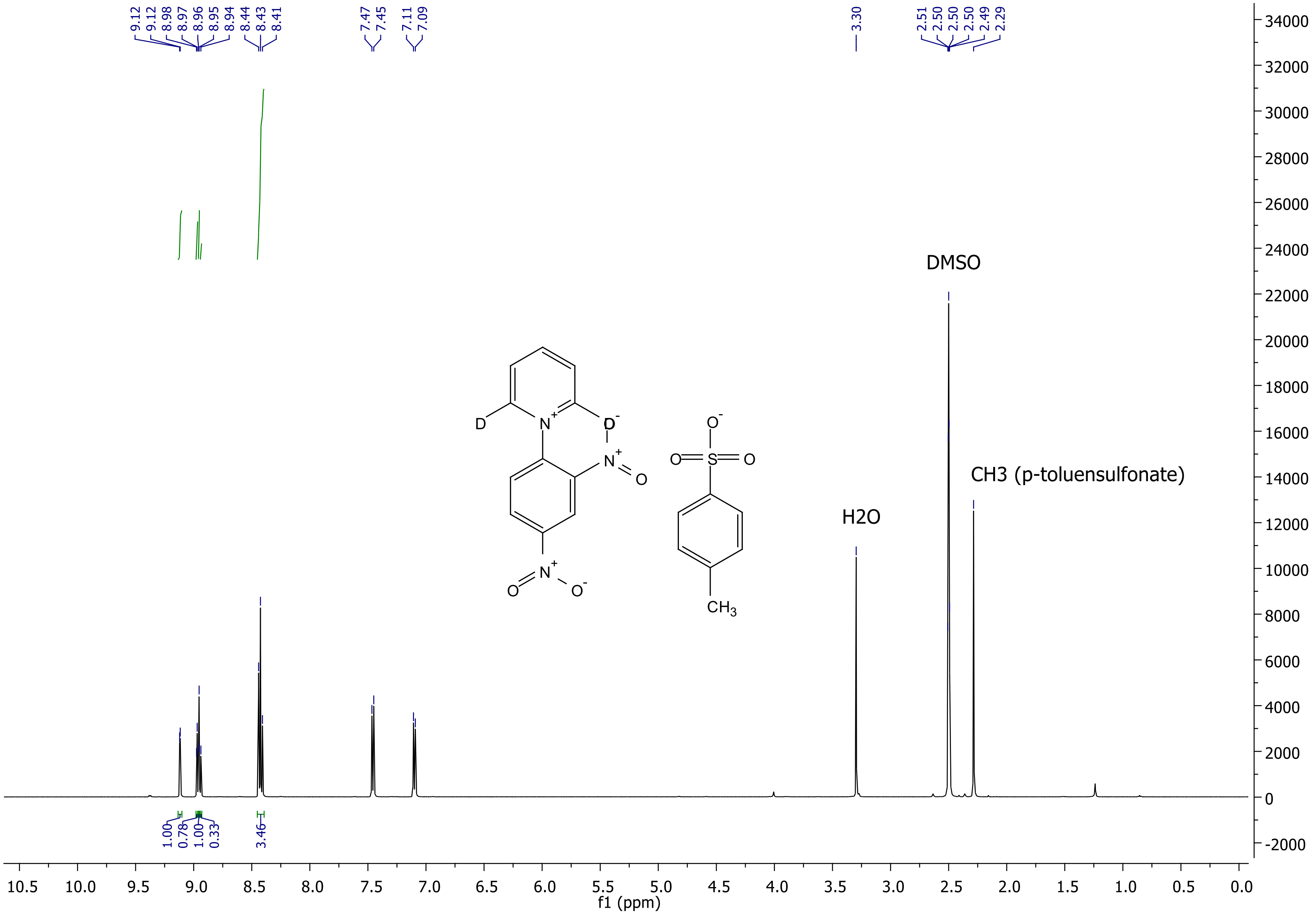

Figure S61. ^{1}H NMR (500 MHz, DMSO-d_6) of **3** .

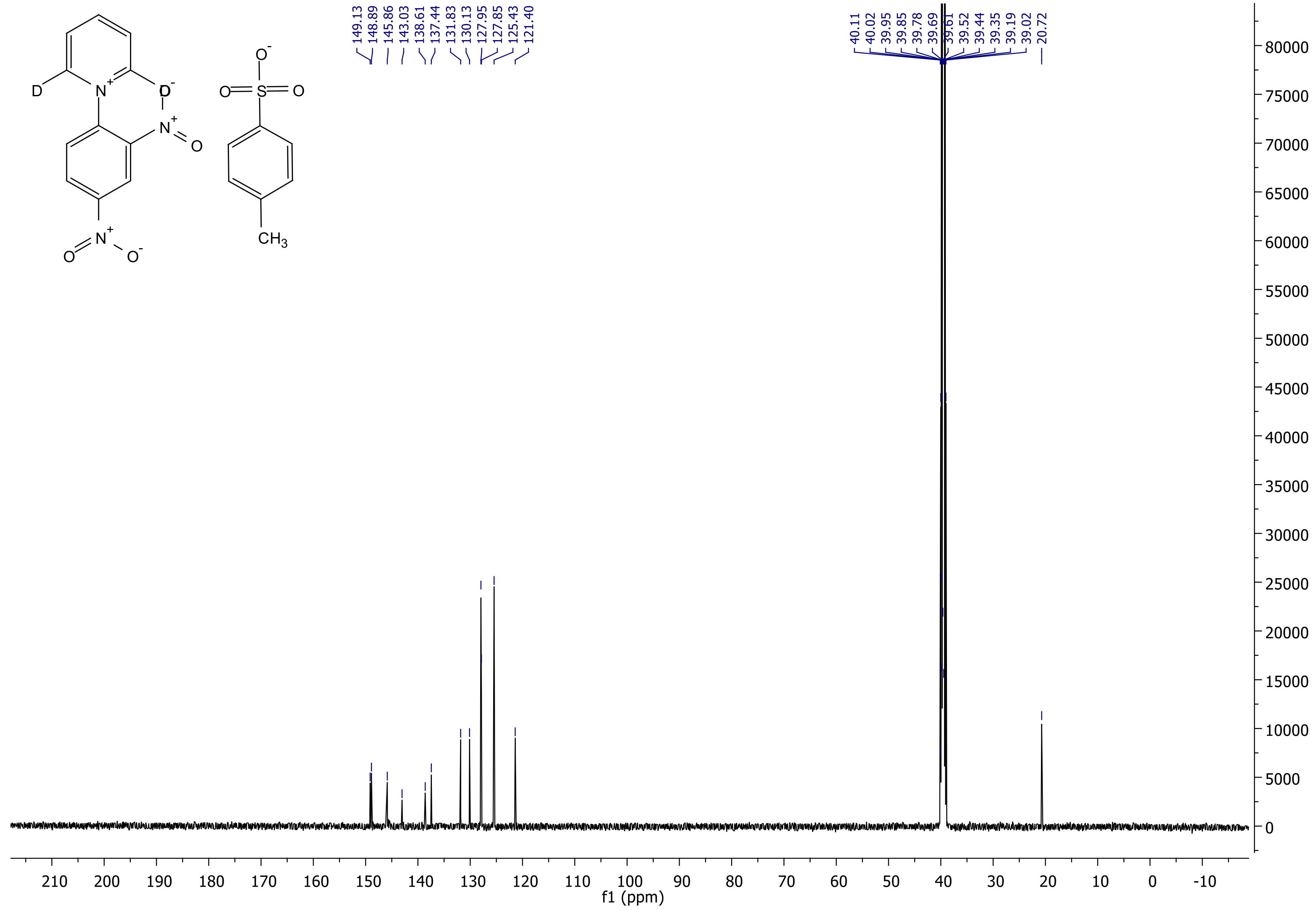

Figure S62. ^{13}C NMR (126 MHz, DMSO-d_6) of **3**.

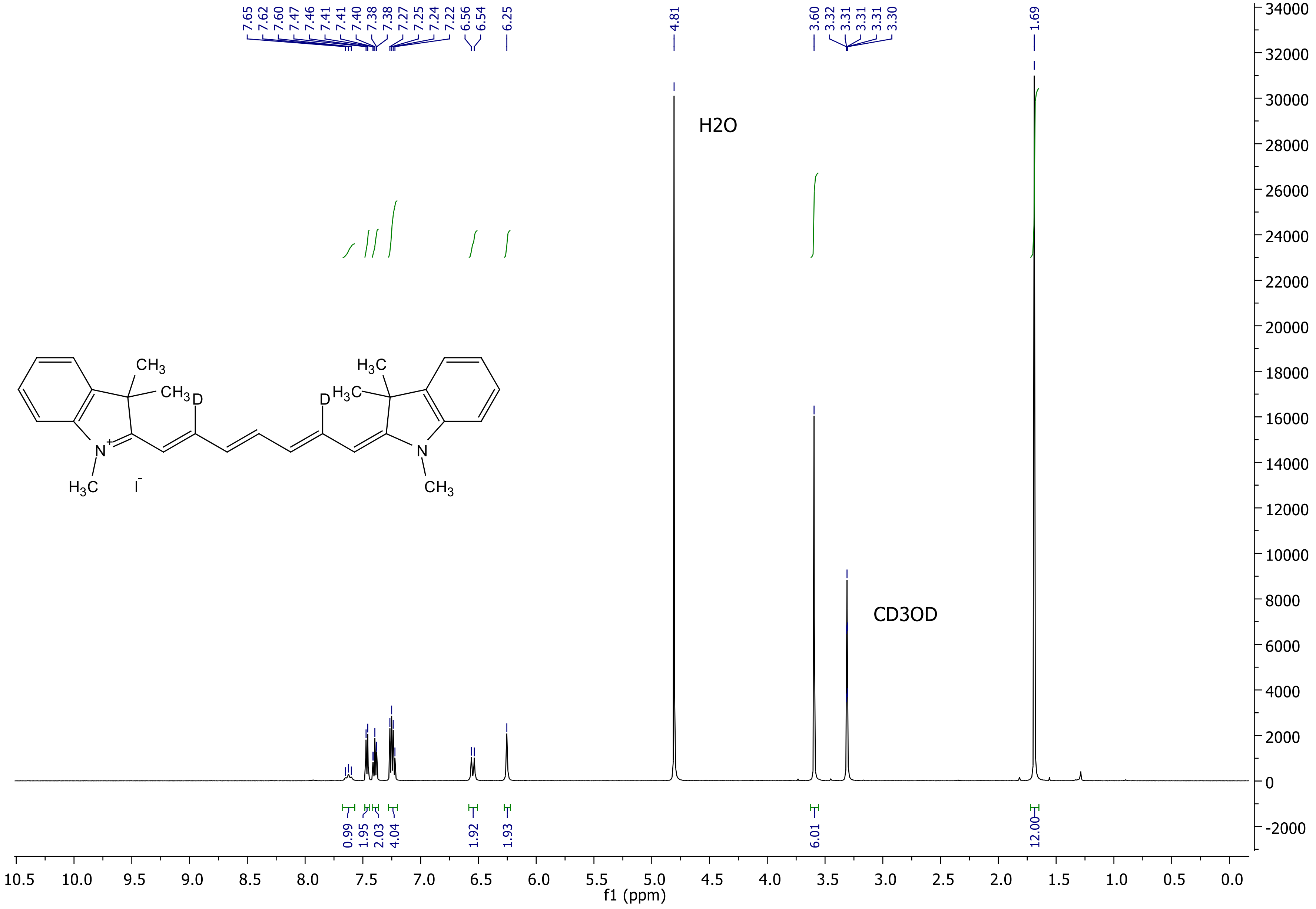

Figure S63. ^{1}H NMR (500 MHz, CD_3OD-d_4) of **5**.

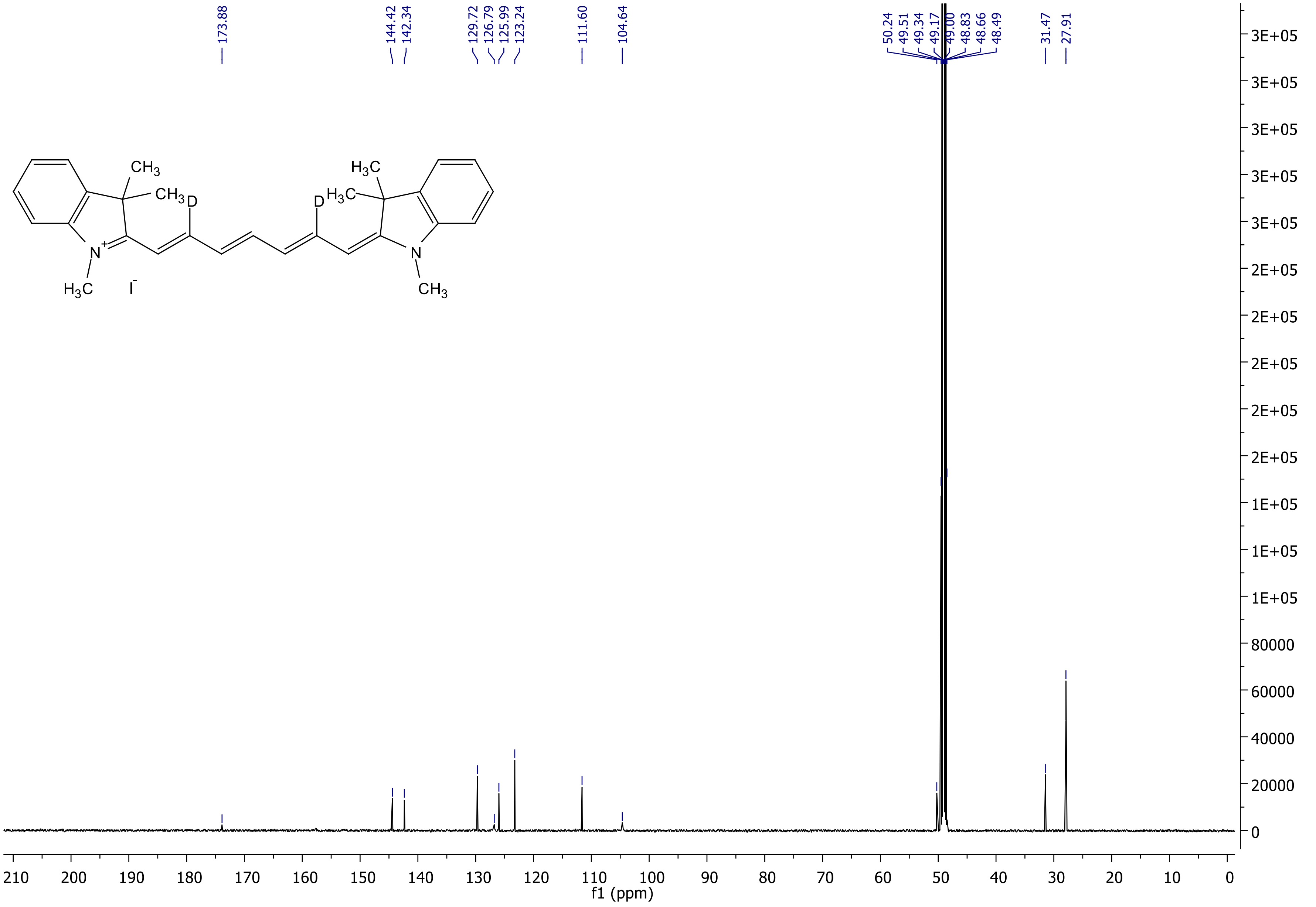

Figure S64. ^{13}C NMR (126 MHz, CD_3OD-d_4) of **5**.

9. **Cartesian Coordinates for structures optimized at the ωB97X-D/6-31G* level of theory**

All-*E* (S_0)

C	8.884056	-1.823477	0.000390
C	9.386842	-0.524148	0.000359
C	8.535696	0.581534	0.000287
C	7.172342	0.330761	0.000248
C	6.654346	-0.958768	0.000277
C	7.506345	-2.048862	0.000349
C	5.141462	-0.897274	0.000220
C	4.586069	-1.559722	1.275982
C	4.586158	-1.559825	-1.275527
C	4.903705	0.616091	0.000151
N	6.095475	1.247762	0.000175
C	6.249989	2.689880	0.000128
C	3.701082	1.312567	0.000074
C	2.428644	0.736787	0.000051
C	1.239200	1.453319	-0.000027
C	0.000000	0.817298	-0.000043
C	-1.239200	1.453319	-0.000118
C	-2.428644	0.736787	-0.000125
C	-3.701082	1.312567	-0.000199
C	-4.903705	0.616091	-0.000206
N	-6.095475	1.247762	-0.000280
C	-5.141462	-0.897274	-0.000138
C	-4.586068	-1.559838	-1.275839
C	-6.654346	-0.958768	-0.000190
C	-4.586159	-1.559709	1.275670
C	-6.249989	2.689880	-0.000356
C	-7.172342	0.330761	-0.000273
C	-8.535696	0.581534	-0.000336
C	-9.386842	-0.524148	-0.000312
C	-8.884056	-1.823477	-0.000228
C	-7.506345	-2.048862	-0.000166
H	9.568080	-2.665332	0.000447
H	10.459795	-0.363078	0.000392
H	8.946277	1.585206	0.000262
H	7.115251	-3.062421	0.000373
H	3.494628	-1.520742	1.317409
H	4.887681	-2.611273	1.297483
H	4.982077	-1.074421	2.172387
H	4.887792	-2.611372	-1.296935
H	3.494720	-1.520869	-1.317022
H	4.982211	-1.074581	-2.171943
H	7.308859	2.940546	0.000182
H	5.788804	3.122147	-0.892910
H	5.788698	3.122215	0.893078
H	3.743615	2.397790	0.000028
H	2.341374	-0.346829	0.000097
H	1.274126	2.540907	-0.000077
H	0.000000	-0.274603	0.000010
H	-1.274126	2.540907	-0.000173
H	-2.341374	-0.346829	-0.000068
H	-3.743615	2.397790	-0.000257
H	-3.494627	-1.520864	-1.317267
H	-4.887683	-2.611390	-1.297248
H	-4.982072	-1.074615	-2.172289
H	-3.494721	-1.520746	1.317164
H	-4.982216	-1.074387	2.172042
H	-4.887790	-2.611255	1.297170
H	-5.788728	3.122136	-0.893360
H	-7.308859	2.940546	-0.000395
H	-5.788773	3.122225	0.892628
H	-8.946277	1.585206	-0.000403
H	-10.459795	-0.363078	-0.000360
H	-9.568080	-2.665332	-0.000210
H	-7.115251	-3.062421	-0.000101

*Z*1 (S_0)

C	-9.392150	0.898934	0.001840
C	-8.775765	2.147973	0.001207
C	-7.385577	2.269405	0.000253
C	-6.647096	1.096348	-0.000102
C	-7.246452	-0.157370	0.000604
C	-8.625643	-0.267779	0.001568
C	-6.175795	-1.220479	0.000100
C	-6.251970	-2.092256	-1.266919
C	-6.250559	-2.092122	1.267271
C	-4.900299	-0.374958	-0.000750
N	-5.235828	0.930020	-0.001133
C	-4.308772	2.041680	-0.002961
C	-3.643948	-0.970072	-0.001118
C	-2.363440	-0.408085	-0.000584
C	-1.195436	-1.161287	-0.000779
C	0.065061	-0.569804	-0.000207
C	1.278805	-1.253726	-0.000298
C	2.496504	-0.586978	0.000055
C	3.743405	-1.216284	0.000064
C	4.974808	-0.572614	0.000195
N	6.137651	-1.256209	0.000123
C	7.253692	-0.387247	0.000148
C	6.228232	-2.703502	0.000027
C	5.278662	0.928579	0.000308
C	4.752680	1.614364	-1.275446
C	6.792574	0.923830	0.000231
C	4.752858	1.614271	1.276181
C	7.691515	1.975495	0.000269
C	9.058132	1.690063	0.000228
C	9.503399	0.370011	0.000158
C	8.604753	-0.697398	0.000118
H	-10.474946	0.832816	0.002595
H	-9.381868	3.047853	0.001472
H	-6.927341	3.252103	-0.000078
H	-9.106005	-1.241916	0.002098
H	-5.440010	-2.825210	-1.292293
H	-7.200891	-2.636910	-1.277048

H	-6.199619	-1.480966	-2.172225
H	-6.197375	-1.480726	2.172455
H	-7.199390	-2.636914	1.278417
H	-5.438464	-2.824949	1.291874
H	-4.864000	2.977393	-0.005273
H	-3.681750	2.015958	0.893564
H	-3.680953	2.012082	-0.898794
H	-3.675318	-2.056425	-0.001503
H	-2.237455	0.670000	0.000302
H	-1.266569	-2.247205	-0.001446
H	0.106904	0.521393	0.000332
H	1.269700	-2.341906	-0.000700
H	2.455132	0.499432	0.000310
H	3.740148	-2.302432	-0.000106
H	5.748984	-3.115157	-0.893183
H	7.275121	-3.000625	-0.000144
H	5.749230	-3.115257	0.893324
H	3.660639	1.617743	-1.320638
H	5.094814	2.653594	-1.293544
H	5.133439	1.116785	-2.171578
H	3.660825	1.617876	1.321450
H	5.133581	1.116508	2.172225
H	5.095177	2.653437	1.294401
H	7.344792	3.005101	0.000336
H	9.777772	2.501579	0.000276
H	10.568366	0.162280	0.000140
H	8.971492	-1.717947	0.000085

*Z*2 (S_0)

C	9.127535	-0.655680	0.000125
C	9.212271	0.734983	-0.000035
C	8.065685	1.530097	-0.000146
C	6.843076	0.876921	-0.000090
C	6.740657	-0.508893	0.000071
C	7.883434	-1.288938	0.000179
C	5.280399	-0.909249	0.000098
C	4.951928	-1.708906	1.275966
C	4.951962	-1.709203	-1.275592
N	5.538195	1.423814	-0.000174
C	5.247623	2.844863	-0.000352
C	4.594966	0.460736	-0.000069
C	3.237077	0.760249	-0.000111
C	2.200799	-0.174718	-0.000020
C	0.848410	0.143956	-0.000056
C	-0.134027	-0.842331	0.000035
C	-1.511113	-0.618349	0.000011
C	-2.394405	-1.693060	0.000106
C	-3.790100	-1.835828	0.000115
C	-4.840633	-0.917213	0.000049
N	-6.122863	-1.342721	0.000064
C	-7.035658	-0.261026	0.000025
C	-6.527644	-2.736219	0.000118
C	-4.832632	0.611310	-0.000031
C	-4.194503	1.162668	1.290743
C	-6.313177	0.924808	-0.000032
C	-4.194540	1.162526	-1.290885
C	-6.972068	2.141124	-0.000081
C	-8.368267	2.146111	-0.000068
C	-9.078643	0.947709	-0.000008
C	-8.421747	-0.283468	0.000040
H	10.034905	-1.250076	0.000208
H	10.185765	1.214008	-0.000075
H	8.152135	2.611064	-0.000267
H	7.818665	-2.373377	0.000305
H	3.900337	-2.003728	1.316881
H	5.558707	-2.619085	1.297569
H	5.181481	-1.126316	2.172503
H	5.181526	-1.126816	-2.172259
H	5.558752	-2.619379	-1.296975
H	3.900376	-2.004048	-1.316461
H	6.180244	3.405374	-0.000460
H	4.676919	3.116420	-0.893420
H	4.676976	3.116659	0.892679
H	2.949041	1.807446	-0.000220
H	2.448040	-1.233290	0.000087
H	0.549393	1.190344	-0.000158
H	0.203394	-1.880608	0.000137
H	-1.851536	0.409012	-0.000084
H	-1.891731	-2.661195	0.000193
H	-4.102511	-2.874202	0.000196
H	-6.151205	-3.243287	0.893346
H	-7.613920	-2.795680	0.000045
H	-6.151085	-3.243385	-0.893002
H	-3.160492	0.842059	1.422027
H	-4.217798	2.256459	1.266741
H	-4.762928	0.832080	2.164619
H	-3.160540	0.841883	-1.422176
H	-4.763003	0.831860	-2.164707
H	-4.217813	2.256319	-1.266993
H	-6.418154	3.075544	-0.000126
H	-8.904183	3.089247	-0.000104
H	-10.163448	0.965923	0.000002
H	-8.993638	-1.204762	0.000090

*Z*3 (S_0)

C	-8.927784	1.017774	0.000020
C	-8.347954	2.284467	0.000026
C	-6.961928	2.446080	0.000018
C	-6.190319	1.294444	0.000003
C	-6.752412	0.023862	0.000001
C	-8.127823	-0.126304	0.000008
C	-5.653923	-1.018415	-0.000009
C	-5.745813	-1.877659	-1.276340
C	-5.745808	-1.877675	1.276312
N	-4.782032	1.162270	-0.000011
C	-3.858417	2.278758	-0.000022
C	-4.401864	-0.133688	-0.000008
C	-3.063358	-0.505123	-0.000001

C	-2.600528	-1.825859	-0.000010
C	-1.282748	-2.275929	-0.000006
C	-0.146373	-1.466493	0.000001
C	1.161673	-1.939974	0.000006
C	2.256238	-1.082795	0.000006
C	3.586618	-1.503322	0.000009
C	4.699990	-0.669659	0.000006
N	5.956618	-1.156813	0.000009
C	6.918663	-0.119423	0.000004
C	6.279825	-2.571259	0.000015
C	4.757570	0.860762	-0.000001
C	4.127593	1.452542	-1.275875
C	6.252514	1.100156	-0.000003
C	4.127596	1.452552	1.275870
C	6.970360	2.282984	-0.000009
C	8.364938	2.220937	-0.000009
C	9.017131	0.989676	-0.000001
C	8.302036	-0.208431	0.000006
H	-10.008107	0.920253	0.000026
H	-8.981255	3.165450	0.000038
H	-6.529711	3.440716	0.000027
H	-8.580858	-1.113689	0.000005
H	-4.961970	-2.637708	-1.319513
H	-6.712576	-2.389498	-1.297260
H	-5.672065	-1.255000	-2.172369
H	-5.672054	-1.255027	2.172348
H	-6.712570	-2.389513	1.297230
H	-4.961965	-2.637725	1.319471
H	-4.417823	3.212153	-0.000107
H	-3.226880	2.251555	0.893435
H	-3.226792	2.251454	-0.893414
H	-2.330050	0.294580	0.000015
H	-3.347108	-2.613419	-0.000023
H	-1.128947	-3.351695	-0.000010
H	-0.276538	-0.383943	0.000001
H	1.331779	-3.014893	0.000007
H	2.039711	-0.017411	0.000003
H	3.757254	-2.575853	0.000014
H	5.872614	-3.054409	-0.893056
H	7.360724	-2.695836	0.000007
H	5.872627	-3.054399	0.893097
H	3.048551	1.283632	-1.317287
H	4.302304	2.532449	-1.297319
H	4.578434	1.017775	-2.172284
H	3.048553	1.283640	1.317286
H	4.578439	1.017793	2.172280
H	4.302304	2.532460	1.297304
H	6.462890	3.243570	-0.000015
H	8.945407	3.137273	-0.000014
H	10.101558	0.955986	-0.000001
H	8.827868	-1.156808	0.000012

*Z*4 (S_0)

C	8.936172	0.300367	-0.000095
C	8.660559	1.665970	-0.000049
C	7.348018	2.139635	0.000006
C	6.334082	1.194429	0.000012
C	6.591536	-0.171149	-0.000035
C	7.896373	-0.631197	-0.000088
C	5.283533	-0.934145	-0.000019
C	5.172723	-1.791735	1.275653
C	5.172655	-1.791674	-1.275726
N	4.932855	1.387657	0.000061
C	4.286763	2.685352	0.000127
C	4.267988	0.213282	0.000036
C	2.880382	0.152759	0.000054
C	2.119524	-1.020031	0.000045
C	0.733043	-1.064908	0.000052
C	0.052864	-2.284693	0.000044
C	-1.323105	-2.523494	0.000041
C	-2.298608	-1.532436	0.000031
C	-3.670078	-1.785174	0.000025
C	-4.667616	-0.815420	0.000007
N	-5.976366	-1.136551	-0.000003
C	-6.796667	0.016006	-0.000017
C	-6.479245	-2.497648	0.000001
C	-4.527284	0.709794	-0.000003
C	-3.826406	1.216157	1.275853
C	-5.979009	1.139671	-0.000018
C	-3.826386	1.216138	-1.275856
C	-6.538700	2.405040	-0.000033
C	-7.929627	2.523020	-0.000046
C	-8.734879	1.385926	-0.000044
C	-8.180006	0.105803	-0.000029
H	9.965802	-0.040955	-0.000138
H	9.478206	2.379179	-0.000057
H	7.153863	3.206568	0.000037
H	8.112292	-1.695900	-0.000126
H	4.233134	-2.348439	1.316630
H	5.993964	-2.514357	1.297356
H	5.243961	-1.169636	2.172228
H	5.243846	-1.169532	-2.172275
H	5.993894	-2.514296	-1.297507
H	4.233063	-2.348375	-1.316681
H	3.665142	2.801342	0.893190
H	5.043601	3.467220	0.000207
H	3.665196	2.801466	-0.892958
H	2.331839	1.090250	0.000073
H	2.633298	-1.977820	0.000030
H	0.185135	-0.125176	0.000064
H	0.682535	-3.174685	0.000040
H	-1.650375	-3.559906	0.000041
H	-1.963700	-0.500034	0.000025
H	-3.975044	-2.827637	0.000032
H	-6.137768	-3.029228	0.893073
H	-7.567196	-2.481438	-0.000030
H	-6.137719	-3.029250	-0.893040
H	-2.777527	0.911188	1.317033
H	-3.862598	2.309535	1.297749

H	-4.328270	0.841869	2.172429
H	-2.777506	0.911170	-1.317014
H	-4.328235	0.841835	-2.172435
H	-3.862579	2.309516	-1.297770
H	-5.911872	3.292361	-0.000035
H	-8.387330	3.506436	-0.000058
H	-9.814616	1.492142	-0.000054
H	-8.823449	-0.767031	-0.000027

*Z*1 – Transition State (S_0)

C	0.012326	-0.105132	-0.019080
C	0.003741	0.031760	1.366144
C	1.191806	0.122637	2.093966
C	2.382977	0.071296	1.384751
C	2.406569	-0.065192	-0.000544
C	1.224514	-0.154384	-0.712051
C	3.843238	-0.090315	-0.484177
C	4.102321	1.099645	-1.426427
C	4.142572	-1.434085	-1.173971
C	4.610791	0.053619	0.837500
N	3.704434	0.138986	1.858646
C	4.079580	0.280899	3.246011
C	5.963104	0.098592	1.051342
C	6.972874	0.015093	0.053150
C	8.313964	0.064256	0.299506
C	9.283727	-0.027190	-0.739643
C	10.634556	0.011703	-0.572447
C	11.540577	-0.092992	-1.674254
C	12.891700	-0.057590	-1.589271
C	13.761185	-0.212236	-2.751903
N	14.234018	0.782015	-3.456018
C	14.280156	-1.528366	-3.293328
C	15.085093	-2.252002	-2.194374
C	15.142031	-1.063555	-4.439704
C	13.096719	-2.400305	-3.759646
C	13.965871	2.199050	-3.239246
C	15.087594	0.322584	-4.505813
C	15.765278	1.076781	-5.448079
C	16.536718	0.367535	-6.367342
C	16.608065	-1.026341	-6.323731
C	15.911104	-1.756883	-5.360249
H	-0.924514	-0.173676	-0.561779
H	-0.942335	0.069268	1.896788
H	1.162111	0.229151	3.172984
H	1.234342	-0.261171	-1.793594
H	5.137025	1.124960	-1.778836
H	3.447920	1.023888	-2.300808
H	3.888729	2.046692	-0.922865
H	3.488199	-1.553202	-2.043517
H	5.178334	-1.495680	-1.518615
H	3.958077	-2.269415	-0.492614
H	3.184381	0.321726	3.864585
H	4.687241	-0.571329	3.569767
H	4.650570	1.203433	3.398850
H	6.314766	0.207503	2.073692
H	6.666742	-0.096738	-0.984543
H	8.668509	0.175429	1.322930
H	8.897962	-0.139640	-1.755450
H	11.053222	0.122412	0.426056
H	11.088834	-0.206774	-2.661623
H	13.395856	0.037211	-0.628537
H	14.437815	-2.495099	-1.347021
H	15.487674	-3.183685	-2.601195
H	15.922995	-1.642954	-1.842825
H	12.445037	-2.642874	-2.916078
H	12.509513	-1.898787	-4.534773
H	13.485933	-3.332629	-4.177881
H	13.273336	2.301913	-2.405429
H	14.906847	2.708016	-3.019122
H	13.526005	2.617588	-4.147003
H	15.708960	2.159575	-5.479797
H	17.089315	0.909529	-7.126987
H	17.217412	-1.549854	-7.052765
H	15.974516	-2.840356	-5.336085

*Z*2 – Transition State (S_0)

C	0.013008	0.187469	0.067173
C	0.019807	-0.009914	1.447850
C	1.215109	-0.149810	2.151655
C	2.386585	-0.083045	1.414166
C	2.400312	0.112458	0.040030
C	1.207910	0.250314	-0.650054
C	3.829501	0.135330	-0.453270
C	4.153475	1.506127	-1.082546
C	4.069458	-1.022949	-1.443648
C	4.594105	-0.076247	0.851831
N	3.732954	-0.190831	1.859575
C	4.108710	-0.401090	3.251817
C	5.990625	-0.153231	1.060476
C	6.942822	-0.045009	0.079820
C	8.339992	-0.127102	0.316455
C	9.243033	-0.014551	-0.697418
C	10.667801	-0.083831	-0.539047
C	11.507712	0.050620	-1.587033
C	12.974276	-0.060936	-1.508136
C	13.839876	0.963244	-1.368724
N	15.212775	0.800111	-1.319810
C	13.526460	2.460612	-1.235659
C	12.665698	2.975741	-2.397155
C	14.919496	3.060624	-1.263167
C	12.850577	2.762851	0.114773
C	15.851642	-0.485052	-1.423645
C	15.867373	2.037728	-1.301405
C	17.227669	2.311692	-1.316258
C	17.620521	3.652169	-1.286155
C	16.685105	4.680921	-1.243635
C	15.319180	4.382662	-1.230175
H	-0.932364	0.293043	-0.453938

H	-0.919383	-0.056204	1.988427
H	1.201387	-0.302932	3.224877
H	1.197269	0.403998	-1.725055
H	5.183982	1.560984	-1.441192
H	3.490615	1.671012	-1.936591
H	3.992061	2.316467	-0.366281
H	3.405527	-0.898759	-2.303718
H	5.097642	-1.042600	-1.812525
H	3.848601	-1.988902	-0.981172
H	3.209943	-0.451809	3.861786
H	4.656771	-1.340720	3.355438
H	4.724898	0.429652	3.604114
H	6.338207	-0.311182	2.076951
H	6.639531	0.113323	-0.951470
H	8.687863	-0.284776	1.335223
H	8.870908	0.143244	-1.710925
H	11.071390	-0.239617	0.459107
H	11.058879	0.197162	-2.574467
H	13.356781	-1.074643	-1.611599
H	11.656575	2.551205	-2.362251
H	12.567495	4.064464	-2.333532
H	13.117474	2.729596	-3.362271
H	11.851585	2.318964	0.161990
H	13.448148	2.373632	0.944367
H	12.752342	3.845784	0.245174
H	15.686126	-0.935941	-2.410972
H	16.924365	-0.377243	-1.264481
H	15.461075	-1.165022	-0.657425
H	17.974463	1.525699	-1.355580
H	18.679849	3.889278	-1.299263
H	17.015151	5.714186	-1.222967
H	14.583793	5.182725	-1.197025

*Z*3 – Transition State (S_0)

C	-0.023705	0.039647	0.241742
C	0.061689	-0.186659	1.616290
C	1.295685	-0.296931	2.254032
C	2.421202	-0.171081	1.455043
C	2.358443	0.056851	0.087910
C	1.126673	0.163967	-0.536565
C	3.758208	0.146682	-0.473134
C	4.003223	1.541524	-1.087532
C	3.997719	-0.983246	-1.498263
C	4.590153	-0.059648	0.785712
N	3.797117	-0.238935	1.825132
C	4.247261	-0.474813	3.196036
C	6.019011	-0.063464	0.933656
C	6.905554	0.090352	-0.073799
C	8.363443	0.064816	0.130172
C	9.106726	1.169897	0.325276
C	10.534252	1.188733	0.520830
C	11.228345	2.337318	0.711536
C	12.645805	2.410600	0.902520
C	13.377537	3.546135	1.088932
N	14.741384	3.526439	1.260975
C	12.904129	5.006286	1.147731
C	12.276403	5.456626	-0.183940
C	14.212773	5.737916	1.376915
C	11.951140	5.250847	2.331717
C	15.522020	2.313752	1.255845
C	15.257994	4.818562	1.434864
C	16.570574	5.221097	1.637354
C	16.812860	6.588768	1.781977
C	15.777983	7.517545	1.725788
C	14.463481	7.089502	1.521209
H	-0.998853	0.120776	-0.226237
H	-0.846015	-0.278282	2.202686
H	1.344448	-0.469405	3.323198
H	1.052824	0.341180	-1.605245
H	5.014558	1.639012	-1.487986
H	3.297669	1.693978	-1.908770
H	3.841547	2.332346	-0.349751
H	3.287065	-0.868000	-2.321175
H	5.006206	-0.949631	-1.915756
H	3.838494	-1.966726	-1.047491
H	3.384045	-0.687354	3.821726
H	4.918270	-1.334945	3.224074
H	4.757008	0.411415	3.580003
H	6.418561	-0.192155	1.934921
H	6.542325	0.222831	-1.092675
H	8.832187	-0.918714	0.093260
H	8.604405	2.138867	0.343747
H	11.059096	0.234556	0.506956
H	10.660046	3.264896	0.715798
H	13.171449	1.459416	0.891041
H	11.340538	4.931176	-0.393318
H	12.062304	6.529787	-0.145663
H	12.963071	5.276347	-1.015839
H	11.003104	4.719551	2.210055
H	12.408406	4.924651	3.270178
H	11.732565	6.320704	2.413233
H	15.415529	1.789468	0.299122
H	16.574216	2.554082	1.402002
H	15.203185	1.644116	2.063054
H	17.392761	4.515070	1.684853
H	17.831608	6.928138	1.941155
H	15.991978	8.574898	1.840785
H	13.652207	7.811604	1.476380

*Z*4 – Transition State (S_0)

C	0.025226	0.240144	0.160713
C	0.057069	0.002014	1.534972
C	1.263748	-0.181051	2.208274
C	2.419462	-0.114359	1.446290
C	2.408839	0.121389	0.078744
C	1.204801	0.301910	-0.581075
C	3.827298	0.131735	-0.443420
C	4.166616	1.514021	-1.039728

C	4.025745	-1.002488	-1.470957
C	4.612527	-0.131828	0.838005
N	3.774812	-0.260506	1.858063
C	4.174680	-0.520159	3.237054
C	6.018403	-0.240853	1.016077
C	6.945512	-0.124332	0.022638
C	8.358452	-0.238981	0.231823
C	9.233109	-0.114466	-0.789916
C	10.694505	-0.239475	-0.655742
C	11.516858	0.808491	-0.464423
C	12.949090	0.705861	-0.334695
C	13.813648	1.735623	-0.134898
N	15.175476	1.550731	-0.027708
C	13.518979	3.237261	0.009534
C	12.897876	3.823596	-1.271105
C	14.913311	3.799850	0.211727
C	12.648978	3.533595	1.244126
C	15.805799	0.258388	-0.125244
C	15.844852	2.763827	0.179780
C	17.202602	3.003448	0.339658
C	17.608937	4.325374	0.534264
C	16.688836	5.368733	0.567791
C	15.326103	5.104608	0.404821
H	-0.928979	0.378700	-0.336019
H	-0.871312	-0.042436	2.093946
H	1.269704	-0.365307	3.276606
H	1.173863	0.487412	-1.650565
H	5.190398	1.558577	-1.418045
H	3.490093	1.715211	-1.874938
H	4.035245	2.306774	-0.298089
H	3.346389	-0.840821	-2.312415
H	5.045241	-1.029354	-1.862224
H	3.796207	-1.977341	-1.032005
H	3.287012	-0.575461	3.862397
H	4.708265	-1.471292	3.299549
H	4.810823	0.289674	3.601366
H	6.382033	-0.433580	2.020914
H	6.629497	0.067393	-0.999025
H	8.724134	-0.428638	1.238501
H	8.823717	0.074169	-1.787546
H	11.097633	-1.249563	-0.735303
H	11.065533	1.795411	-0.399789
H	13.354486	-0.299821	-0.409182
H	11.896826	3.425816	-1.460384
H	12.817810	4.911672	-1.177486
H	13.523536	3.602412	-2.140389
H	11.639905	3.124145	1.141613
H	13.100641	3.110059	2.145736
H	12.562483	4.616337	1.383152
H	15.593778	-0.203618	-1.096724
H	16.884897	0.367295	-0.024456
H	15.448943	-0.409543	0.667837
H	17.937880	2.206151	0.317443
H	18.666115	4.536767	0.661349
H	17.029381	6.387480	0.720078
H	14.603600	5.916492	0.429698

all-*E* (T_1)

C	-9.317520	1.215979	0.000401
C	-9.580031	-0.153464	0.000341
C	-8.545643	-1.087706	0.000255
C	-7.246745	-0.596859	0.000231
C	-6.967779	0.767376	0.000289
C	-8.003078	1.685425	0.000374
C	-5.469504	0.982481	0.000241
C	-5.038475	1.736855	1.273757
C	-5.038576	1.736980	-1.273235
C	-4.965642	-0.458872	0.000151
N	-6.029740	-1.305866	0.000151
C	-5.923623	-2.751253	0.000078
C	-3.653671	-0.923823	0.000078
C	-2.484471	-0.155358	0.000073
C	-1.218226	-0.747537	-0.000001
C	0.000000	-0.066935	-0.000011
C	1.218226	-0.747537	-0.000084
C	2.484471	-0.155358	-0.000100
C	3.653671	-0.923823	-0.000172
C	4.965642	-0.458872	-0.000196
N	6.029740	-1.305866	-0.000265
C	5.469504	0.982481	-0.000155
C	5.038481	1.736970	-1.273604
C	6.967779	0.767376	-0.000216
C	5.038571	1.736866	1.273387
C	5.923623	-2.751253	-0.000319
C	7.246745	-0.596859	-0.000279
C	8.545643	-1.087705	-0.000343
C	9.580031	-0.153464	-0.000342
C	9.317520	1.215979	-0.000279
C	8.003078	1.685425	-0.000215
H	-10.141829	1.921124	0.000468
H	-10.607003	-0.503453	0.000362
H	-8.769977	-2.148668	0.000209
H	-7.800431	2.752629	0.000420
H	-3.955281	1.875141	1.316937
H	-5.506803	2.725805	1.286934
H	-5.353599	1.198627	2.172100
H	-5.506910	2.725929	-1.286279
H	-3.955387	1.875276	-1.316487
H	-5.353768	1.198838	-2.171606
H	-6.919761	-3.188992	0.000110
H	-5.393056	-3.094488	-0.893295
H	-5.392965	-3.094570	0.893367
H	-3.514660	-2.002907	0.000019
H	-2.535950	0.928184	0.000129
H	-1.179463	-1.838468	-0.000055
H	0.000000	1.021045	0.000040
H	1.179463	-1.838468	-0.000133
H	2.535950	0.928184	-0.000054
H	3.514660	-2.002907	-0.000214

H	3.955288	1.875259	-1.316778
H	5.506809	2.725921	-1.286690
H	5.353609	1.198823	-2.171994
H	3.955381	1.875157	1.316646
H	5.353758	1.198642	2.171711
H	5.506905	2.725813	1.286523
H	5.392986	-3.094494	-0.893649
H	6.919761	-3.188992	-0.000363
H	5.393034	-3.094564	0.893013
H	8.769977	-2.148668	-0.000393
H	10.607003	-0.503453	-0.000392
H	10.141829	1.921124	-0.000280
H	7.800431	2.752629	-0.000166

all-*E* S_1)

C	9.165444	-1.474593	0.000090
C	9.534696	-0.128292	-0.000022
C	8.579506	0.884474	-0.000090
C	7.241886	0.499165	-0.000041
C	6.858118	-0.843302	0.000068
C	7.817785	-1.839170	0.000136
C	5.348300	-0.938773	0.000091
C	4.859697	-1.660029	1.273971
C	4.859672	-1.660239	-1.273661
C	4.958866	0.536547	-0.000027
N	6.091015	1.295043	-0.000089
C	6.095845	2.743621	-0.000195
C	3.690915	1.107767	-0.000078
C	2.465484	0.425369	-0.000007
C	1.233170	1.079384	-0.000075
C	0.000012	0.419917	-0.000004
C	-1.233173	1.079333	-0.000074
C	-2.465461	0.425268	-0.000004
C	-3.690919	1.107617	-0.000076
C	-4.958848	0.536348	-0.000021
N	-6.091025	1.294802	-0.000093
C	-5.348227	-0.938986	0.000115
C	-4.859575	-1.660449	-1.273630
C	-6.858048	-0.843572	0.000095
C	-4.859593	-1.660210	1.274002
C	-6.095909	2.743380	-0.000224
C	-7.241866	0.498881	-0.000030
C	-8.579500	0.884141	-0.000078
C	-9.534653	-0.128661	0.000005
C	-9.165351	-1.474948	0.000131
C	-7.817678	-1.839475	0.000177
H	9.932116	-2.242148	0.000140
H	10.586420	0.138882	-0.000059
H	8.887263	1.924314	-0.000181
H	7.532199	-2.887364	0.000221
H	3.769108	-1.719041	1.314627
H	5.254783	-2.680556	1.287553
H	5.211053	-1.145939	2.172859
H	5.254752	-2.680770	-1.287079
H	3.769082	-1.719251	-1.314289
H	5.211017	-1.146302	-2.172640
H	7.122461	3.104481	-0.000159
H	5.590939	3.126409	-0.893129
H	5.590838	3.126534	0.892628
H	3.632483	2.193540	-0.000186
H	2.452773	-0.660704	0.000109
H	1.229600	2.169228	-0.000191
H	0.000035	-0.670189	0.000113
H	-1.229647	2.169178	-0.000190
H	-2.452706	-0.660805	0.000112
H	-3.632530	2.193392	-0.000187
H	-3.768983	-1.719422	-1.314259
H	-5.254619	-2.680994	-1.287036
H	-5.210939	-1.146533	-2.172614
H	-3.769001	-1.719178	1.314656
H	-5.210968	-1.146125	2.172885
H	-5.254639	-2.680752	1.287595
H	-5.590988	3.126170	-0.893148
H	-7.122538	3.104201	-0.000231
H	-5.590946	3.126329	0.892608
H	-8.887295	1.923969	-0.000177
H	-10.586387	0.138474	-0.000030
H	-9.931994	-2.242532	0.000194
H	-7.532054	-2.887659	0.000275

*Z*1 (S_1)

C	9.463573	0.724335	-0.332946
C	8.915141	1.991612	-0.130540
C	7.547954	2.163838	0.073151
C	6.756278	1.019853	0.069028
C	7.288748	-0.252667	-0.141678
C	8.648193	-0.409503	-0.340510
C	6.175903	-1.270895	-0.095714
C	6.358230	-2.241720	1.089995
C	6.068592	-2.051920	-1.418693
C	4.957454	-0.377982	0.123032
N	5.369173	0.912260	0.257192
C	4.551287	2.036420	0.665070
C	3.660509	-0.879147	0.158147
C	2.424820	-0.214478	0.113170
C	1.205684	-0.893295	0.167081
C	-0.040970	-0.265414	0.092045
C	-1.260078	-0.949804	0.140010
C	-2.505032	-0.326213	0.058458
C	-3.717108	-1.030398	0.110761
C	-4.995136	-0.487271	0.035866
N	-6.113578	-1.263141	0.098911
C	-7.278247	-0.493700	-0.000131
C	-6.093226	-2.703767	0.249911
C	-5.410597	0.972579	-0.121888
C	-4.955550	1.830631	1.077438
C	-6.918223	0.848625	-0.133371
C	-4.914869	1.568148	-1.456540

C	-7.894992	1.820663	-0.250115
C	-9.235852	1.432319	-0.231932
C	-9.581391	0.086380	-0.098289
C	-8.608714	-0.902547	0.020142
H	10.532052	0.620680	-0.489829
H	9.562012	2.862819	-0.136181
H	7.134751	3.157028	0.211790
H	9.076965	-1.394516	-0.501298
H	5.523373	-2.947163	1.150605
H	7.278937	-2.817801	0.953773
H	6.428902	-1.700174	2.037465
H	5.927965	-1.375361	-2.266211
H	6.986198	-2.625107	-1.584535
H	5.230548	-2.755372	-1.391325
H	5.187718	2.783801	1.140228
H	4.043330	2.495456	-0.190290
H	3.813089	1.703251	1.397292
H	3.610294	-1.966183	0.168727
H	2.383020	0.863890	-0.006346
H	1.226332	-1.977601	0.273582
H	-0.065418	0.819466	-0.012746
H	-1.233409	-2.034038	0.246744
H	-2.513742	0.754492	-0.049508
H	-3.637988	-2.109240	0.220685
H	-5.590392	-2.981968	1.181822
H	-7.113461	-3.081109	0.279950
H	-5.573157	-3.168541	-0.594082
H	-3.866959	1.914459	1.127504
H	-5.368880	2.839292	0.979742
H	-5.312385	1.405302	2.019515
H	-3.824993	1.643619	-1.488338
H	-5.242963	0.957547	-2.302264
H	-5.327880	2.573998	-1.580871
H	-7.627871	2.868529	-0.354428
H	-10.015741	2.180959	-0.322472
H	-10.628114	-0.199494	-0.086110
H	-8.898273	-1.942526	0.122587

*Z*2 (S_1)

C	8.401205	0.012268	-0.056319
C	7.011555	0.111596	-0.050958
C	6.192000	-1.017804	-0.042200
C	6.749272	-2.283629	-0.038517
C	8.139607	-2.404852	-0.043758
C	8.949127	-1.266856	-0.052549
C	4.742108	-0.589212	-0.038147
C	4.873176	0.930336	-0.046201
N	6.203449	1.251705	-0.053000
C	3.909435	1.941399	-0.047623
C	2.501467	1.950528	-0.042087
C	1.541847	0.934088	-0.032551
C	0.168243	1.198997	-0.028782
C	-0.813298	0.203112	-0.019372
C	-2.183147	0.465889	-0.015637
C	-3.156475	-0.543043	-0.006236
C	-4.535741	-0.361744	-0.001802
C	-5.333453	0.939399	-0.005624
C	-6.751862	0.413462	0.003021
C	-6.733278	-0.982061	0.010419
N	-5.400580	-1.413026	0.007318
C	-7.957499	1.091171	0.004284
C	-9.143013	0.354017	0.013037
C	-9.109241	-1.041422	0.020367
C	-7.902696	-1.736362	0.019196
C	-5.068388	1.777342	1.262883
C	-5.078943	1.763482	-1.285332
C	-4.988132	-2.801778	0.013099
C	6.721131	2.605657	-0.061395
C	4.068564	-1.094755	1.256504
C	4.057408	-1.107589	-1.321799
H	-10.098134	0.868450	0.014158
H	-10.039470	-1.600070	0.027118
H	-7.898107	-2.820755	0.024984
H	-7.985748	2.177154	-0.001422
H	-4.041190	2.148945	1.297949
H	-5.741039	2.640489	1.274480
H	-5.253255	1.188541	2.165607
H	-5.270897	1.164797	-2.180040
H	-5.752009	2.626244	-1.300908
H	-4.052242	2.135057	-1.332806
H	-5.867274	-3.442977	0.020017
H	-4.397999	-3.027735	-0.881000
H	-4.390890	-3.018197	0.904839
H	-2.786291	-1.565388	-0.001996
H	-2.488028	1.508283	-0.020383
H	-0.488515	-0.837343	-0.014705
H	-0.156109	2.239742	-0.033478
H	1.835937	-0.108405	-0.027676
H	2.085740	2.957432	-0.046092
H	4.322988	2.944960	-0.054666
H	6.390047	3.147989	0.829978
H	7.808678	2.577457	-0.064559
H	6.384546	3.139112	-0.956069
H	3.058478	-0.703359	1.386315
H	4.016185	-2.187990	1.236790
H	4.657133	-0.799251	2.129516
H	3.046215	-0.717456	-1.446737
H	4.638380	-0.820828	-2.202777
H	4.005234	-2.200570	-1.290743
H	6.119857	-3.169016	-0.031708
H	8.596055	-3.389010	-0.041010
H	10.028573	-1.377582	-0.056569
H	9.049561	0.881264	-0.063207

*Z*3 (S_1)

C	8.355462	-0.320562	0.072493
C	6.972473	-0.167603	0.058609
C	6.366229	1.089395	0.078662

C	7.142429	2.233577	0.113945
C	8.532309	2.103031	0.128692
C	9.124799	0.839473	0.108011
C	4.862225	0.926598	0.055865
C	4.729607	-0.593393	0.019974
N	5.972840	-1.147917	0.024471
C	3.576541	-1.371174	-0.013477
C	2.255248	-0.905930	-0.020624
C	1.148986	-1.758180	-0.055237
C	-0.174620	-1.316523	-0.062587
C	-1.271574	-2.192954	-0.097479
C	-2.623881	-1.845793	-0.106548
C	-3.126793	-0.531752	-0.081430
C	-4.467416	-0.166621	-0.089099
C	-5.708990	-1.053964	-0.125053
C	-6.810842	-0.017650	-0.113608
C	-6.251709	1.260582	-0.076460
N	-4.856656	1.139241	-0.062729
C	-8.184905	-0.173038	-0.133769
C	-8.990668	0.966886	-0.116193
C	-8.417603	2.239128	-0.078954
C	-7.035996	2.410121	-0.058380
C	-5.778617	-1.889550	-1.420661
C	-5.809111	-1.949963	1.127479
C	-3.938160	2.257714	-0.025371
C	6.223736	-2.574713	-0.003095
C	4.274165	1.582373	-1.211117
C	4.241960	1.524817	1.335969
H	-10.070540	0.864356	-0.131580
H	-9.057779	3.115154	-0.065697
H	-6.610477	3.407190	-0.029449
H	-8.632892	-1.162243	-0.162806
H	-4.976860	-2.630184	-1.471285
H	-6.733905	-2.422094	-1.457344
H	-5.711827	-1.247566	-2.303411
H	-5.764058	-1.350526	2.040988
H	-6.764587	-2.483307	1.115913
H	-5.008231	-2.692444	1.162249
H	-4.499257	3.189976	-0.010298
H	-3.315744	2.210119	0.874519
H	-3.294190	2.251994	-0.911220
H	-2.402678	0.276680	-0.053080
H	-3.326537	-2.670633	-0.135175
H	-1.043524	-3.256589	-0.119621
H	-0.345365	-0.241587	-0.039860
H	1.329865	-2.832643	-0.077670
H	2.059666	0.162395	0.001276
H	3.703111	-2.450781	-0.036846
H	5.808272	-3.017412	-0.914167
H	7.296462	-2.756500	0.013483
H	5.774478	-3.057307	0.870856
H	3.190086	1.455130	-1.268747
H	4.489722	2.655317	-1.198099
H	4.719567	1.155821	-2.114093
H	3.156919	1.395086	1.360815
H	4.665253	1.058187	2.229710
H	4.456584	2.597270	1.376467
H	6.682900	3.217900	0.129952
H	9.157299	2.989328	0.156249
H	10.206516	0.754515	0.119643
H	8.835487	-1.292781	0.056507

*Z*4 (S_1)

C	8.903461	0.476439	0.017841
C	8.553522	1.827462	-0.000804
C	7.220422	2.229678	-0.010928
C	6.253464	1.229260	-0.001727
C	6.588362	-0.125208	0.017034
C	7.916541	-0.510968	0.026913
C	5.326422	-0.959738	0.023199
C	5.257371	-1.811614	1.307992
C	5.266388	-1.843966	-1.240034
N	4.857212	1.346191	-0.008497
C	4.140674	2.604788	-0.027895
C	4.253712	0.126195	0.005536
C	2.870283	-0.010485	0.003141
C	2.150534	-1.215565	0.016116
C	0.759915	-1.269888	0.013284
C	0.033491	-2.472258	0.026012
C	-1.359980	-2.594706	0.024485
C	-2.284747	-1.542418	0.010665
C	-3.668087	-1.756928	0.010037
C	-4.656067	-0.776165	-0.002837
N	-5.982005	-1.087061	-0.002479
C	-6.778899	0.063397	-0.016397
C	-6.496993	-2.441408	0.010693
C	-4.500421	0.741643	-0.018787
C	-3.792135	1.258897	1.251300
C	-5.947010	1.184460	-0.026683
C	-3.786428	1.231560	-1.296497
C	-6.493911	2.454644	-0.041373
C	-7.883463	2.588555	-0.045624
C	-8.703239	1.458734	-0.035155
C	-8.166341	0.174307	-0.020327
H	9.950471	0.192573	0.025386
H	9.332406	2.582933	-0.007578
H	6.969465	3.284582	-0.025220
H	8.190957	-1.561985	0.041497
H	4.342304	-2.407457	1.352678
H	6.109267	-2.498007	1.334070
H	5.301377	-1.179231	2.199029
H	5.316665	-1.234390	-2.146502
H	6.118437	-2.530659	-1.242700
H	4.351643	-2.440896	-1.276017
H	3.511089	2.698795	0.863027
H	4.851544	3.428606	-0.039343
H	3.513448	2.672292	-0.922840
H	2.277350	0.900955	-0.009854
H	2.685106	-2.160988	0.029092

H	0.222218	-0.323071	0.000444
H	0.608088	-3.396110	0.038185
H	-1.763097	-3.605428	0.035208
H	-1.909966	-0.525053	0.000185
H	-3.993445	-2.794411	0.020776
H	-6.162221	-2.967725	0.910398
H	-7.584893	-2.417185	0.006946
H	-6.156626	-2.987127	-0.875244
H	-2.750908	0.929374	1.299510
H	-3.802712	2.353391	1.252638
H	-4.304537	0.911689	2.152673
H	-2.744892	0.901441	-1.332731
H	-4.294558	0.864739	-2.192507
H	-3.797272	2.325764	-1.321579
H	-5.857179	3.334930	-0.049457
H	-8.330544	3.576923	-0.057116
H	-9.781639	1.579196	-0.038576
H	-8.820848	-0.690223	-0.012298

*Z*1 – Twisted Structure (S_1)

C	-7.976196	-1.069201	-1.431158
C	-6.938794	-0.472702	-0.728385
C	-7.060888	-0.123057	0.616831
C	-8.250538	-0.352152	1.283755
C	-9.313052	-0.942680	0.593499
C	-9.171238	-1.295698	-0.745841
N	-5.659621	-0.130226	-1.188721
C	-4.876658	0.265726	-0.110593
C	-5.766616	0.502361	1.105329
C	-3.629704	0.989471	-0.324093
C	-2.390593	0.407994	-0.176100
C	-1.189122	1.104747	-0.337347
C	0.066810	0.501159	-0.184470
C	1.269866	1.160454	-0.334669
C	2.504533	0.508746	-0.166215
C	3.728162	1.130666	-0.304931
C	4.980071	0.509812	-0.142102
N	6.121533	1.185092	-0.293658
C	7.254156	0.353236	-0.081837
C	6.804107	-0.924196	0.222198
C	5.291644	-0.943969	0.212557
C	8.599655	0.677799	-0.147121
C	9.508967	-0.347739	0.109421
C	9.076043	-1.637076	0.416554
C	7.714549	-1.935895	0.475410
C	6.205762	2.597933	-0.633446
C	4.756386	-1.312274	1.611151
C	4.779864	-1.913558	-0.871875
C	-5.208139	-0.197583	2.354596
C	-5.968732	2.006001	1.394409
C	-5.142200	-0.602084	-2.449922
H	-10.251002	-1.130997	1.105001
H	-10.000874	-1.759556	-1.269843
H	-7.872536	-1.356157	-2.472428
H	-8.359118	-0.082797	2.330945
H	-4.233359	0.221733	2.627774
H	-5.882605	-0.059943	3.206902
H	-5.089956	-1.270503	2.178690
H	-6.694892	2.141455	2.202566
H	-5.029471	2.476696	1.706290
H	-6.347016	2.526997	0.509139
H	-4.978984	-1.688204	-2.440021
H	-5.845177	-0.358772	-3.250909
H	-4.194933	-0.105354	-2.664608
H	-3.653406	2.058898	-0.570491
H	-2.352095	-0.648856	0.082838
H	-1.228130	2.162931	-0.589422
H	0.087561	-0.558338	0.071618
H	1.268484	2.218322	-0.589784
H	2.463445	-0.546380	0.088547
H	3.720434	2.186019	-0.560197
H	3.664325	-1.323053	1.643915
H	5.111253	-2.311875	1.877318
H	5.118361	-0.610917	2.367996
H	3.688510	-1.944300	-0.914347
H	5.156930	-1.633639	-1.859413
H	5.136493	-2.922356	-0.645780
H	7.251041	2.892173	-0.691010
H	5.736601	2.780884	-1.603452
H	5.714531	3.200117	0.135008
H	8.954538	1.674377	-0.384476
H	10.571410	-0.133429	0.068071
H	9.805982	-2.415213	0.611543
H	7.381652	-2.941468	0.715038

*Z*2 – Twisted Structure (S_1)

C	-9.011758	0.501810	0.905231
C	-9.159209	0.732508	-0.459465
C	-8.067620	0.670031	-1.327891
C	-6.829753	0.369149	-0.779553
C	-6.667272	0.136104	0.583028
C	-7.754546	0.200357	1.434660
C	-5.211145	-0.166349	0.877552
C	-5.075094	-1.585773	1.458859
C	-4.635725	0.893703	1.834569
C	-4.592044	-0.064736	-0.522261
N	-5.581367	0.243989	-1.415244
C	-5.353774	0.411702	-2.831122
C	-3.285293	-0.239494	-0.903475
C	-2.199568	-0.563128	-0.053513
C	-0.904390	-0.739184	-0.485903
C	0.160395	-1.067512	0.365877
C	1.477217	-1.258563	-0.067151
C	2.540010	-1.571498	0.749411
C	3.884090	-1.823316	0.240088
C	4.890539	-0.871637	0.136090
N	6.119739	-1.176161	-0.339041
C	4.844745	0.601230	0.516341

C	3.831570	1.376776	-0.353425
C	6.255270	1.026420	0.196934
C	4.531123	0.788054	2.014445
C	6.525739	-2.494432	-0.806925
C	6.970237	-0.058561	-0.311483
C	8.304384	0.032317	-0.699918
C	8.914470	1.272082	-0.561771
C	8.211865	2.371444	-0.055140
C	6.876473	2.256533	0.327663
H	-9.875461	0.555866	1.559304
H	-10.139506	0.965794	-0.862504
H	-8.204629	0.853071	-2.388183
H	-7.636482	0.019343	2.499702
H	-4.032804	-1.846958	1.659913
H	-5.629307	-1.650643	2.400590
H	-5.487398	-2.328085	0.769624
H	-5.183294	0.867585	2.782049
H	-3.578172	0.719161	2.049460
H	-4.738591	1.896234	1.409683
H	-6.293453	0.649840	-3.326862
H	-4.647400	1.228892	-3.014035
H	-4.953609	-0.509400	-3.268916
H	-3.047137	-0.125909	-1.957948
H	-2.383276	-0.687435	1.010198
H	-0.692926	-0.623861	-1.549189
H	-0.050808	-1.186371	1.428287
H	1.667862	-1.139846	-1.134165
H	2.376152	-1.741549	1.812948
H	4.137493	-2.838811	-0.068970
H	2.812524	1.059414	-0.129493
H	3.921502	2.444839	-0.135278
H	4.027885	1.229665	-1.419587
H	3.510078	0.472245	2.234908
H	5.225891	0.223257	2.642730
H	4.624488	1.847451	2.269866
H	5.852489	-2.830632	-1.597648
H	7.534737	-2.439767	-1.207742
H	6.509482	-3.205985	0.021817
H	8.861772	-0.811086	-1.090522
H	9.953016	1.387394	-0.851000
H	8.716808	3.326343	0.041056
H	6.339566	3.114195	0.720005

*Z*3 – Twisted Structure (S_1)

C	7.677797	-1.196787	1.587492
C	6.655792	-0.598060	0.864625
C	6.787413	-0.314543	-0.493382
C	7.958229	-0.626890	-1.158772
C	9.001173	-1.229271	-0.450576
C	8.855468	-1.507771	0.905302
N	5.385635	-0.185397	1.297547
C	4.663068	0.360390	0.261410
C	5.515992	0.333811	-1.006018
C	3.371095	0.832362	0.423614
C	2.548575	1.399543	-0.547053
C	1.208778	1.885553	-0.222711
C	0.069133	1.120593	-0.327230
C	-1.204784	1.602443	-0.004582
C	-2.361352	0.821181	-0.107503
C	-3.624785	1.290037	0.205527
C	-4.817530	0.550725	0.113823
N	-5.998723	1.084648	0.435692
C	-7.057344	0.153184	0.262867
C	-6.515550	-1.040236	-0.195038
C	-5.016681	-0.895882	-0.334946
C	-8.414179	0.323722	0.487479
C	-9.237724	-0.771519	0.231484
C	-8.712172	-1.978483	-0.228867
C	-7.341707	-2.122276	-0.446359
C	-6.188823	2.449696	0.904291
C	-4.598143	-1.094045	-1.806014
C	-4.296719	-1.884372	0.604570
C	4.879251	-0.536493	-2.105498
C	5.814952	1.753454	-1.523955
C	4.893449	-0.300540	2.647208
H	9.926293	-1.480228	-0.958241
H	9.670037	-1.976090	1.448455
H	7.585433	-1.423944	2.644132
H	8.068618	-0.407556	-2.217405
H	3.930689	-0.118578	-2.453426
H	5.556381	-0.604153	-2.962953
H	4.693650	-1.549461	-1.736936
H	6.506362	1.700138	-2.370994
H	4.907418	2.263296	-1.858344
H	6.280103	2.360213	-0.741954
H	5.667460	-0.724758	3.284834
H	4.622186	0.684124	3.044383
H	4.014398	-0.954136	2.687967
H	2.945349	0.743518	1.420219
H	2.893943	1.564504	-1.561973
H	1.081167	2.916435	0.123692
H	0.168058	0.092627	-0.671408
H	-1.301444	2.630763	0.339446
H	-2.233092	-0.199385	-0.454665
H	-3.697946	2.317824	0.548223
H	-3.520465	-0.981023	-1.945272
H	-4.874727	-2.103503	-2.122713
H	-5.108450	-0.381314	-2.459740
H	-3.209781	-1.796414	0.538313
H	-4.594503	-1.726424	1.644840
H	-4.569529	-2.905628	0.324583
H	-5.849062	3.159010	0.145541
H	-7.245903	2.621056	1.092634
H	-5.637298	2.608798	1.834192
H	-8.839856	1.254514	0.844935
H	-10.305867	-0.678292	0.394708

H	-9.376912	-2.813867	-0.419756
H	-6.937140	-3.063987	-0.805112

*Z*4 – Twisted Structure (S_1)

C	-8.997562	0.803615	0.754416
C	-8.970900	1.333995	-0.532068
C	-7.809793	1.300413	-1.306792
C	-6.682181	0.718131	-0.746894
C	-6.694203	0.183661	0.538878
C	-7.849215	0.222038	1.297642
C	-5.325511	-0.378998	0.869611
C	-5.426000	-1.893701	1.128488
C	-4.728409	0.359689	2.081327
C	-4.554255	-0.073864	-0.418992
N	-5.400028	0.553375	-1.297951
C	-5.007041	0.976994	-2.619784
C	-3.242829	-0.348618	-0.724465
C	-2.298601	-0.992069	0.103111
C	-0.980462	-1.236834	-0.288614
C	-0.029227	-1.856282	0.491065
C	1.332376	-2.111226	0.026846
C	2.381973	-1.232427	0.214126
C	3.669221	-1.502876	-0.254977
C	4.789597	-0.665288	-0.119682
N	5.986771	-1.014280	-0.601987
C	4.884699	0.703750	0.546467
C	3.996856	1.743200	-0.168378
C	6.345556	1.027525	0.334690
C	4.568420	0.621633	2.054250
C	6.268618	-2.261183	-1.300314
C	6.960576	-0.014788	-0.347832
C	8.305731	-0.002422	-0.685881
C	9.038557	1.120193	-0.307850
C	8.438448	2.177133	0.378174
C	7.083245	2.138743	0.705703
H	-9.912183	0.841451	1.336394
H	-9.867324	1.783681	-0.946883
H	-7.811153	1.719542	-2.307202
H	-7.866900	-0.193607	2.301592
H	-4.448926	-2.336930	1.338023
H	-6.074855	-2.076965	1.990871
H	-5.856282	-2.406502	0.263639
H	-5.366766	0.205477	2.957129
H	-3.725578	-0.001581	2.324140
H	-4.668739	1.434649	1.888506
H	-5.854467	1.439819	-3.122971
H	-4.193532	1.709133	-2.565556
H	-4.674837	0.120442	-3.216940
H	-2.886225	-0.047054	-1.706637
H	-2.589600	-1.323694	1.094851
H	-0.689906	-0.906245	-1.286185
H	-0.291224	-2.221302	1.483047
H	1.554374	-3.044139	-0.500248
H	2.168530	-0.307172	0.737408
H	3.811070	-2.448457	-0.770133
H	2.933761	1.523500	-0.049157
H	4.187650	2.729996	0.262211
H	4.225653	1.788664	-1.236768
H	3.521774	0.370527	2.239118
H	5.197912	-0.123049	2.549246
H	4.767904	1.593977	2.512877
H	5.664055	-2.327702	-2.207878
H	7.319241	-2.287937	-1.578147
H	6.056733	-3.112084	-0.648679
H	8.787487	-0.814611	-1.218204
H	10.093770	1.169462	-0.552893
H	9.034384	3.038345	0.659718
H	6.622055	2.963911	1.239704

*Z*1 – Transition State 1 (S_1)

C	-0.109110	-0.245670	0.319467
C	-0.002074	-0.311734	1.708197
C	1.240089	-0.285817	2.340248
C	2.361678	-0.191637	1.527784
C	2.272239	-0.124866	0.140662
C	1.034047	-0.151326	-0.476029
C	3.661033	-0.029034	-0.452607
C	3.818570	1.297234	-1.223669
C	3.938341	-1.247306	-1.356458
C	4.524630	-0.054075	0.808368
N	3.723803	-0.146603	1.891544
C	4.205463	-0.194872	3.259420
C	5.912826	0.004700	0.921713
C	6.819680	0.102053	-0.135287
C	8.200070	0.150506	0.036213
C	9.097586	0.242574	-1.033473
C	10.474304	0.306137	-0.890565
C	11.365093	0.448779	-1.980985
C	12.729658	0.459965	-1.842723
C	13.656224	0.422876	-2.956101
N	14.529902	1.429363	-3.226916
C	13.924544	-0.759312	-3.871879
C	14.338798	-2.005927	-3.060423
C	15.072752	-0.218476	-4.699373
C	12.702886	-1.098739	-4.751620
C	14.567802	2.726586	-2.588550
C	15.378762	1.078376	-4.281793
C	16.383392	1.837341	-4.869606
C	17.100044	1.243655	-5.907239
C	16.813809	-0.053523	-6.333889
C	15.794384	-0.794141	-5.729509
H	-1.088849	-0.267872	-0.145567
H	-0.899890	-0.384751	2.312922
H	1.302627	-0.338341	3.421523
H	0.948440	-0.099953	-1.557719

H	4.819874	1.407633	-1.647335
H	3.098383	1.323715	-2.046944
H	3.622831	2.153870	-0.572767
H	3.219573	-1.255784	-2.181353
H	4.943370	-1.218347	-1.784647
H	3.827489	-2.181508	-0.798979
H	3.359336	-0.266881	3.939466
H	4.846802	-1.069146	3.405644
H	4.767660	0.713346	3.496233
H	6.336842	-0.027940	1.921588
H	6.449852	0.140258	-1.156118
H	8.601603	0.115041	1.047921
H	8.685689	0.274655	-2.042668
H	10.898803	0.242890	0.110598
H	10.939983	0.517966	-2.980881
H	13.137111	0.423541	-0.826127
H	13.519463	-2.329258	-2.410112
H	14.582363	-2.829799	-3.739134
H	15.217910	-1.799036	-2.443556
H	11.873653	-1.459629	-4.134456
H	12.371102	-0.225948	-5.321812
H	12.965983	-1.890703	-5.460922
H	15.547451	2.892236	-2.128707
H	14.383743	3.516561	-3.324822
H	13.794541	2.773749	-1.821518
H	16.610474	2.845551	-4.538958
H	17.895038	1.803190	-6.389461
H	17.387243	-0.490139	-7.144770
H	15.573953	-1.802768	-6.067809

*Z*1 – Transition State 2 (S_1)

C	-0.099058	0.131850	0.198387
C	-0.025982	0.267503	1.584253
C	1.201495	0.304529	2.243985
C	2.343358	0.200489	1.461748
C	2.287921	0.065037	0.078042
C	1.064120	0.029328	-0.566362
C	3.691993	-0.022841	-0.479223
C	3.959096	1.157018	-1.435517
C	3.900277	-1.380637	-1.180308
C	4.525124	0.086130	0.797608
N	3.698090	0.208290	1.856823
C	4.146786	0.332951	3.231312
C	5.912076	0.071179	0.943948
C	6.843438	-0.054068	-0.087318
C	8.221446	-0.057831	0.113227
C	9.140797	-0.180217	-0.933479
C	10.516206	-0.209682	-0.762846
C	11.428657	-0.394243	-1.827859
C	12.791493	-0.357068	-1.672829
C	13.736339	-0.446419	-2.766574
N	13.932849	0.530109	-3.698002
C	14.698165	-1.594492	-3.002265
C	15.650078	-1.771861	-1.798787
C	15.434121	-1.099888	-4.229583
C	13.943099	-2.912713	-3.270104
C	13.292282	1.825073	-3.743714
C	14.955292	0.164409	-4.580794
C	15.463216	0.883638	-5.656105
C	16.477258	0.281602	-6.399206
C	16.959725	-0.985506	-6.070923
C	16.438071	-1.685354	-4.979537
H	-1.068018	0.105866	-0.288515
H	-0.939114	0.346143	2.164814
H	1.237629	0.411005	3.322485
H	1.004814	-0.076257	-1.645867
H	4.974156	1.134020	-1.839742
H	3.259588	1.106217	-2.275269
H	3.811515	2.113930	-0.927219
H	3.198255	-1.466739	-2.015068
H	4.912572	-1.484854	-1.578568
H	3.713257	-2.209349	-0.491792
H	3.283432	0.405483	3.889257
H	4.732766	-0.544629	3.519904
H	4.755500	1.233997	3.352094
H	6.311772	0.164701	1.949984
H	6.496600	-0.153237	-1.112097
H	8.601389	0.036514	1.129423
H	8.749066	-0.271637	-1.947105
H	10.921069	-0.090883	0.241313
H	11.023212	-0.531386	-2.829938
H	13.185460	-0.260475	-0.655848
H	15.090210	-2.064586	-0.904037
H	16.379198	-2.559862	-2.013614
H	16.196939	-0.848183	-1.588397
H	13.354434	-3.204045	-2.394235
H	13.270539	-2.811726	-4.126905
H	14.655488	-3.715666	-3.485803
H	12.465807	1.845971	-3.033763
H	14.005274	2.616284	-3.486265
H	12.906060	2.010766	-4.750749
H	15.093635	1.870573	-5.914282
H	16.897705	0.811867	-7.247583
H	17.751421	-1.428878	-6.665575
H	16.821970	-2.668857	-4.723369

*Z*2 – Transition State 1 (S_1)

C	9.058110	1.286069	0.147021
C	9.305662	0.201880	-0.693707
C	8.279324	-0.651261	-1.096735
C	7.001695	-0.376335	-0.626439
C	6.738759	0.703016	0.214116
C	7.765107	1.542609	0.607453
C	5.263189	0.735368	0.551217
C	4.630604	2.046155	0.042359
C	5.062580	0.565080	2.070597
C	4.753423	-0.478268	-0.226038
N	5.798114	-1.061034	-0.870221

C	5.679876	-2.240529	-1.702174
C	3.460066	-0.977022	-0.316810
C	2.323890	-0.453943	0.307694
C	1.044374	-1.000600	0.163247
C	-0.086378	-0.474648	0.763236
C	-1.378411	-1.050458	0.648034
C	-2.500197	-0.547309	1.243757
C	-3.816582	-1.163039	1.090088
C	-4.923610	-0.545955	0.572262
N	-6.152590	-1.160020	0.511530
C	-5.041035	0.852270	-0.028690
C	-4.794849	1.955307	1.021481
C	-6.487072	0.863260	-0.464697
C	-4.091510	1.027180	-1.230796
C	-6.416293	-2.495146	1.005439
C	-7.093468	-0.349082	-0.114636
C	-8.433262	-0.615548	-0.397819
C	-9.157016	0.380013	-1.042639
C	-8.564630	1.597261	-1.393996
C	-7.221834	1.844245	-1.104744
H	9.875641	1.933827	0.445114
H	10.315295	0.013855	-1.044088
H	8.492959	-1.489219	-1.751126
H	7.572749	2.387456	1.262907
H	3.558092	2.087994	0.248609
H	5.105278	2.896337	0.541910
H	4.778886	2.158019	-1.035330
H	5.543884	1.395921	2.595697
H	4.004497	0.559125	2.344298
H	5.514210	-0.366980	2.421514
H	6.657929	-2.504981	-2.099458
H	5.301405	-3.084573	-1.116673
H	5.002497	-2.050218	-2.540910
H	3.306515	-1.860183	-0.931870
H	2.413675	0.426036	0.938139
H	0.934199	-1.888044	-0.459931
H	0.016603	0.419177	1.378364
H	-1.472261	-1.945850	0.033653
H	-2.411604	0.348826	1.856582
H	-3.917113	-2.197527	1.417035
H	-3.745890	1.980544	1.326011
H	-5.041151	2.930000	0.589020
H	-5.418314	1.807518	1.907770
H	-3.047618	0.965090	-0.912842
H	-4.272758	0.260645	-1.989485
H	-4.257108	2.007947	-1.687284
H	-6.043721	-2.591787	2.029170
H	-7.489600	-2.676953	1.011355
H	-5.930894	-3.245297	0.372046
H	-8.906278	-1.556508	-0.140413
H	-10.201296	0.205443	-1.279239
H	-9.155630	2.354607	-1.897746
H	-6.764887	2.789902	-1.381242

*Z*2 – Transition State 2 (S_1)

C	0.078439	0.045549	0.460883
C	0.243123	0.113803	1.844061
C	1.511077	0.142944	2.421725
C	2.602560	0.100933	1.562834
C	2.453922	0.033083	0.178817
C	1.190197	0.004721	-0.382713
C	3.817851	0.001917	-0.476575
C	3.996828	1.232566	-1.388845
C	4.000476	-1.312863	-1.261981
C	4.735179	0.062997	0.743735
N	3.974016	0.116015	1.867649
C	4.514108	0.178487	3.210321
C	6.125333	0.068510	0.793144
C	6.993395	0.017552	-0.300307
C	8.388493	0.015127	-0.176087
C	9.256081	-0.049711	-1.252934
C	10.667837	-0.006282	-1.121592
C	11.542027	-0.067985	-2.173414
C	12.986506	-0.050351	-2.062895
C	13.772505	0.926366	-1.502253
N	15.131696	0.760416	-1.362337
C	13.409644	2.354476	-1.080496
C	12.717159	3.104464	-2.235200
C	14.779448	2.929175	-0.796074
C	12.542825	2.434459	0.194934
C	15.839069	-0.472175	-1.638383
C	15.752808	1.940847	-0.970103
C	17.109837	2.192828	-0.769519
C	17.466685	3.478183	-0.378523
C	16.503568	4.474916	-0.196182
C	15.150345	4.202188	-0.404106
H	-0.920484	0.024021	0.038416
H	-0.630051	0.144982	2.487503
H	1.617520	0.196420	3.499599
H	1.060779	-0.048476	-1.460083
H	4.983617	1.252207	-1.858206
H	3.244623	1.207582	-2.183283
H	3.865092	2.159590	-0.823897
H	3.249652	-1.368517	-2.056180
H	4.988354	-1.377043	-1.725024
H	3.869617	-2.179399	-0.607915
H	3.697973	0.202428	3.929779
H	5.132544	-0.701552	3.413893
H	5.118505	1.082450	3.337417
H	6.590458	0.115298	1.774720
H	6.589456	-0.030248	-1.307369
H	8.812577	0.065504	0.826930
H	8.839749	-0.111955	-2.258027
H	11.072870	0.054927	-0.113506
H	11.135966	-0.264544	-3.167113
H	13.499932	-0.893955	-2.524467
H	11.750598	2.647022	-2.463892
H	12.544379	4.145476	-1.944744

H	13.334355	3.097629	-3.137803
H	11.505825	2.171858	-0.020044
H	12.926946	1.779854	0.983042
H	12.556678	3.462004	0.571500
H	15.993455	-0.605890	-2.715015
H	16.808182	-0.452702	-1.140308
H	15.268605	-1.318004	-1.247105
H	17.869351	1.433897	-0.920506
H	18.514195	3.710249	-0.217166
H	16.812539	5.469754	0.106485
H	14.405809	4.980690	-0.264210

*Z*3 – Transition State 1 (S_1)

C	-9.078522	-1.428030	-0.190682
C	-9.254403	-0.353610	-1.062730
C	-8.218551	0.539348	-1.329468
C	-7.004566	0.312971	-0.691846
C	-6.813644	-0.755315	0.183131
C	-7.850430	-1.634285	0.440314
C	-5.398070	-0.726089	0.716635
C	-4.643602	-2.009506	0.310466
C	-5.409015	-0.545646	2.249751
C	-4.842236	0.502003	0.008291
N	-5.805673	1.041911	-0.777327
C	-5.623669	2.221616	-1.599707
C	-3.558029	1.070568	0.108104
C	-2.512581	0.613421	0.876277
C	-1.200092	1.258796	0.857651
C	-0.042873	0.609746	0.544461
C	1.244894	1.212583	0.630355
C	2.417938	0.536607	0.375918
C	3.689210	1.142102	0.430891
C	4.902853	0.527217	0.167358
N	6.082511	1.195661	0.252321
C	5.179990	-0.919583	-0.242247
C	4.744405	-1.915476	0.851732
C	6.688536	-0.907602	-0.363170
C	4.540219	-1.259834	-1.603261
C	6.196938	2.592706	0.619330
C	7.171335	0.363830	-0.059783
C	8.527288	0.665904	-0.088538
C	9.402164	-0.361523	-0.436494
C	8.934048	-1.639045	-0.742722
C	7.566731	-1.919264	-0.707015
H	-9.903019	-2.107074	-0.001215
H	-10.215268	-0.205445	-1.544551
H	-8.376292	1.369884	-2.008711
H	-7.715518	-2.470452	1.120618
H	-3.605666	-1.992093	0.652836
H	-5.135561	-2.878036	0.758991
H	-4.647820	-2.137644	-0.775611
H	-5.907875	-1.403047	2.711788
H	-4.398050	-0.480275	2.659150
H	-5.954542	0.359159	2.531472
H	-6.535117	2.416857	-2.161098
H	-5.402960	3.093445	-0.975618
H	-4.804768	2.067270	-2.309235
H	-3.368862	1.959608	-0.488980
H	-2.620700	-0.272723	1.495007
H	-1.142942	2.313063	1.138244
H	-0.092538	-0.438213	0.249329
H	1.294428	2.261985	0.919235
H	2.339381	-0.514659	0.113433
H	3.706580	2.193315	0.706462
H	3.659351	-1.931295	0.981166
H	5.066440	-2.923547	0.573062
H	5.202579	-1.665078	1.812576
H	3.448449	-1.252656	-1.553721
H	4.855214	-0.548303	-2.371629
H	4.858386	-2.259801	-1.913829
H	5.788930	2.757475	1.621535
H	7.245153	2.884681	0.619055
H	5.659173	3.220448	-0.098276
H	8.911486	1.652442	0.146001
H	10.467563	-0.158492	-0.468270
H	9.637863	-2.419933	-1.010294
H	7.203533	-2.914955	-0.945560

*Z*3 – Transition State 2 (S_1)

C	-8.800265	-1.296675	0.544441
C	-8.357065	-2.247717	-0.374585
C	-7.081679	-2.176891	-0.931506
C	-6.273292	-1.118401	-0.534610
C	-6.700095	-0.162447	0.385987
C	-7.968623	-0.244217	0.931550
C	-5.597557	0.848440	0.615018
C	-5.163280	0.846844	2.095508
C	-6.061346	2.252815	0.171865
C	-4.505572	0.302890	-0.294950
N	-4.963031	-0.803089	-0.933344
C	-4.200320	-1.567065	-1.899482
C	-3.189727	0.774318	-0.474262
C	-2.632981	1.899785	0.083785
C	-1.247301	2.285147	-0.172550
C	-0.171308	1.494583	0.097904
C	1.166386	1.835509	-0.258415
C	2.243428	1.003365	-0.048682
C	3.569318	1.357743	-0.368903
C	4.698870	0.581491	-0.162945
N	5.940520	1.011838	-0.505969
C	4.807662	-0.818172	0.442680
C	4.343547	-0.840194	1.913027
C	6.299745	-1.058351	0.362975
C	4.056151	-1.866020	-0.402923
C	6.214296	2.302801	-1.104812
C	6.921593	0.051679	-0.204982
C	8.295784	0.109112	-0.402273
C	9.043437	-0.998150	-0.006100

C	8.435461	-2.116293	0.564237
C	7.052469	-2.151423	0.752358
H	-9.798179	-1.377132	0.962168
H	-9.013974	-3.061666	-0.663316
H	-6.752934	-2.929792	-1.639443
H	-8.314674	0.494469	1.648988
H	-4.333999	1.535625	2.274829
H	-6.005038	1.157931	2.721646
H	-4.855453	-0.154275	2.410005
H	-6.920768	2.558821	0.776318
H	-5.273259	2.998873	0.301768
H	-6.366168	2.250540	-0.878344
H	-4.850285	-2.297701	-2.377573
H	-3.801173	-0.903836	-2.672351
H	-3.373086	-2.094513	-1.412298
H	-2.541791	0.189315	-1.123107
H	-3.224866	2.564577	0.707205
H	-1.072434	3.261069	-0.631042
H	-0.339830	0.533072	0.582252
H	1.334868	2.807331	-0.721223
H	2.044145	0.032950	0.397254
H	3.707438	2.338682	-0.816060
H	3.272282	-0.644371	2.005501
H	4.546095	-1.826074	2.342646
H	4.882300	-0.094194	2.503784
H	2.975200	-1.705635	-0.384809
H	4.391338	-1.841326	-1.443573
H	4.256615	-2.865091	-0.003888
H	5.885713	3.107733	-0.439969
H	7.284455	2.406200	-1.272343
H	5.699304	2.393898	-2.066338
H	8.787292	0.968773	-0.844136
H	10.119286	-0.985247	-0.146181
H	9.042209	-2.964192	0.863614
H	6.580500	-3.023029	1.196992

*Z*4 – Transition State 1 (S_1)

C	9.111922	1.323779	-0.009741
C	9.329023	0.215169	-0.827199
C	8.291253	-0.654325	-1.158684
C	7.033897	-0.370099	-0.640960
C	6.801207	0.733512	0.177122
C	7.839107	1.589385	0.498898
C	5.341330	0.770677	0.574797
C	4.680279	2.060115	0.045674
C	5.201110	0.654681	2.106320
C	4.808660	-0.470589	-0.136541
N	5.824570	-1.067660	-0.809270
C	5.678913	-2.274915	-1.596945
C	3.509851	-0.981517	-0.151821
C	2.402487	-0.444132	0.491760
C	1.122997	-1.053320	0.461977
C	0.026467	-0.558025	1.109340
C	-1.294953	-1.182744	1.034927
C	-2.418141	-0.530764	0.611741
C	-3.707369	-1.129154	0.637399
C	-4.898935	-0.529067	0.278049
N	-6.085171	-1.198871	0.315336
C	-5.150405	0.898308	-0.206679
C	-4.767459	1.940868	0.862966
C	-6.649501	0.879902	-0.409336
C	-4.439646	1.179278	-1.546884
C	-6.221574	-2.580704	0.728683
C	-7.149634	-0.382792	-0.090315
C	-8.503231	-0.688189	-0.187696
C	-9.355338	0.324618	-0.620224
C	-8.869418	1.592627	-0.942424
C	-7.506612	1.877039	-0.837760
H	9.938183	1.983647	0.232444
H	10.323569	0.020961	-1.215205
H	8.480837	-1.511441	-1.795444
H	7.671083	2.453306	1.135839
H	3.616644	2.103375	0.293100
H	5.168834	2.929418	0.496470
H	4.785335	2.135054	-1.040262
H	5.692592	1.509094	2.581739
H	4.154017	0.648453	2.419342
H	5.675994	-0.259383	2.473422
H	6.637810	-2.538254	-2.038951
H	5.345452	-3.105039	-0.966067
H	4.954677	-2.119188	-2.402825
H	3.341982	-1.891965	-0.722287
H	2.496387	0.470844	1.068980
H	1.017594	-1.972073	-0.114878
H	0.119650	0.365787	1.681005
H	-1.390553	-2.220364	1.361595
H	-2.310946	0.495721	0.274561
H	-3.743469	-2.159534	0.981973
H	-3.690688	1.954627	1.049395
H	-5.064419	2.937352	0.521832
H	-5.278980	1.737433	1.807827
H	-3.352132	1.177464	-1.439712
H	-4.713966	0.433843	-2.298525
H	-4.741202	2.163896	-1.917205
H	-5.888192	-2.702786	1.764180
H	-7.265533	-2.879949	0.662020
H	-5.629157	-3.232079	0.078442
H	-8.900321	-1.666694	0.058446
H	-10.417246	0.119475	-0.706684
H	-9.556787	2.362363	-1.276661
H	-7.130560	2.864645	-1.089215

*Z*4 – Transition State 2 (S_1)

C	-8.789899	-0.758033	0.622326
C	-8.532127	-1.847014	-0.209408
C	-7.269957	-2.051392	-0.764623
C	-6.281669	-1.125626	-0.454999
C	-6.523515	-0.033202	0.375264

C	-7.780308	0.159317	0.919882
C	-5.258229	0.785129	0.518332
C	-4.813158	0.829536	1.994135
C	-5.478921	2.204328	-0.045024
C	-4.286482	-0.018976	-0.341604
N	-4.938352	-1.086323	-0.869430
C	-4.324154	-2.060343	-1.747923
C	-2.937229	0.227399	-0.600783
C	-2.185763	1.295779	-0.129648
C	-0.800543	1.441628	-0.402989
C	-0.034773	2.477812	0.046810
C	1.391518	2.598621	-0.246536
C	2.312303	1.639467	0.062979
C	3.674682	1.721405	-0.337607
C	4.647576	0.762880	-0.131027
N	5.937715	0.944260	-0.531312
C	4.522434	-0.607597	0.533365
C	4.137739	-0.486525	2.022747
C	5.937635	-1.126379	0.405175
C	3.543893	-1.524671	-0.227512
C	6.419586	2.140627	-1.191845
C	6.731849	-0.168641	-0.226365
C	8.085112	-0.376454	-0.473311
C	8.628423	-1.590750	-0.062559
C	7.846228	-2.558062	0.570570
C	6.489957	-2.328371	0.808563
H	-9.781632	-0.623467	1.040797
H	-9.326139	-2.552407	-0.431667
H	-7.089658	-2.905711	-1.407821
H	-7.982065	1.007515	1.568024
H	-3.882150	1.387694	2.121046
H	-5.587082	1.320070	2.592424
H	-4.666751	-0.180138	2.387931
H	-6.265173	2.704123	0.529054
H	-4.573897	2.813494	0.019065
H	-5.794175	2.164029	-1.091357
H	-5.070174	-2.786834	-2.063998
H	-3.920331	-1.567771	-2.638068
H	-3.517449	-2.589621	-1.230203
H	-2.416709	-0.485397	-1.236245
H	-2.645123	2.055399	0.495275
H	-0.324379	0.674607	-1.014189
H	-0.503192	3.276745	0.622603
H	1.727983	3.495786	-0.770311
H	1.964503	0.769839	0.611539
H	3.961068	2.628867	-0.863075
H	3.126379	-0.093508	2.152050
H	4.178500	-1.475846	2.488760
H	4.833381	0.168987	2.553884
H	2.516899	-1.154343	-0.173374
H	3.826539	-1.609393	-1.280554
H	3.566587	-2.525835	0.213795
H	6.247685	3.017370	-0.559914
H	7.488106	2.049965	-1.375911
H	5.909077	2.278981	-2.150297
H	8.709147	0.361747	-0.964544
H	9.681018	-1.784657	-0.240219
H	8.296952	-3.494910	0.880105
H	5.883439	-3.082214	1.302395

Cy$^{\bullet+}$ (D_0)

C	-9.138550	1.500531	0.000384
C	-9.513627	0.153875	0.000335
C	-8.560517	-0.859419	0.000259
C	-7.229513	-0.461198	0.000234
C	-6.836617	0.872824	0.000281
C	-7.794658	1.872828	0.000357
C	-5.328951	0.956074	0.000235
C	-4.830931	1.663603	1.279314
C	-4.831014	1.663718	-1.278812
C	-4.958028	-0.520719	0.000156
N	-6.059278	-1.263049	0.000160
C	-6.089903	-2.724904	0.000097
C	-3.666986	-1.120027	0.000084
C	-2.466524	-0.445478	0.000077
C	-1.224207	-1.115235	0.000001
C	0.000000	-0.450560	-0.000010
C	1.224207	-1.115235	-0.000086
C	2.466524	-0.445478	-0.000100
C	3.666986	-1.120027	-0.000176
C	4.958028	-0.520719	-0.000197
N	6.059278	-1.263049	-0.000272
C	5.328951	0.956074	-0.000145
C	4.830923	1.663718	-1.279157
C	6.836617	0.872824	-0.000207
C	4.831022	1.663603	1.278970
C	6.089903	-2.724904	-0.000339
C	7.229513	-0.461198	-0.000282
C	8.560517	-0.859419	-0.000353
C	9.513627	0.153875	-0.000345
C	9.138550	1.500531	-0.000270
C	7.794658	1.872828	-0.000200
H	-9.906304	2.266651	0.000443
H	-10.565548	-0.109341	0.000357
H	-8.869540	-1.898399	0.000221
H	-7.514224	2.921587	0.000395
H	-3.740655	1.726630	1.315025
H	-5.226084	2.682789	1.296190
H	-5.180577	1.149756	2.178877
H	-5.226165	2.682907	-1.295568
H	-3.740740	1.726744	-1.314590
H	-5.180722	1.149955	-2.178399
H	-7.123484	-3.060692	0.000136
H	-5.597490	-3.107003	-0.896549
H	-5.597397	-3.107082	0.896658
H	-3.625763	-2.205683	0.000029
H	-2.447817	0.639986	0.000130
H	-1.225905	-2.204080	-0.000052
H	0.000000	0.638938	0.000043

H	1.225905	-2.204080	-0.000139
H	2.447817	0.639986	-0.000047
H	3.625763	-2.205683	-0.000227
H	3.740647	1.726748	-1.314855
H	5.226077	2.682906	-1.295943
H	5.180563	1.149953	-2.178768
H	3.740748	1.726626	1.314760
H	5.180735	1.149759	2.178508
H	5.226173	2.682790	1.295815
H	5.597413	-3.107002	-0.896943
H	7.123484	-3.060692	-0.000389
H	5.597474	-3.107083	0.896264
H	8.869540	-1.898398	-0.000412
H	10.565548	-0.109341	-0.000399
H	9.906304	2.266651	-0.000266
H	7.514224	2.921587	-0.000142

O_2 (T_0)

O	0.000000	0.000000	0.002401
O	0.000000	0.000000	1.207599

[Cy–O_2] (T_0)

C	7.984241	-1.969266	0.412819
C	7.114415	-0.914828	0.198799
C	7.610297	0.366940	-0.007457
C	8.968446	0.644529	-0.008940
C	9.837649	-0.425322	0.207648
C	9.357149	-1.716304	0.416192
N	6.518757	1.245925	-0.198402
C	5.338452	0.596789	-0.126492
C	5.601609	-0.888309	0.141896
C	4.125012	1.255269	-0.284021
C	2.863187	0.659061	-0.225088
C	1.662639	1.337539	-0.386108
C	0.434891	0.682874	-0.317185
C	-0.813462	1.281947	-0.466332
C	-1.991014	0.550502	-0.383135
C	-3.270692	1.091463	-0.523432
C	-4.462185	0.380909	-0.444106
C	-4.679069	-1.114748	-0.194820
C	-6.190327	-1.202894	-0.225953
C	-6.724945	0.057172	-0.465491
N	-5.661480	0.979843	-0.592924
C	-7.027809	-2.291401	-0.058466
C	-8.407760	-2.094170	-0.134651
C	-8.927207	-0.823926	-0.374956
C	-8.090865	0.279699	-0.545717
C	-4.076754	-1.969577	-1.327005
C	-4.152318	-1.546435	1.187339
C	-5.835072	2.399392	-0.832317
C	6.648062	2.669810	-0.442007
C	5.014553	-1.333852	1.495326
C	5.101139	-1.772795	-1.016593
H	-9.080531	-2.935210	-0.005542
H	-10.001721	-0.684563	-0.431230
H	-8.513774	1.260843	-0.730970
H	-6.623759	-3.282212	0.129318
H	-2.985328	-1.913926	-1.346230
H	-4.359777	-3.016131	-1.179298
H	-4.454940	-1.649603	-2.301944
H	-4.444151	-2.583795	1.376865
H	-3.062835	-1.486231	1.248981
H	-4.576199	-0.923006	1.978887
H	-6.897038	2.630083	-0.887263
H	-5.394596	2.977557	-0.014673
H	-5.364408	2.687575	-1.776955
H	-3.329475	2.159598	-0.710722
H	-1.887814	-0.515404	-0.195757
H	-0.865720	2.352689	-0.653079
H	0.452955	-0.392732	-0.130230
H	1.678973	2.409626	-0.571685
H	2.794453	-0.410224	-0.041488
H	4.148858	2.325051	-0.469754
H	3.921825	-1.312931	1.496029
H	5.333024	-2.358962	1.707248
H	5.371566	-0.692766	2.306146
H	4.011191	-1.766335	-1.097136
H	5.518742	-1.439968	-1.970903
H	5.421307	-2.805252	-0.847100
H	6.147489	3.238002	0.347720
H	7.701591	2.941846	-0.450953
H	6.211518	2.931539	-1.410539
H	9.361917	1.642212	-0.169350
H	10.907113	-0.242568	0.212713
H	10.054948	-2.530028	0.582226
H	7.610527	-2.976307	0.575510
O	-5.578147	1.607752	2.494379
O	-6.781907	1.597341	2.424616

[Cy–O_2] (T_0) (Resonance Structure 1)

C	8.103764	-1.219132	-0.408195
C	7.063811	-0.351361	-0.160193
C	7.299452	1.020603	-0.030661
C	8.576007	1.546445	-0.142458
C	9.614768	0.663026	-0.390885
C	9.386331	-0.698614	-0.522854
N	6.089102	1.664248	0.214645
C	5.067771	0.790577	0.263442
C	5.589021	-0.610206	0.009091
C	3.760580	1.192983	0.513954
C	2.634650	0.397493	0.557399
C	1.352429	0.927122	0.752659
C	0.205007	0.146868	0.746075
C	-1.055538	0.725062	0.690622
C	-2.219753	-0.010552	0.433104
C	-3.459564	0.585128	0.331592
C	-4.666595	-0.029554	0.017316

C	-4.931625	-1.496199	-0.260684
C	-6.418735	-1.487621	-0.503904
C	-6.887853	-0.175698	-0.388247
N	-5.816591	0.660407	-0.083724
C	-7.284361	-2.516049	-0.802327
C	-8.628926	-2.217605	-0.981690
C	-9.089319	-0.914668	-0.863273
C	-8.227508	0.128990	-0.564506
C	-4.203811	-1.970753	-1.526914
C	-4.600511	-2.383835	0.946283
C	-5.936483	2.083611	0.085651
C	5.959520	3.087175	0.379041
C	5.351595	-1.542690	1.204287
C	5.015556	-1.198574	-1.288320
H	-9.323856	-3.008481	-1.215418
H	-10.137911	-0.705764	-1.005603
H	-8.608926	1.133693	-0.475639
H	-6.932090	-3.531336	-0.895498
H	-3.126417	-1.930605	-1.406146
H	-4.493650	-2.994518	-1.747626
H	-4.476593	-1.345511	-2.373971
H	-4.914716	-3.403557	0.740932
H	-3.536938	-2.385228	1.160601
H	-5.130417	-2.032695	1.828907
H	-5.697761	2.371540	1.111711
H	-5.263963	2.606601	-0.596588
H	-6.955929	2.388019	-0.132489
H	-3.483022	1.653144	0.498006
H	-2.103679	-1.069601	0.261471
H	-1.155790	1.776086	0.922857
H	0.298744	-0.917630	0.573176
H	1.263802	1.979585	0.983048
H	2.706293	-0.665580	0.386380
H	3.596062	2.249399	0.674728
H	5.842328	-2.494840	1.021858
H	5.770273	-1.108743	2.109569
H	4.293932	-1.722814	1.365572
H	5.483988	-2.158650	-1.487306
H	3.942035	-1.340202	-1.221380
H	5.221399	-0.535089	-2.125163
H	5.625307	3.329952	1.390017
H	6.922821	3.559016	0.208913
H	5.243327	3.490114	-0.339248
H	8.778294	2.600845	-0.041803
H	10.619138	1.045484	-0.482569
H	10.215054	-1.361039	-0.716360
H	7.932206	-2.279067	-0.511618
O	-0.494828	1.582345	-1.376274
O	0.754707	1.687895	-1.347116

[Cy–O$_2$] (T_0) (Resonance Structure 2)

C	7.735033	-1.557122	-0.003330
C	6.850528	-0.493816	0.031886
C	7.329242	0.808666	0.107399
C	8.684091	1.098748	0.149967
C	9.568150	0.019782	0.113919
C	9.105011	-1.291990	0.038328
N	6.225304	1.692716	0.129035
C	5.053534	1.027415	0.070864
C	5.336680	-0.477176	0.001328
C	3.830153	1.686520	0.077628
C	2.581819	1.063992	0.019727
C	1.358809	1.721555	0.024990
C	0.165090	1.007237	-0.036393
C	-1.122883	1.536294	-0.040040
C	-2.233124	0.704775	-0.105121
C	-3.560976	1.135885	-0.115058
C	-4.671911	0.303557	-0.179942
C	-4.726701	-1.226285	-0.253530
C	-6.221254	-1.465761	-0.298674
C	-6.889514	-0.248027	-0.256473
N	-5.930253	0.788895	-0.185706
C	-6.936755	-2.647736	-0.370716
C	-8.331176	-2.587312	-0.399602
C	-8.985459	-1.358056	-0.356571
C	-8.272705	-0.160666	-0.283919
C	-4.067817	-1.759460	-1.540818
C	-4.125273	-1.879773	1.006118
C	-6.255113	2.201173	-0.126134
C	6.334488	3.137041	0.203904
C	4.776100	-1.220651	1.229553
C	4.828691	-1.092814	-1.317159
H	-8.909548	-3.503253	-0.455882
H	-10.069696	-1.325235	-0.379749
H	-8.800152	0.786283	-0.251380
H	-6.427434	-3.606786	-0.404395
H	-4.249325	-2.835616	-1.619458
H	-4.492507	-1.277946	-2.426054
H	-2.986722	-1.599107	-1.546360
H	-3.045708	-1.723854	1.074842
H	-4.589629	-1.482161	1.912843
H	-4.308722	-2.958027	0.975322
H	-5.828236	2.726476	-0.985724
H	-7.335959	2.325476	-0.144770
H	-5.868728	2.641925	0.797727
H	-3.728782	2.207798	-0.068100
H	-2.029906	-0.361995	-0.151114
H	-1.259679	2.614439	0.008534
H	0.247587	-0.079038	-0.087042
H	1.331483	2.807820	0.077329
H	2.541274	-0.020800	-0.034540
H	3.835302	2.771157	0.131500
H	5.131205	-0.765317	2.158229
H	3.683253	-1.225775	1.244024
H	5.117664	-2.259943	1.208551
H	5.170681	-2.129888	-1.386036
H	3.737385	-1.093462	-1.377165
H	5.221195	-0.547851	-2.180193
H	5.879524	3.599293	-0.677245

H	5.840016	3.509713	1.105946
H	7.384516	3.419922	0.241467
H	9.064216	2.112636	0.208763
H	10.635519	0.211900	0.145543
H	9.814040	-2.112482	0.011636
H	7.374932	-2.580429	-0.062298
O	1.003204	-2.682886	-0.205378
O	-0.198655	-2.771197	-0.228574

[Cy–O$_2$] (T_0) (Resonance Structure 3)

C	8.130468	-1.515028	-0.127238
C	7.192462	-0.499254	-0.075492
C	7.603563	0.824341	0.027266
C	8.941537	1.182630	0.081239
C	9.879861	0.151287	0.028351
C	9.484906	-1.180778	-0.074402
N	6.456048	1.650346	0.061111
C	5.319863	0.927317	-0.015893
C	5.679840	-0.558479	-0.112924
C	4.065503	1.524867	-0.002518
C	2.842959	0.853287	-0.076222
C	1.602049	1.475800	-0.061617
C	0.414380	0.751740	-0.137307
C	-0.867339	1.296396	-0.129039
C	-2.003056	0.501064	-0.207355
C	-3.311497	0.988931	-0.204120
C	-4.464093	0.216188	-0.279180
C	-4.600117	-1.306762	-0.370067
C	-6.105361	-1.467053	-0.402796
C	-6.707481	-0.215820	-0.348033
N	-5.694668	0.767859	-0.278164
C	-6.883572	-2.609129	-0.464632
C	-8.273199	-2.474538	-0.469787
C	-8.860530	-1.212321	-0.415197
C	-8.084164	-0.054788	-0.353513
C	-3.981917	-1.854877	-1.671009
C	-4.020470	-2.004658	0.875433
C	-5.944738	2.193841	-0.193715
C	6.492200	3.096403	0.165789
C	5.151538	-1.350959	1.098560
C	5.207726	-1.171401	-1.445648
H	-8.900288	-3.358418	-0.515503
H	-9.941746	-1.122268	-0.418849
H	-8.559768	0.918588	-0.306885
H	-6.426738	-3.593933	-0.505311
H	-2.895263	-1.738701	-1.689502
H	-4.209602	-2.921599	-1.757551
H	-4.397437	-1.346745	-2.545689
H	-2.934046	-1.901973	0.935075
H	-4.455578	-1.594371	1.790396
H	-4.256887	-3.072190	0.834356
H	-5.562239	2.592496	0.750785
H	-5.465862	2.713276	-1.028896
H	-7.016181	2.377360	-0.241176
H	-3.428092	2.066569	-0.135953
H	-1.840893	-0.571764	-0.275899
H	-0.980209	2.376434	-0.058781
H	0.495260	-0.334762	-0.209037
H	1.553686	2.560481	0.011128
H	2.841474	-0.231352	-0.150448
H	4.021329	2.607560	0.071166
H	5.487817	-0.900885	2.036769
H	4.059753	-1.400000	1.112427
H	5.535402	-2.374689	1.055874
H	5.581972	-0.594101	-2.295675
H	5.594121	-2.191496	-1.531081
H	4.117820	-1.216496	-1.513543
H	7.526580	3.429994	0.218674
H	6.021857	3.553682	-0.709890
H	5.972782	3.425435	1.070778
H	9.268737	2.213431	0.160817
H	10.935873	0.397124	0.068244
H	10.235233	-1.963142	-0.113815
H	7.823512	-2.554051	-0.207572
O	-7.397986	-1.466133	2.788884
O	-6.751676	-0.450417	2.850062

10. References

1. Štacková, L., Štacko, P. & Klán, P. Approach to a Substituted Heptamethine Cyanine Chain by the Ring Opening of Zincke Salts. *J. Am. Chem. Soc.* **141**, 7155–7162 (2019).
2. Lv, C., Wan, C., Liu, S., Lan, Y. & Li, Y. Aryne Trifunctionalization Enabled by 3-Silylaryne as a 1,2-Benzdiyne Equivalent. *Org. Lett.* **20**, 1919–1923 (2018).
3. Janeková, H. *et al.* Deuteration of heptamethine cyanine dyes enhances their emission efficacy. *Chem. Comm.* **60**, 1000–1003 (2023).
4. Feng, Y., Vinogradov, I. & Ge, N.-H. General noise suppression scheme with reference detection in heterodyne nonlinear spectroscopy. *Opt. Express.* **25**, 26262 (2017).
5. Liu, Y. *et al.* Sub-Millisecond Photoinduced Dynamics of Free and EL222-Bound FMN by Stimulated Raman and Visible Absorption Spectroscopies. *Biomol.* **13**, (2023).
6. Kloz, M., Weißenborn, J., Polívka, T., Frank, H. A. & Kennis, J. T. M. Spectral watermarking in femtosecond stimulated Raman spectroscopy: Resolving the nature of the carotenoid S∗ state. *PCCP* **18**, 14619–14628 (2016).
7. Janoš, J. *et al.* Conformational control of the photodynamics of a bilirubin dipyrrinone subunit: Femtosecond spectroscopy combined with nonadiabatic simulations. *J. Phys. Chem. A.* **124**, 10457–10471 (2020).
8. Madea, D. *et al.* Photochemistry of (Z)-Isovinylneoxanthobilirubic Acid Methyl Ester, a Bilirubin Dipyrrinone Subunit: Femtosecond Transient Absorption and Stimulated Raman Emission Spectroscopy. *J. Org. Chem.* **87**, 3089–3103 (2022).
9. Mullen, K. M. & Stokkum, I. H. M. V. The variable projection algorithm in time-resolved spectroscopy, microscopy and mass spectrometry applications. *Numer. Algorithms.* **51**, 319–340 (2009).
10. Mullen, K. M. & Van Stokkum, I. H. M. An R Package for Modeling Multi-way Spectroscopic Measurements. *J. Stat. Softw.* **18**, 1–46 (2007).
11. Kovalenko, S. A., Dobryakov, A. L., Ruthmann, J. & Ernsting, N. P. Femtosecond spectroscopy of condensed phases with chirped supercontinuum probing. *Phys. Rev. A.* **59**, 2369–2384 (1999).
12. Slavov, C., Hartmann, H. & Wachtveitl, J. Implementation and evaluation of data analysis strategies for time-resolved optical spectroscopy. *Anal. Chem.* **87**, 2328–2336 (2015).
13. Tutorials for Origin 9.0. https://www.originlab.com/pdfs/tutorials.pdf.
14. Chai, J. Da & Head-Gordon, M. Long-range corrected hybrid density functionals with damped atom-atom dispersion corrections. *PCCP* **10**, 6615–6620 (2008).
15. Jacquemin, D., Chibani, S., Le Guennic, B. & Mennucci, B. Solvent effects on cyanine derivatives: A PCM investigation. *J. Phys. Chem. A.* **118**, 5343–5348 (2014).
16. Marenich, A. V., Cramer, C. J. & Truhlar, D. G. Universal solvation model based on solute electron density and on a continuum model of the solvent defined by the bulk dielectric constant and atomic surface tensions. *J. Phys. Chem. B.* **113**, 6378–6396 (2009).
17. Zapata Trujillo, J. C. & McKemmish, L. K. Model Chemistry Recommendations for Scaled Harmonic Frequency Calculations: A Benchmark Study. *J. Phys. Chem. A.* **127**, 1715–1735 (2023).
18. Le Guennic, B. & Jacquemin, D. Taking Up the Cyanine Challenge with Quantum Tools. *Acc. Chem. Res.* **48**, 530–537 (2015).

19. Shao, Y. *et al.* Advances in molecular quantum chemistry contained in the Q-Chem 4 program package. *Mol. Phys.* **113**, 184–215 (2015).

20. Epifanovsky, E. *et al.* Software for the frontiers of quantum chemistry: An overview of developments in the Q-Chem 5 package. *J. Chem. Phys.* **155**, (2021).

21. R. D. Johnson. NIST Standard Reference Database Number 101, 2022. NIST Computational Chemistry Comparison and Benchmark Database. http://cccbdb.nist.gov/.

22. Frisch, M. J. ; *et al. Gaussian 16 Rev. A.03, Wallingford, CT*. (2016).

23. Sure, R. & Grimme, S. Comprehensive Benchmark of Association (Free) Energies of Realistic Host-Guest Complexes. *J. Chem. Theory. Comput.* **11**, 3785–3801 (2015).

24. Neese, F., Wennmohs, F., Becker, U. & Riplinger, C. The ORCA quantum chemistry program package. *J. Chem. Phys.* **152**, (2020).

25. Bannwarth, C., Ehlert, S. & Grimme, S. GFN2-xTB - An Accurate and Broadly Parametrized Self-Consistent Tight-Binding Quantum Chemical Method with Multipole Electrostatics and Density-Dependent Dispersion Contributions. *J. Chem. Theory. Comput.* **15**, 1652–1671 (2019).

26. Romero, N. A. & Nicewicz, D. A. Organic Photoredox Catalysis. *Chem. Rev.* **116**, 10075–10166 (2016).

27. Sellet, N., Clement-Comoy, L., Elhabiri, M., Cormier, M. & Goddard, J. P. Second generation of near-infrared cyanine-based photocatalysts for faster organic transformations. *Chem. Eur. J.* **29**, (2023).

28. AlNashef, I. M., Leonard, M. L., Kittle, M. C., Matthews, M. A. & Weidner, J. W. Electrochemical generation of superoxide in room-temperature ionic liquids. *Electrochem. Solid-State Lett.* **4**, (2001).

29. Lenhard, J. R. & Cameron, A. D. Electrochemistry and Electronic Spectra of Cyanine Dye Radicals in Acetonitrile. *J. Phys. Chem.* **97**, 4916–4925 (1993).

30. Belko, N. *et al.* Indotricarbocyanine dyes relevant for photodynamic therapy and their radicals: Substituent effects studied by optical and electrochemical methods. *Dyes Pigm.* **216**, (2023).

31. Fouassier, J.-P., Lougnot, D.-J. & Faure, J. Transient absorptions in a polymethine laser dye. *Chem. Phys. Lett.* **35**, 189–194 (1975).
